# Highly Enantioselective Catalysis by Enzyme Encapsulated in Metal Azolate Frameworks with Micelle-Controlled Pore Sizes

**DOI:** 10.1021/acscentsci.3c01432

**Published:** 2024-01-18

**Authors:** Hao Ren, Jian Yuan, Yi-Ming Li, Wen-Jing Li, Yi-Hang Guo, Yi-Bo Zhang, Bing-Hao Wang, Kaili Ma, Lu Peng, Guping Hu, Wen-Qi Wang, Hailong He, Lien-Yang Chou, Ming-Hua Zeng, Yue-Biao Zhang, Lin Cheng

**Affiliations:** † Jiangsu Engineering Laboratory of Smart Carbon-Rich Materials and Device, School of Chemistry and Chemical Engineering, Southeast University, Nanjing 211189, China; ‡ School of Physical Science and Technology, Shanghai Key Laboratory of High-Resolution Electron Microscopy, State Key Laboratory of Advanced Medical Materials and Devices, ShanghaiTech University, Shanghai 201210, China; § Avogadral Solutions, 3130 Grants Lake Boulevard #18641, Sugar Land, Texas 77496, United States; ∥ School of Chemistry and Chemical Engineering, Anhui University, Hefei 230601, China; ⊥ State Key Laboratory of Structural Chemistry, Fujian Institute of Research on the Structure of Matter, Chinese Academy of Sciences, Fujian 350002, China; # School of Chemistry and Pharmaceutical Sciences, State Key Laboratory for Chemistry and Molecular Engineering of Medicinal Resources, Guangxi Normal University, Guilin 541004, China; ¶ Analysis and Testing Center, Southeast University, Nanjing 211189, China; □ School of Chemistry, Sun Yat-Sen University, Guangzhou 510275, China

## Abstract

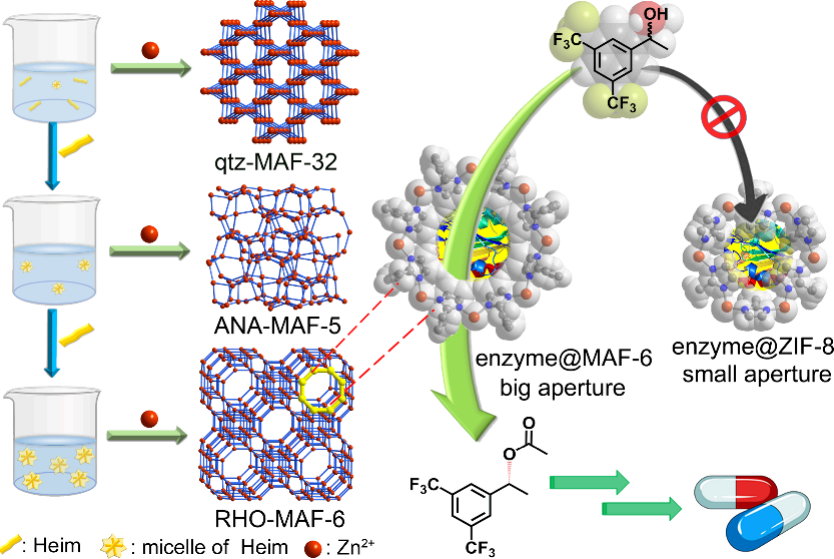

Encapsulating enzymes within metal–organic frameworks has enhanced their structural stability and interface tunability for catalysis. However, the small apertures of the frameworks restrict their effectiveness to small organic molecules. Herein, we present a green strategy directed by visible linker micelles for the aqueous synthesis of MAF-6 that enables enzymes for the catalytic asymmetric synthesis of chiral molecules. Due to the large pore aperture (7.6 Å), double the aperture size of benchmark ZIF-8 (3.4 Å), MAF-6 allows encapsulated enzyme BCL to access larger substrates and do so faster. Through the optimization of surfactants’ effect during synthesis, BCL@MAF-6-SDS (SDS = sodium dodecyl sulfate) displayed a catalytic efficiency (*K*_cat_/*K*_m_) that was 420 times greater than that of BCL@ZIF-8. This biocomposite efficiently catalyzed the synthesis of drug precursor molecules with 94–99% enantioselectivity and nearly quantitative yields. These findings represent a deeper understanding of de novo synthetic encapsulation of enzyme in MOFs, thereby unfolding the great potential of enzyme@MAF catalysts for asymmetric synthesis of organics and pharmaceuticals.

## Abstract 2

A micelle-oriented synthetic protocol for preparing enzyme@MAF catalysts is presented with practical applications in highly enatioselective chiral drug precursor synthesis.

## Introduction

Enzymes, the highly evolved biological catalysts capable of converting simple substances into complex chiral molecules through regiospecific and stereospecific reactions, play a crucial role in both biological processes and industrial production.^1^ However, the structural susceptibility of enzymes, rendering their activity and selectivity vulnerable to temperature and chemical environments, imposes limitations on their broader applicability.^2^ Immobilizing enzymes on solid materials, such as zeolites, mesoporous silica, and polymers, enhances stability and reusability^3−10^ but has minimal impact on enzyme structures.^11^ In contrast, metal–organic frameworks (MOFs), formed by periodic linkages of metal nodes and organic linkers, offer three-dimensional compartments that interact with enzymes through H-bonding interactions,^12^ thereby inducing conformational changes and elevating enzyme activity.^13,14^ While MOF-encapsulated enzymes have evolved from H_2_O_2_ degradation^15,16^ to diverse applications like CO_2_ reduction,^17,18^ hydrolysis,^19^ oxidation,^20^ and photocatalysis,^21^ the small pore apertures of existing frameworks limit participation in catalysis involving larger molecules. Therefore, there has been a decade-long desire to design processes for MOFs with large apertures that are both enzyme- and environment-friendly. Extending linker length for isoreticular frameworks^22−28^ offers a solution, but the cost of the multistep synthesis and interpenetration structures limit the use of long linkers for the large-aperture enzyme@MAF catalysts.

Among MOFs, zeolitic imidazolate frameworks (ZIFs) distinguish themselves with commercially available imidazolate linkers. They achieve heterogeneity through mix-linkers, creating over 200 structures and 20 topologies with well-defined spatial arrangements.^29,30^ One notable feature of ZIFs is their ability to generate polymorphic topology structures from the same starting materials by varying reaction conditions and additives. ZIF-8 has served as a benchmark in the enzyme@MAF catalysis field for a decade, owing to its synthesis under enzyme-compatible conditions at room temperature in water.^15,16,31−36^ However, ZIF-8’s largest pore aperture is relatively small (3.4 Å),^37,38^ limiting its practical application in large-molecule synthesis and catalysis. Synthesizing ZIFs with large apertures typically requires high temperatures and organic solvents,^37^ causing significant enzyme deactivation. Thus, the synthesis of larger-aperture ZIFs under enzyme- and ecofriendly conditions is crucial for progress in MOF-encapsulated enzymes.

Unlike the conventional trial-and-error approach typically employed in MOF invention, here we introduce a straightforward and observable process for synthesizing polymorphic ZIFs at room temperature in water—a condition known for its enzyme-friendly nature. By precisely adjusting imidazole linker micelles’ size through linker concentrations, we synthesized seven distinct topological ZIFs in pure phases, including MAF-6 (named after “metal azolate frameworks”^39,40^), featuring an impressive large aperture of 7.6 Å—double that of ZIF-8. This larger aperture facilitates the encapsulation of enzymes for catalyzing reactions involving substantial organic substrates (Figures S1–S2). To demonstrate the potential of MAF-6 as a platform for asymmetric catalysis, we encapsulated *Burkholderia cepacia* lipase (BCL), creating BCL@MAF-6-SDS (SDS = sodium dodecyl sulfate), which outperformed a catalytic efficiency (*K*_cat_/*K*_m_) that was 420 times greater than that of BCL@ZIF-8. Applying this biocatalyst in practical drug precursor synthesis showcases 94–99% enantioselectivity and near-quantitative yields. Our research provides a profound insight into micelles-directed de novo encapsulation of enzymes in ZIFs with large apertures, marking a significant advancement of enzyme@MAF catalysts from proof-of-concept to a groundbreaking achievement in practical asymmetric synthesis of organics and pharmaceuticals.

## Results and Discussion

### Linker Micelles-Directed Synthesis of ZIFs in Water at Room Temperature

Traditional MAF-6 synthesis, common in typical ZIF synthesis, requires bases, ethanol solvent, organic templates, and high temperature risking enzyme deactivation. To meet our goal of an advanced biocatalyst, it is crucial to develop a synthetic approach for MAF-6 that prioritizes enzyme compatibility and environmental sustainability.^41^

**Figure 1:**
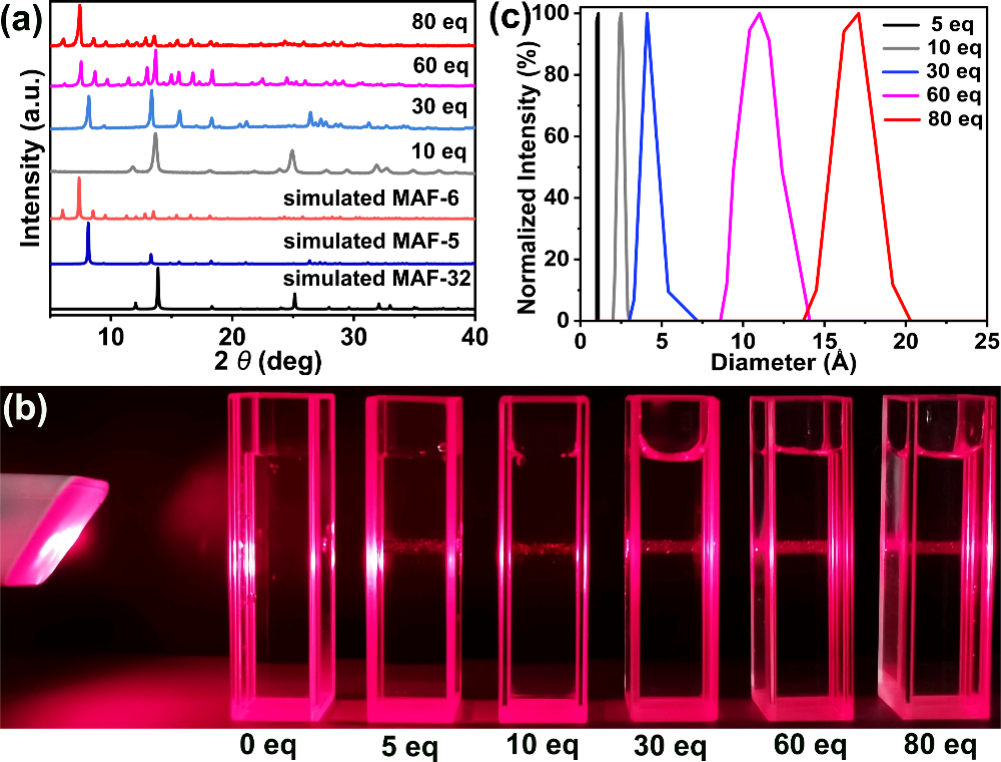
(a) PXRD patterns of MAF-32, MAF-5, and MAF-6 synthesized with different concentrations of HeIM. The default value of 1 equiv (1 equiv) is 0.02 mol L^–1^. (b) The Tyndall effect of HeIM at different concentrations under laser irradiation. (c) The micelle size of HeIM at different concentrations determined by DLS.

In our study, we used water-soluble Zn(NO_3_)_2_ as the Zn^2+^ source. Maintaining a constant Zn^2+^ concentration (0.02 mol L^–1^, 1 equiv) in aqueous solution at room temperature, we varied the 2-ethylimidazole (HeIM) concentration from 5 to 80 equiv relative to Zn^2+^. At 5–20 equiv HeIM, MAF-32 formed solely; at 30 equiv, only MAF-5 formed; and at 40 equiv, pure-phase MAF-6 was exclusively produced (Figure 1 a and Figure S3). Continued increasing HeIM concentration improved MAF-6 crystallinity. Surprisingly, this MAF-6 synthesis requires no additional base or template, as we hypothesize that excess HeIM serves as a linker, base, and, crucially, a self-templating agent in the framework formation.

To explore the self-template effect, various concentrations of HeIM aqueous solution were tested for the Tyndall effect (Figure 1b). The transition from zero to strong scattered light intensity as HeIM concentration increased (0 to 80 equiv) suggests the formation and growth of HeIM micelles in the solution. Dynamic light scattering (DLS) quantitatively assessed micelle size, revealing a single-peak distribution and an increase from 1.0 to 17.0 Å as HeIM concentration went from 5 to 80 equiv (Figure 1c). The micelle size increase corresponds to the progression of aperture and cage sizes in their respective ZIF structures—MAF-32, MAF-5, and, ultimately, MAF-6. (Figure S4).

Furthermore, examining ^1^H NMR spectra with TMS as an external standard revealed a 0.25 ppm upfield shift in HeIM proton chemical shift as its concentration increased from 5 to 80 equiv (Figure S5). This shift indicates changes in HeIM’s chemical environment, suggesting the aggregation of HeIM molecules and weak interactions among them. Diffusion ordered spectroscopy (DOSY)^42^ measured diffusion coefficients, determining the particle hydrodynamic diameter of 2.0 Å at 5 equiv and 10.4 Å at 80 equiv solutions with the Stokes–Einstein equation and the volume formula for spheres^43^ (Figure S6 and Table S1). The DOSY experiment confirmed that higher HeIM concentrations lead to the aggregation of larger-size micelles. The Tyndall effect, along with DLS and DOSY measurements, qualitatively and quantitatively shows that increasing HeIM concentration leads to larger micelle sizes, directing the formation of polymorphic ZIFs with expanded apertures and cages. In our proposed mechanism, HeIM linker aggregation initiates micelle formation, interacting with Zn^2+^ ions to generate ZIF frameworks. Higher HeIM concentration enlarges micelle size, serving as a larger template for ZIF formation. Increasing HeIM from 5 to 80 equiv transforms pure-phase formation from nonporous MAF-32^39^ to elliptical-apertured MAF-5 (7.3 × 5.0/4.1 Å^2^)^44^ and finally to MAF-6 with a larger circular aperture of 7.6 Å.

Exploring micelle-directed ZIF synthesis, we varied concentrations of 2-methylimidazole (2-HmIM) and imidazole (HIM). Increasing 2-HmIM concentration enlarged the micelle diameter from 1.3 to 14.5 Å, forming nonporous ZIF-L^45^ to ZIF-8 with a 3.4 Å aperture (Figures S7–S10). Similar trends were observed with HIM, where higher concentrations transformed micelles from 1.3 to 7.9 Å, resulting in nonporous ZIF-61^26^ to a dense ZIF (CCDC: 745450)^46^ (Figures S11–S14). Elevated 2-HmIM or HIM concentrations also enhanced the Tyndall effect (Figures S8a and S12a).

With a high concentration of HeIM solution (80 equiv), we explored recycling it as a mother liquor. After each synthesis cycle, MAF-6 products were filtered, washed, and dried, with the weight determining HeIM and Zn(NO_3_)_2_ consumption. Replenishing the filtrate with HeIM and Zn(NO_3_)_2_, we repeated this process for 20 cycles. The product maintained a well-defined crystal structure and consistent crystallinity, resulting in a stable final yield of approximately 50% for each cycle (Figure S15). This recycling synthesis demonstrates continuous MAF-6 production using the mother liquor solution, reducing raw material waste in potential industrial applications and aligning with green and environmentally friendly production principles.

### Synthesis and Characterization of BCL@MAF-6

Given the high hydrophobicity of MAF-6 and the hydrophilicity of the BCL enzyme, a higher concentration of amphiphilic HeIM is needed for effective BCL encapsulation during synthesis. Under 80 equiv HeIM conditions, BCL mixed with HeIM and Zn(NO_3_)_2_ formed BCL@MAF-6 composite with excellent crystallinity (Figure S16a). To investigate the surfactant effect on activity, various surfactants (PEG, PVP, SDS, and SDBS) were added as starting materials in a one-pot synthesis to produce BCL@MAF-6-X (X = PEG, PVP, SDS, and SDBS) (Figures S16b and S16c). For comparison, BCL@ZIF-8 and BCL@ZIF-8-SDS were obtained using a similar one-pot method (Figure S16d). All enzyme@MAFs exhibited high crystallinity in their PXRD patterns.

**Figure 2:**
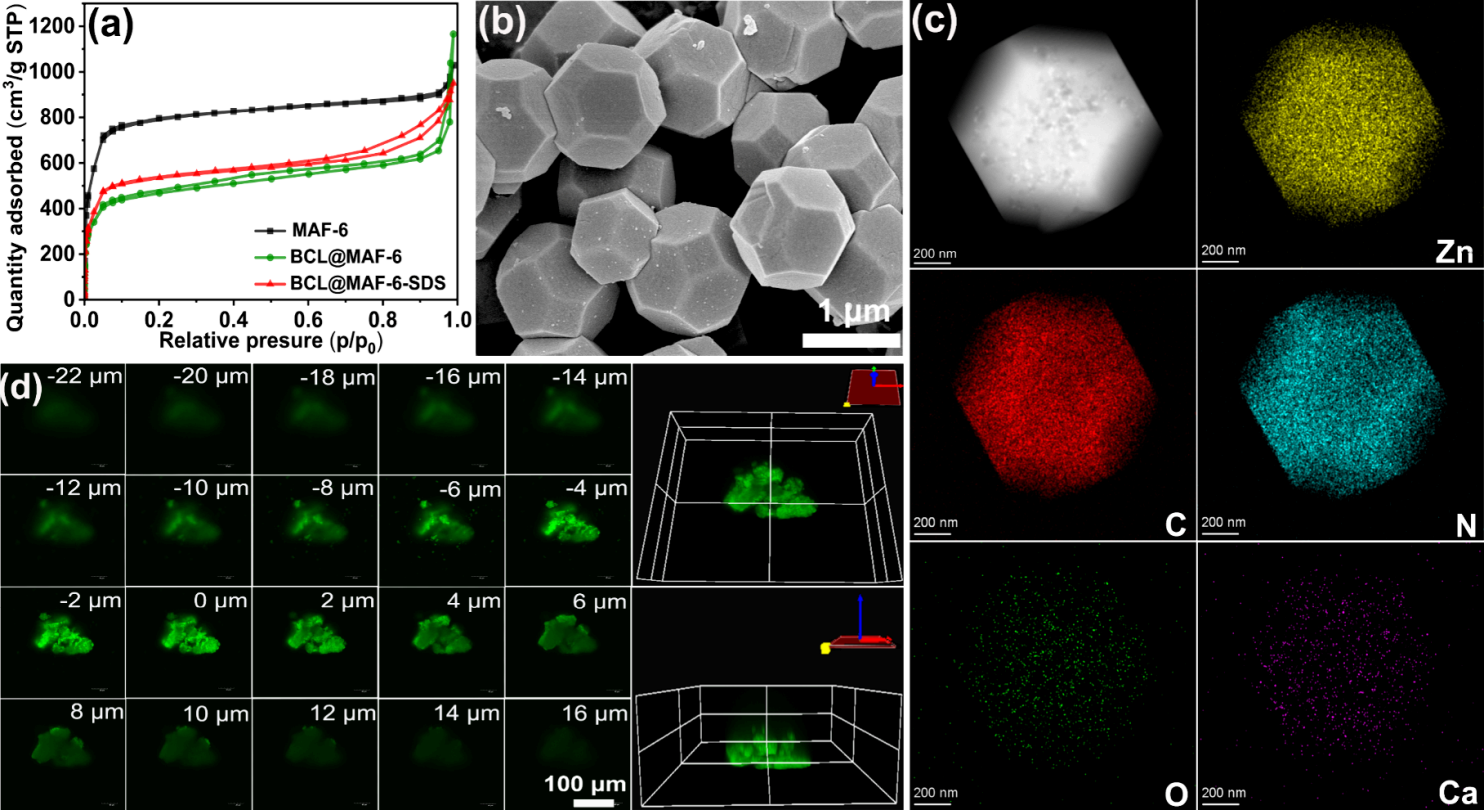
(a) N_2_ adsorption and desorption isotherms of MAF-6, BCL@MAF-6, and BCL@MAF-6-SDS. (b) SEM image of BCL@MAF-6. (c) EDS mapping of element distribution of BCL@MAF-6 for Zn, C, N, O, and Ca by TEM. (d) CLSM images of a series of focal planes of FITC-labeled BCL@MAF-6-SDS measured at 2 μm intervals along the *z*-axis and the FITC-labeled BCL@MAF-6-SDS of CLSM images in 3D view, top view (top), and main view (below).

N_2_ adsorption–desorption results indicate nearly identical specific surface areas for MAF-6 (1809.1 m^2^ g^–1^) and surfactant-modified MAF-6-SDS (1875.1 m^2^ g^–1^), suggesting SDS introduction has no impact on crystal surface area (Figure S17 and Table S2). In contrast, BCL@MAF-6 and BCL@MAF-6-SDS exhibit lower specific surface areas (1219.9 and 1297.9 m^2^ g^–1^, respectively) compared to MAF-6 and MAF-6-SDS, attributed to pore and channel occupation by BCL (Figure 2a). Similarly, BCL@ZIF-8 also displays a lower specific surface area (1134.9 m^2^ g^–1^) compared to ZIF-8 (1573.3 m^2^ g^–1^) due to enzyme encapsulation (Figure S18 and Table S2). Scanning electron microscope (SEM) analysis reveals a chamfered cube structure for BCL@MAF-6 crystals, consistent in size (approximately 1 μm) and structure with pure MAF-6 (Figure 2 b and Figure S19). Energy dispersive spectroscopy (EDS) shows a uniform distribution of C, N, Zn, O, and Ca elements, indicating successful BCL incorporation into MAF-6 crystals (Figure 2c). Similarly, SEM images of ZIF-8 and BCL@ZIF-8 demonstrate intact crystallinity and morphology (Figures S20–S21). To provide further evidence for BCL incorporation into ZIFs, we measured FTIR spectroscopy and used fluorescein isothiocyanate (FITC) to label BCL. A distinctive peak around 1650 cm^–1^ of the internal C=O vibration (amide I band), indicating the presence of BCL,^47^ was observed in the spectra of BCL@MAF-6, BCL@MAF-6-X, and BCL@ZIF-8 compared to original ZIFs (Figure S22). Confocal laser scanning microscopy (CLSM) images showed FITC-labeled BCL uniformly distributed within crystals, confirming its presence inside rather than just on crystal surfaces (Figure S23).^9,48^ The 3D view CLSM image of FITC-BCL@MAF-6-SDS along the *z*-axis scanning further confirmed even enzyme distribution inside the crystals (Figure 2d).^49^ SDS–PAGE analysis on BCL@MAF-6 and surface-adsorbed BCL/MAF-6 showed that, after enzyme denaturation, surface washing, and framework digestion, BCL@MAF-6 displayed a 33 kDa protein band, similar to free BCL (Figure S24). In contrast, no band for BCL/MAF-6 was observed under the same treatment. This confirms effective washing of BCL adsorbed onto the MAF surface, affirming that, in the case of BCL@MAF-6, BCL is genuinely encapsulated inside the MAF-6 crystals rather than merely adsorbed on the surface.^49,50^

### Optimization of Biocatalyst BCL@MAF-6

The optimal BCL loading in MAF-6 was determined by evaluating its activity in the hydrolysis of *p*-nitrophenyl butyrate (NPB). BCL@MAF-6 activity initially increased and then decreased with BCL loading. The highest activity, reaching 36.5% of free BCL activity, was observed at a loading of 0.18 mg mg^–1^ (Table S3 and Figure S25). Control experiments showed that BCL incubated with Zn(NO_3_)_2_ or HeIM retained 91.5% and 93.6% of free enzyme activity, suggesting the BCL@MAF-6 synthesis is enzyme-friendly.

Recent reports show that surfactants can influence the enzyme activity inside MOFs.^51^ We found the cationic surfactants (CTAB and DTAB) inhibited MAF-6 crystal nuclei formation during BCL@MAF-6 synthesis, likely due to their accumulation on HeIM micelle surfaces obstructing MAF-6 formation.^52^ Successful synthesis of BCL@MAF-6-X (X = surfactant) was achieved with nonionic (PEG and PVP) and anionic (SDS and SDBS) surfactants. Anionic surfactant-regulated BCL@MAF-6-SDS (73.8% of free enzyme activity) and BCL@MAF-6-SDBS (66.1%) exhibited nearly double the activity of BCL@MAF-6 (36.5%). In contrast, nonionic surfactant-regulated BCL@MAF-6-PEG and BCL@MAF-6-PVP displayed similar activities to BCL@MAF-6, with values of 34.3% and 33.6%, respectively (Figures S26–S27). For a sharp comparison, MAF-6 and MAF-6-SDS were employed to adsorb the same amount of BCL on their surfaces. Interestingly, BCL/MAF-6-SDS exhibited similar activity (26.7%) to that of BCL/MAF-6 (25.8%). These results highlight the positive impact of anionic surfactants in enhancing BCL@MAF-6 activity, while the effect of nonionic surfactants is minimal. Moreover, BCL@ZIF-8 (5.8%) and BCL@ZIF-8-SDS (7.2%) activities were significantly lower than those of BCL@MAF-6 (36.5%) and BCL@MAF-6-SDS (73.8%), suggesting that MAF-6’s larger aperture and pore size provide better accessibility for substrate and product molecules. The alkyl vibration peak in SDS is around 2900 cm^–1^ in the FTIR spectra (see Figure S22). This suggests the presence of SDS in the SDS-regulated MAF-6 framework. To measure the SDS content in the BCL@MAF-6-SDS sample, ^1^H NMR spectra were employed, unveiling a molar ratio of 8% relative to HeIM and a concentration ratio of 0.8:1 to BCL (Figure S28).

**Figure 3:**
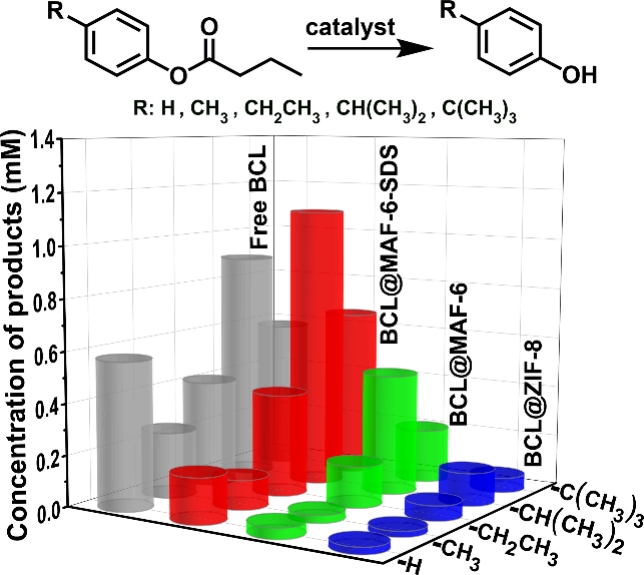
Catalytic hydrolysis of a series of phenolic esters with free BCL, BCL@MAF-6-SDS, BCL@MAF-6, and BCL@ZIF-8 catalysts.

To explore the influence of MAF-6’s hydrophobic nature for catalysis, phenolic ester hydrolysis reactions with various hydrophobic substituents (H, CH_3_, C_2_H_5_, CH(CH_3_)_2_, and C(CH_3_)_3_) at the 4-position of the phenol group were conducted (Figure 3 and Figure S29). BCL@MAF-6-SDS and free BCL exhibited higher activities than BCL@MAF-6 and BCL@ZIF-8 for all substrates. Notably, BCL@MAF-6-SDS demonstrated 1.2 times the activity of free BCL when the substituent was CH(CH_3_)_2_. As substrate hydrophobicity increased, transitioning from CH_3_ to CH(CH_3_)_2_, BCL@MAF-6-SDS showed a significant 9.2-fold increase in conversion compared to a more modest 3.5-fold increase for free BCL (Tables S4–S5). This indicates that MAF-6’s hydrophobic framework enhances enzyme catalytic performance for hydrophobic substrates that are commonly encountered in organics and drug molecules. Encapsulation in different topological structures (MAF-32 or MAF-5) revealed that BCL@MAF-32 exhibited minimal activity due to its compact structure, while BCL@MAF-5 and BCL@MAF-6 displayed similar activities (Figures S30–S31).

Expanding biomolecule encapsulation in MAF-6, we successfully encapsulated cytochrome c (Cyt c) and glucose oxidase (GOx) in MAF-6 (Figure S32). Cyt c@MAF-6-SDS and GOx@MAF-6-SDS composites exhibited excellent activity, surpassing free Cyt c and GOx by 144% and 236%, respectively (Figures S33–S34). Our findings highlight the hydrophobic nature of MAF-6 and the surfactant effect, broadening enzyme immobilization techniques with improved activity and enhancing MAF-6’s versatility for various enzyme encapsulation applications.

### Asymmetric Catalysis with BCL@MAF-6-SDS

Leveraging the enhanced catalytic activity of BCL@MAF-6-SDS due to the framework’s large aperture and hydrophobic nature, we applied this composite to enantioselective asymmetric catalysis. Employing the kinetic resolution of 4-phenyl-3-buten-2-ol, where BCL selectively reacts with the *R* enantiomer, resulting in a theoretical yield of 50% from a racemic mixture, we optimized conditions in various solvents and temperatures (Table 1). BCL@MAF-6-SDS outperformed in nonpolar solvents, especially *n*-hexane, achieving the highest conversion at 45 °C. Under optimized conditions, BCL@MAF-6-SDS exhibited a significantly higher reaction yield (39.8%) compared to BCL@MAF-6 (5.7%), BCL@ZIF-8 (0.2%), and free BCL (35.9%) (entries 1, 12–14).

**Table 1:**
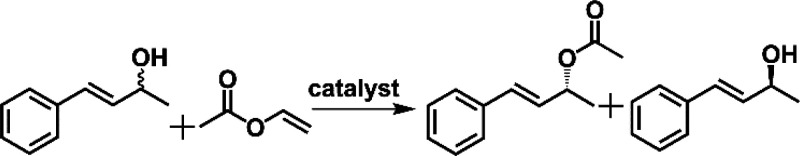
Kinetic Resolution of Secondary Alcohols with Vinyl Acetate^a^

entry	catalyst	*T* (°C)	solvent	yield (%)^b^
1	BCL@MAF-6-SDS	45	*n*-hexane	39.8
2	BCL@MAF-6-SDS	45	toluene	9.9
3	BCL@MAF-6-SDS	45	DCE	6.7
4	BCL@MAF-6-SDS	45	DCM	3.4
5	BCL@MAF-6-SDS	45	THF	trace
6	BCL@MAF-6-SDS	45	acetone	trace
7	BCL@MAF-6-SDS	45	MeCN	trace
8	BCL@MAF-6-SDS	15	*n*-hexane	9.8
9	BCL@MAF-6-SDS	25	*n*-hexane	19.6
10	BCL@MAF-6-SDS	35	*n*-hexane	26.2
11	BCL@MAF-6-SDS	55	*n*-hexane	32.9
12	BCL@MAF-6	45	*n*-hexane	5.7
13	BCL@ZIF-8	45	*n*-hexane	0.2
14	free BCL	45	*n*-hexane	35.9

a Reaction conditions: 4-phenyl-3-buten-2-ol (0.3 mmol), vinyl acetate (0.75 mmol), catalyst (2 mg, based on BCL), solvent (2.5 mL) for 12 h.

b Calculated by ^1^H NMR.

**Figure 4:**
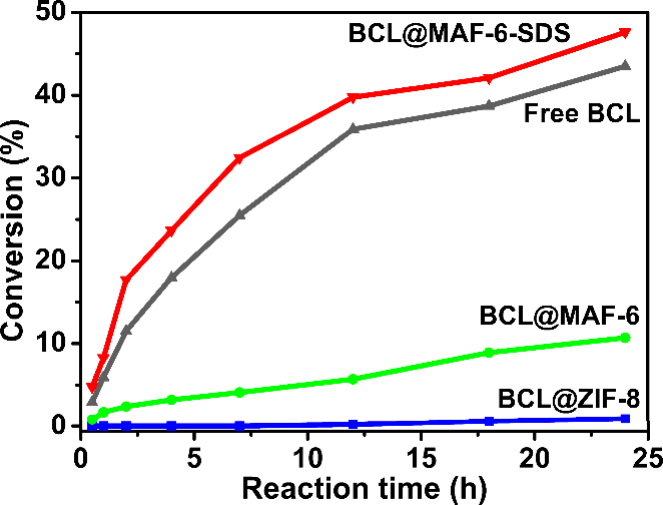
Kinetic resolution of 4-phenyl-3-buten-2-ol in *n*-hexane at 45 °C in different times with free BCL, BCL@ZIF-8, BCL@MAF-6, and BCL@MAF-6-SDS catalysts.

Detailed kinetics investigation (Figure 4 and Figure S35) revealed a direct correlation between catalytic rate and activity: BCL@MAF-6-SDS > BCL ≫ BCL@MAF-6 > BCL@ZIF-8. After 24 h, BCL@MAF-6 reached a yield of 10.7%, nearly 12 times higher than BCL@ZIF-8 (0.9%). Free BCL and BCL@MAF-6-SDS achieved yields of 43.5% and 47.6%, respectively, approaching the theoretical quantitative yield (50%) for BCL kinetic resolution. Importantly, BCL@MAF-6-SDS exhibited excellent enantioselectivity, with the product over 99% *ee* (*R* enantiomer) (Figure S36). These findings indicate that BCL encapsulation and SDS introduction did not impact the enzyme’s stereoselectivity within MAF-6.

Analyzing initial reaction rates with varying substrate concentrations, we applied the Lineweaver–Burk equation for free BCL, BCL@MAF-6, BCL@MAF-6-SDS, and BCL@ZIF-8 (Figure S37 and Table S6).^7,20^ All four composites followed Michaelis–Menten kinetics, with BCL@MAF-6 displaying a *V*_max_ approximately 29 times higher than BCL@ZIF-8, indicating superior reaction rates. BCL@MAF-6-SDS exhibited a slightly higher *V*_max_ value (0.14 mM min^–1^) than free BCL (0.12 mM min^–1^), seven times higher than BCL@MAF-6, suggesting improved reaction rates with SDS. Comparing the Michaelis constant (*K*_m_), BCL@MAF-6 and BCL@MAF-6-SDS displayed lower values than free BCL and BCL@ZIF-8, indicating robust substrate affinity. The catalytic efficiency parameter *K*_cat_/*K*_m_ for BCL@MAF-6 and BCL@MAF-6-SDS demonstrated efficiencies 112 and 420 times higher, respectively, compared to BCL@ZIF-8, emphasizing MAF-6’s preference for enzyme immobilization due to its larger aperture.

In industrial settings, catalyst reuse and recycle are pivotal. The BCL@MAF-6 catalyst underwent a six-cycle test, displaying no significant activity decrease, maintaining a relative yield above 80% throughout (Figure S38a). The catalyst’s crystallinity largely remained unchanged, indicating excellent recyclability and reusability (Figure S38b). The SEM image still maintained good morphology after six cycles of BCL@MAF-6 catalysis (Figure S39). These findings highlight the practical viability of BCL@MAF-6 as an industrial catalyst.

### Catalytic Synthesis of Chiral Drug Precursors Molecules

**Figure 5:**
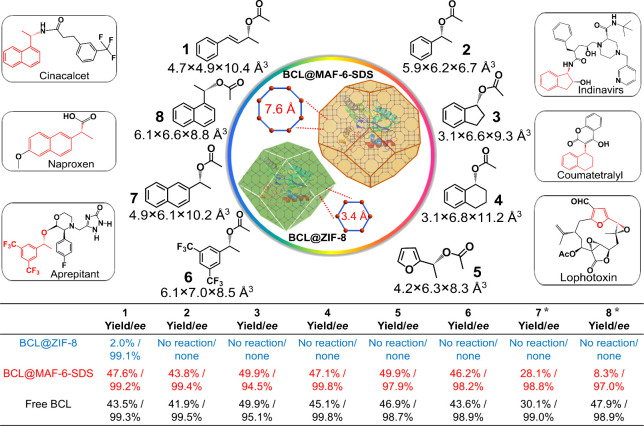
Catalytic synthesis of chiral drug precursors with BCL@MAF-6-SDS, free BCL, and BCL@ZIF-8 catalysts. ^*^The enzyme CAL-B was employed in place of BCL for **7** and **8**.

Asymmetric catalysis has gained significant traction in the pharmaceutical industry, playing a crucial role in the enantioselective synthesis of chiral target molecules throughout various stages of drug discovery and development. Based on the significantly larger aperture of MAF-6 compared to that of ZIF-8, BCL@MAF-6-SDS exhibits the ability to access larger organic substrates in organic synthesis. Based on this, we extended the application of the kinetic resolution to the synthesis of six chiral drug precursor molecules, which exceeded the pore size of ZIF-8 (Figure 5 and Table S7). The corresponding drug molecules are shown near their precursors. Initially, the synthesis of a smaller molecule **1** was tested, and the yields of BCL@ZIF-8, free BCL, and BCL@MAF-6-SDS after 24 h were 2.0%, 43.5%, and 47.6%, respectively, indicating the beneficial role of the larger aperture of MAF-6 in substrate accessibility. As the size of the substrates increased, the catalytic reaction using BCL@ZIF-8 failed to produce detectable products, while both free BCL and BCL@MAF-6-SDS achieved satisfactory yields. For products **1**–**6**, BCL@MAF-6-SDS demonstrated superior catalytic performance with 43.8–49.9% yield and 94.5–99% *ee*. To investigate the universality of other enzymes encapsulated in MAF-6, lipase CAL-B was introduced into MAF-6 (Figure S40), and its application in asymmetric catalysis was explored. As shown in Figure 5, the size of product **7** (4.9 × 6.1 × 10.2 Å^3^) was slightly smaller than the aperture of MAF-6 (7.6 Å), resulting in 28.1% yield with 98% *ee*. The yield was slightly lower than that of free CAL-B (30.1%). On the contrary, when addressing a more sizable product **8** (6.1 × 6.6 × 8.8 Å^3^), which is similar to the aperture of MAF-6, it resulted in a notably lower yield in comparison to free CAL-B.

Our application in synthesizing drug precursors demonstrates that MAF-6 with its larger aperture is superior to ZIF-8 in chiral substrate screening without compromising the enzymes’ stereoselectivity. Accordingly, MAF-6 establishes its potential as a new benchmark for the application of MOFs in immobilizing enzymes and other material.

### Thermal and Chemical Stability

Free enzymes are susceptible to lose their activity under extreme conditions such as high temperatures and organic solvents.^8^ However, when enzymes are encapsulated in MOFs, they can maintain a certain level of activity under the protective effect of the framework. In light of this, we conducted stability tests on BCL@MAF-6 in extreme environments (Figure S41). First, thermal stability was investigated. After incubation in a 70 °C aqueous solution for 0.5 and 1 h, free BCL only retained 55% and 25% of its original activity, respectively. In contrast, BCL@MAF-6 retained 68% and 64% of its original activity under the same conditions. Moreover, when incubated at higher temperatures (80 and 90 °C) for 0.5 h, BCL@MAF-6 maintained better activity (63% and 58%, respectively) compared to free BCL (17% and 14%, respectively). Considering the solvent environment in which enzymes catalyze reactions, we also tested the compatibility of free BCL and BCL@MAF-6 with organic solvents. Encouragingly, BCL@MAF-6 retained 93%, 89%, 97%, and 92% of its original activity after incubation in MeOH, EtOH, DMF, and DMSO, respectively, whereas the activity of free BCL in these solvents decreased significantly to 4–14%. Overall, these tests exhibited that the stability of BCL in extreme environments was greatly enhanced inside MAF-6.

## Conclusions

Designing crystalline frameworks with large apertures for catalysis poses a challenge. While the isoreticular principle has worked for many MOFs, adapting it to ZIFs is rarely achievable due to the requirement of ∼145° M–Im–M angle in the expansion of imidazolate linkers. After years of trial-and-error and high-throughput methods,^29,30^ researchers have defined three design principles for creating extra-large apertures and cages in ZIFs.^30,37^ These principles, based on a molecular-level understanding of imidazole linker steric index and link–link interaction, particularly steric repulsion in mixed-linker systems, offer insights into known ZIFs’ formation and predict sizes for new ZIF structures. Building upon these principles, our micelle-induced approach takes advantage of attractive interactions among identical linkers. Through controlled linker concentration, we can generate three-dimensional micelles of desired sizes, leading to the formation of pure-phase polymorphic ZIFs with corresponding sizes of cages and apertures. The presence of micelles can be observed through the Tyndall effect, and their size can be measured using DLS analysis prior to initiating the ZIF synthesis. Our strategy not only provides a direct visible three-dimensional guidance for ZIF design and synthesis using tunable experimental parameters but also extends the green synthesis conditions of aqueous solution and room temperature for not only SOD ZIF formation but also six additional ZIF topologies. Among them, MAF-6 stands out with the largest cage and aperture. By encapsulating BCL, MAF-6 exhibits a 112-fold increase in reactivity compared to ZIF-8, the benchmark in the enzyme@MAF field, particularly in the rate parameter *K*_cat_/*K*_m_. The enhanced reactivity of BCL@MAF-6 is attributed to MAF-6’s eight-membered ring aperture, which is more than twice the size of ZIF-8’s largest aperture. This larger aperture facilitates easy diffusion of substrates to the encapsulated enzyme. The biocatalyst was further developed by incorporating surfactant-modified frameworks, leading to an enhanced substrate enrichment effect and a more open-lid enzyme conformation. The catalytic efficiency *K*_cat_/*K*_m_ of BCL@MAF-6-SDS exhibited a remarkable improvement, approximately 420 times greater than that of BCL@ZIF-8. Taking these advantages, we have effectively broadened the scope of enzyme@MAF catalysis, progressing from small-molecule reactions to practical applications in chiral organic molecule synthesis. Notably, the system demonstrated high enantioselectivity and nearly quantitative conversion in the asymmetric synthesis of drug precursors, an accomplishment that has been sought after in the MOF catalysis field for a decade.

Our research not only successfully expanded enzyme@MAF catalysis from a proof-of-concept to practical applications in chiral organic molecule synthesis but also spanned the full development stream, from designing MOF frameworks to catalyzing drug precursors synthesis. This holistic approach breaks down barriers between MOF invention and catalysis, providing valuable guidance for researchers in both realms.

## Supporting Information Available

Detailed description of synthesis, catalysis, and materials characterization including XRD, DLS, IR, CLSM, SEM, gas adsorption, ^1^H and ^13^C NMR spectra, HPLC, etc. (PDF)
Transparent Peer Review report available (PDF)
