# Supplementary material for: Highly Enantioselective Catalysis by Enzyme Encapsulated in Metal Azolate Frameworks with Micelle-Controlled Pore Sizes
Source: ACS Cent Sci. 2024 Jan 18;10(2):358–66. doi: 10.1021/acscentsci.3c01432 (PMC10906037; doi:10.1021/acscentsci.3c01432)
Supplement: Supplementary file 1 — oc3c01432_si_001.pdf [file oc3c01432_si_001.pdf]

Supporting Information (73 pages)

Highly Enantioselective Catalysis by Enzyme Encapsulated in Metal Azolate Frameworks with Micelle-Controlled Pore Sizes

Hao Ren,[1] Jian Yuan,[3,*] Yi-Ming Li,[1,4] Wen-Jing Li,[1] Yi-Hang Guo,[5,6] Yi-Bo Zhang,[6] Bing-Hao Wang,[1] Kaili Ma,[7] Lu Peng,[7] Guping Hu,[8] Wen-Qi Wang,[2] Hailong He,[2] Lien-Yang Chou,[2] Ming-Hua Zeng,[6] Yue-Biao Zhang,[2,*] and Lin Cheng[1,*]

[1]*Jiangsu Engineering Laboratory of Smart Carbon-Rich Materials and Device, School of Chemistry and Chemical Engineering, Southeast University, Nanjing 211189, China.*

[2]*School of Physical Science and Technology, Shanghai Key Laboratory of High-Resolution Electron Microscopy, State Key Laboratory of Advanced Medical Materials and Devices, ShanghaiTech University, Shanghai 201210, China.*

[3]*Avogadral Solutions, 3130 Grants Lake Blvd #18641, Sugar Land, Texas 77496, United States.*

[4]*School of Chemistry and Chemical Engineering, Anhui University, Hefei 230601, China.*

[5]*State Key Laboratory of Structural Chemistry, Fujian Institute of Research on the Structure of Matter, Chinese Academy of Sciences, Fujian 350002, China.*

[6]*School of Chemistry and Pharmaceutical Sciences, State Key Laboratory for Chemistry and Molecular Engineering of Medicinal Resources, Guangxi Normal University, Guilin 541004, China.*

[7]*Analysis and Testing Center, Southeast University, Nanjing 211189, China.*

[8]*School of Chemistry, Sun Yat-Sen University, Guangzhou 510275, China.*

[*]To whom correspondence should be addressed:

lcheng@seu.edu.cn (C.L.) or zhangyb@shanghaitech.edu.cn (Y.-B.Z.) or jianyuanchem@gmail.com (J.Y.)

Table of Content

Section 1. Methods.. 3

Section 2. Synthesis of Samples 5

Section 3. Linker Micelles-Directed Synthesis of ZIFs 9

Section 4. Synthesis and Characterization of BCL@MAF-6 16

Section 5. Optimization of Biocatalyst BCL@MAF-6. 22

Section 6. Asymmetric Catalysis with BCL@MAF-6-SDS 32

Section 7. Catalytic Synthesis of Chiral Drug Precursor Molecules................................. 36

Section 8. Thermal and Chemical Stability... 38

Section 9. NMR Spectroscopy 39

Section 10. HPLC Traces.. 55

Section 11. References.. 72

Section 1. Methods

Chemicals. Lipase BCL from Burkholderia cepacia, CAL-B (Novozym 435 from Candida Antarctica), Cytochrome c (Cyt c) and Glucose oxidase (GOx) were purchased from Sigma–Aldrich. BCA Protein Quantitation Assay was purchased from Shanghai yuanye Bio–Technology Co., Ltd. 4-phenyl-3-buten-2-ol, 1-(furan-2-yl)ethan-1-ol and 1-(3,5-bis(trifluoromethyl)phenyl)ethan-1-ol were converted from corresponding ketone (4-phenyl-3-buten-2-one, 2-acetylfuran and 1-(3,5-bis(trifluoromethyl)acetophenone)) using $NaBH_4$ as the reductant.[1] Phenyl ester (phenyl butyrate, *p*-tolyl butyrate, 4-ethylphenyl butyrate, 4-isopropylphenyl butyrate and 4-(tert-butyl)phenyl butyrate) was synthesized according to the previously reported literature (See Section 9.1 NMR spectra of substrates for detailed data.).[2] Other reagents and solvents were purchased from commercials without further purification.

Analytical techniques. Powder X-ray diffraction (PXRD) was the Ultima IV X-ray Diffractometer using Cu Kα radiation under 40 kV and 40 mA.

The Tyndall effect is achieved by illuminating the sample solution with a laser pointer in a dark environment. Dynamic light scattering (DLS) was tested using a Brookhaven Instruments NanoBrook series instrument at 25 °C. Care was taken that the water was pure distilled water and that the inner and outer surfaces of the quartz cuvette were wiped clean.

Scanning electron microscopy (SEM, Hitachi SU8010), transmission electron microscope (TEM, Talos F200X) and energy dispersive X-ray spectrometer (EDX) system were employed to characterize the morphologies, microstructures and the chemical compositions of the products.

N_2 adsorption-desorption (77K) isotherms were performed on a Micromeritics ASAP 2460 (Mike) to determine the BET surface and pore size distribution. Prior to the BET test, solvent exchange (methanol) was carried out by Soxhlet extractor, and then vacuum activation was carried out at 120 °C for 12 hours to remove guest molecules from the pores.

Infrared spectra were measured in the range from 4000 to 400 cm $^{-1}$ using a Nicolet iS10 FTIR spectrometer. Confocal laser scanning microscope (CLSM) with excitation wavelength λ_{ex}= 488 nm and long pass emission wavelength λ_{ex}= 525 nm were obtained on Olympus Fluo View FV3000

confocal microscope.

The enzyme content and enzymatic hydrolysis activity were measured by a Multiskan SkyHigh full wavelength enzyme standard (Thermo Fisher Scientific).

^{1}H and ^{13}C NMR spectra and diffusion ordered spectroscopy (DOSY) were recorded with a Bruker 400 MHz spectrometer (AVANCE III HD 400MHz). The TopSpin controlled NMR data were collected for DOSY diffusion experiments, and the 1- and 2-dimensional spectra were integrated.

Chiral high performance liquid chromatography (HPLC) analysis for *ee* value determinations was performed using a SHIMadzu LC-20AT apparatus equipped with SPD-20 a UV detector and Daicel columns OD–H, OJ–H or AD–H columns, using hexane/iso-propanol as the eluent.

Section 2. Synthesis of Samples

Synthesis of MAF-32, MAF-5 and MAF-6 under different conditions and all experiments were performed at room temperature.

$Zn(NO_3)_2$•$6H_2O$ (148.7 mg, 0.5 mmol) was dissolved in 12.5 mL water to obtain a concentration of 0.04 mol L^{-1} (2 eq). This solution was quickly poured into 2-ethylimidazole (HeIM) aqueous solution (12.5 mL) of the corresponding concentration. The final concentration of Zn^{2+} in the mixture was 0.02 mol L^{-1} (1 eq) and the final HeIM concentration was various from 5 eq to 80 eq. The mixture was stirred vigorously at room temperature for 30 minutes. The solid product was collected by centrifugation and washed 6 times with 20 mL water each time. The final product was freeze-dried before application. Note: Occasional difficulties may arise in the synthesis of MAF-6, possibly attributable to significant fluctuations in room temperature or high humidity.

When the surfactants (Polyethylene glycol (PEG), polyvinyl pyrrolidone (PVP), sodium dodecyl sulfate (SDS) and sodium dodecyl benzene sulfonate (SDBS)) were used to prepare surfactant modified ZIFs, the procedure was similar to the above process, expect that the surfactant (50 mg) was dissolvent in HeIM solution obtain the surfactant concentration of 2 mg mL^{-1} before addition of Zn^{2+} solution.

BCL@MAF-6-X (X = polyvinyl pyrrolidone (PVP), Polyethylene glycol (PEG), sodium dodecyl sulfate (SDS) and sodium dodecyl benzene sulfonate (SDBS)). $Zn(NO_3)_2$•$6H_2O$ (148.7 mg, 0.5 mmol) was dissolved in 12.5 mL water (solution A, 0.04 mol L^{-1}, 2 eq). Various amounts of BCL (0.5 to 3 mg mL^{-1}) were added to 12.5 mL of aqueous solution containing the corresponding surfactant (2 mg mL^{-1}), and HeIM (3.845 g, 40 mmol) was added to the above mixed solution (solution B). Solution A was quickly poured into solution B and stirred for 3 h. It should be noted that when the surfactant is an anionic surfactant, the stirring time is 1.5 hours; the concentration of surfactant should not be lower than 2 mg mL^{-1}, otherwise it will be unfavorable to the formation of MAF-6. Finally, the as-synthesized powder was collected by centrifugation, washed 6 times with 20 mL ultrapure water each time, and freeze-dried.

When varying amounts of BCL were introduced, with SDS as the surfactant, the designations for these samples were as follows: BCL@MAF-6-SDS-0.5, BCL@MAF-6-SDS-1,

BCL@MAF-6-SDS-1.5, BCL@MAF-6-SDS (control), BCL@MAF-6-SDS-2.5, and BCL@MAF-6-SDS-3.

In order to compare with other surfactants, the same method was adopted, and the product name was marked as BCL@MAF-6-PVP, BCL@MAF-6-PEG, BCL@MAF-6-SDBS. BCL@MAF-6 was synthesized using the same method, except that no surfactant was added, and the product name was marked as BCL@MAF-6-0.5, BCL@MAF-6-1, BCL@MAF-6-1.5, BCL@MAF-6, BCL@MAF-6-2.5 and BCL@MAF-6-3.

Synthesis of ZIF-8. $Zn(NO_3)_2{\bullet}6H_2O$ (237.6 mg, 0.8 mmol) was dissolved in 10 mL water to obtain a concentration of 0.08 mol L^{-1}. This solution was added to 2-methylimidazole (2-HmIM) aqueous solution (1.2 M, 10 mL), and continuous stirring for 3 h. The solid product was collected by centrifugation and washed 6 times with 20 mL water each time. The final product was freeze-dried before application.

BCL@ZIF-8. $Zn(NO_3)_2{\bullet}6H_2O$ (237.6 mg, 0.8 mmol) was dissolved in 10 mL water to obtain a concentration of 0.08 mol L^{-1}. This solution was added to the solution containing 2-HmIM (1.2 M, 5 mL) and BCL (2, 2.5, 3 mg mL^{-1}, 5 mL). Then, the mixed solution was continuously stirred for 3 h. Through centrifugation, the product was washed 6 times with 20 mL ultrapure water each time, and then freeze-dried. When varying amounts of BCL were introduced, BCL@ZIF-8-X was flagged as BCL@ZIF-8-2, BCL@ZIF-8, BCL@ZIF-8-3.

BCL@ZIF-8-SDS. The synthesis process is similar to that of BCL@ZIF-8. SDS was added to BCL solution followed by HmIM solution. When varying amounts of BCL were introduced, BCL@ZIF-8-SDS-X was flagged as BCL@ZIF-8-SDS-2, BCL@ZIF-8-SDS, BCL@ZIF-8-SDS-3.

Repeated cycle experiment for MAF-6 synthesis. $Zn(NO_3)_2{\bullet}6H_2O$ (148.7 mg, 0.5 mmol) was dissolved in 12.5 mL water to obtain a concentration of 0.04 mol L^{-1} (2 eq). This solution was added to HeIM (3.845 g, 40 mmol) aqueous solution (12.5 mL). The mixture was stirred for 30 minutes. The MAF-6 products were filtered, washed and dried. The weight of the dried product was measured to determine the consumption of HeIM and zinc nitrate. To replenish the filtrate, appropriate amounts of zinc nitrate and HeIM were added, and this process was repeated for a total of 20 cycles.

For FITC marked FITC-BCL@MAF-6, FITC-BCL@MAF-6-SDS, FITC-BCL@ZIF-8 and

FITC-BCL was used instead of free BCL. Weigh 400 mg of BCL and fluorescein isothiocyanate (FITC, 8 mg) into carbonate–bicarbonate buffered aqueous solution (CBS, 0.1 mmol, 20 mL, pH=9.16) and stir continuously for 3 hours under dark conditions. Free FITC was removed by Sephadex G-25 column under UV-Vis spectrum, and the crude product (FITC-BCL) was centrifugally concentrated by using an ultrafiltration centrifuge tube (Millipore, 15 mL/10 K, 4000 rpm for 25 min). Then the concentrated solution was further washed with ultrapure water, and then concentrated again. This process was repeated three times to completely remove the free FITC and buffer solution, and finally the concentrated solution was freeze-dried for use.

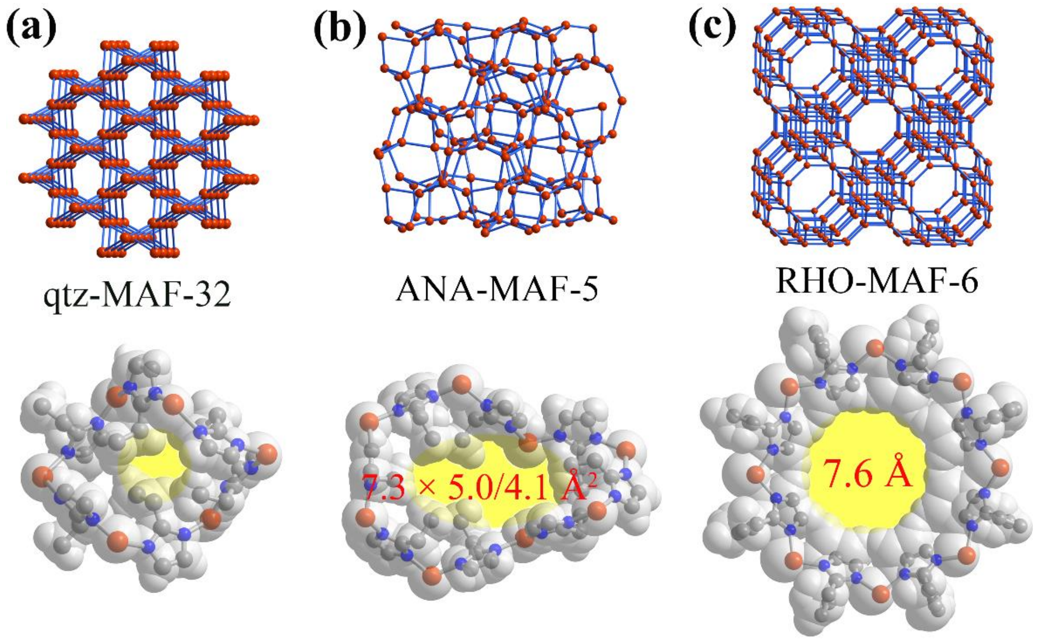

Figure S1. Comparison with the topology structure and windows of MAFs. (a) qtz nonporous topological structure of MAF-32 and nonporous windows. (b) ANA topological structure of MAF-5 and elliptical windows. (c) RHO topological structure of MAF-6 and circular windows.

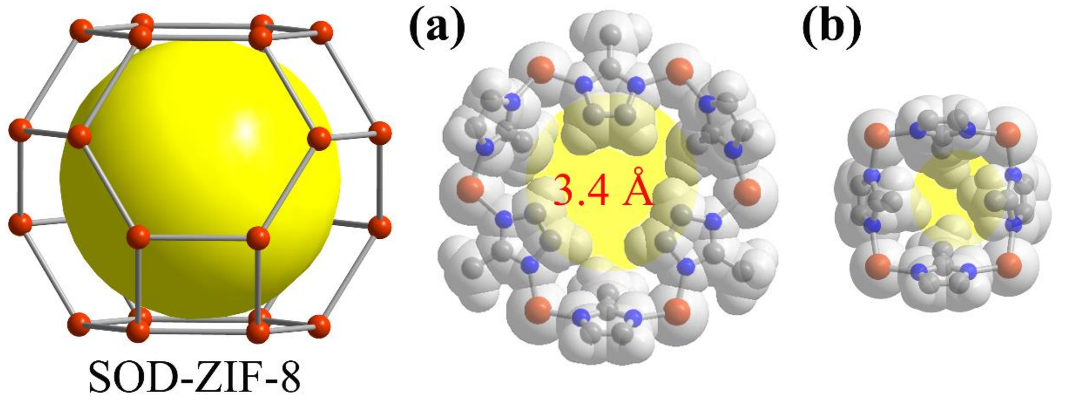

Figure S2. Two kinds of windows for ZIF-8 with SOD topological structure, and the yellow sphere represents the free volume within the framework. (a) 6-membered ring aperture of ZIF-8. (b) 4-membered ring aperture of ZIF-8.

Section 3. Linker Micelles-Directed Synthesis of ZIFs

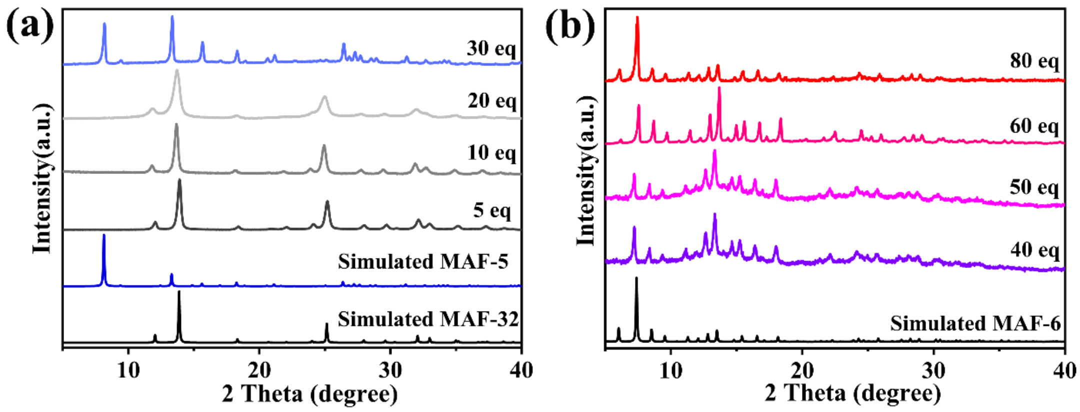

Figure S3. PXRD patterns of MAF-32, MAF-5 and MAF-6 synthesized with different concentrations of HeIM (1 eq = 0.02 mol L^{-1}, the concentration of Zn^{2+} is constantly 1 eq). (a) MAF-32 was formed from 5 eq to 20 eq of HeIM. MAF-5 was formed at 30 eq. (b) MAF-6 was formed from 40 eq to 80 eq.

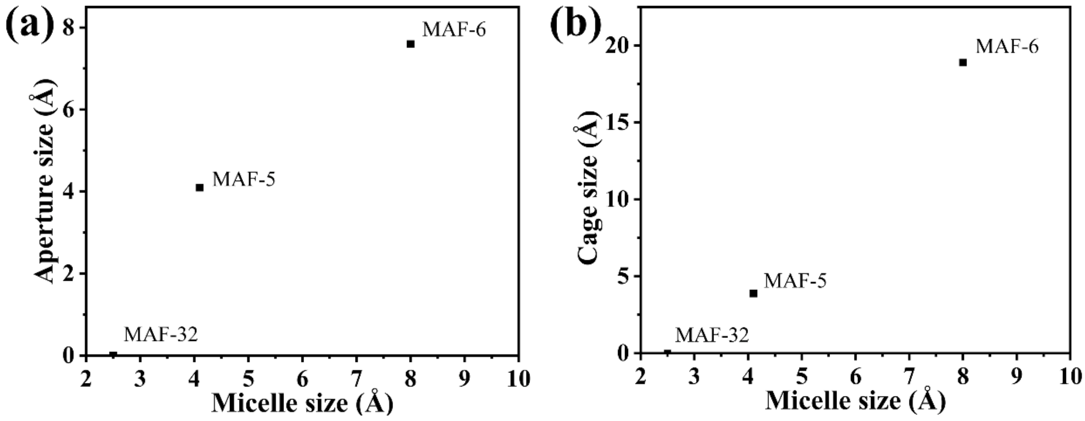

Figure S4. Relation of micelle size to aperture and cage size. (a) The relationship between micelle size and aperture size. (b) The relationship between micelle size and ZIF cage size.

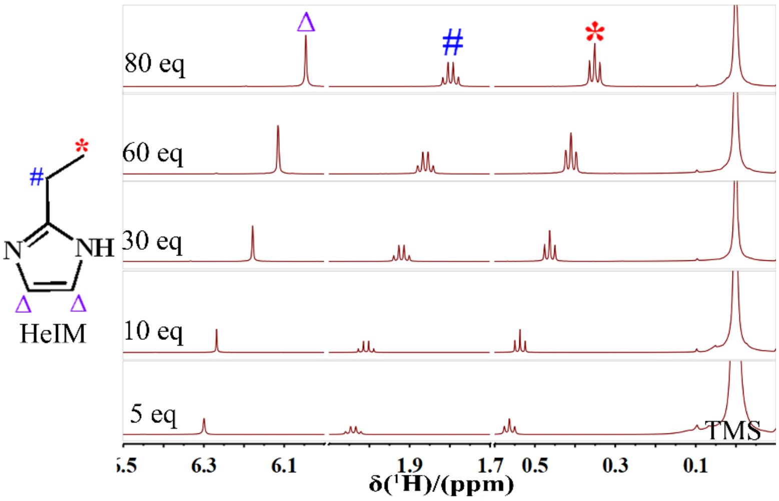

Figure S5. ^{1}H NMR spectra of HeIM at different concentration from 5 eq to 80 eq (1 eq = 0.02 mol L^{-1}). TMS was sealed in glass tube and put inside the HeIM NMR solution as an external standard during the NMR running.

DOSY NMR Experiments.

5 eq and 80 eq of HeIM NMR solution (1 eq = 0.02 mol L^{-1}) were prepared with deuterated water for ^{1}H NMR Diffusion Ordered Spectroscopy (DOSY) analysis. Chromatographic grade methanol was sealed in a glass tube and put inside the HeIM NMR solution as an external standard during the NMR running. The TopSpin controlled NMR data were collected for DOSY diffusion experiments, and the 1- and 2-dimensional spectra were integrated. The self-diffusion coefficient (D) is measured by the DOSY, and the hydrodynamic particle radius (R_H) is calculated using Einstein–Stokes equation ($D = \frac{K_B T}{6\pi\eta R_H}$).[3,4]

The calculation shows the micelles diameter is 2.0 Å for 5 eq HeIM solution and 10.4 Å for 80 eq HeIM solution.

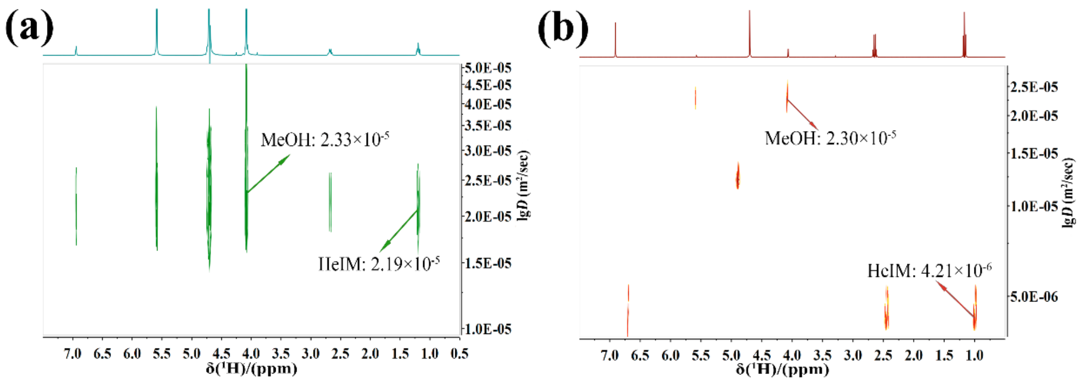

Figure S6. DOSY spectrum analysis. ^{1}H DOSY spectrum of HeIM for (a) 5 eq and (b) 80 eq. (1 eq = 0.02 mol L^{-1}).

Table S1. Calculation of micelle radius and diameter from DOSY NMR data and Einstein–Stokes equation.[3,4]

HeIM	D (m^2 s^{-1})	Micelle Radius R_H(Å)	Micelle Diameter (Å)
**5 eq**	2.19×10^{-5}	1.0	2.0
**80 eq**	4.21×10^{-6}	5.2	10.4

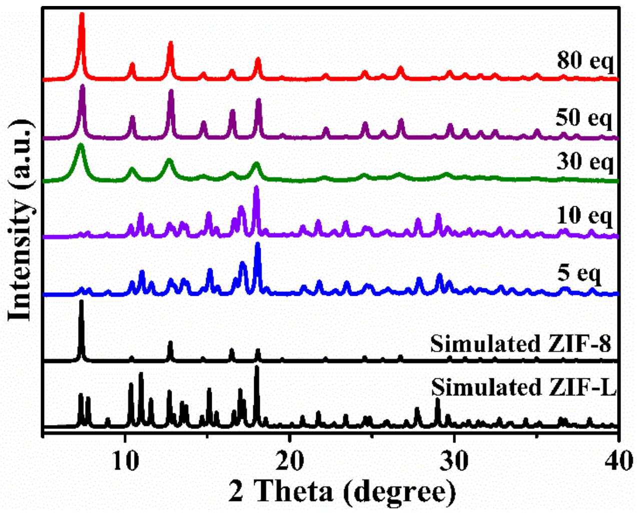

Figure S7. PXRD pattern of ZIFs synthesized with 2-HmIM at different concentration (1 eq = 0.02 mol L^{-1}).

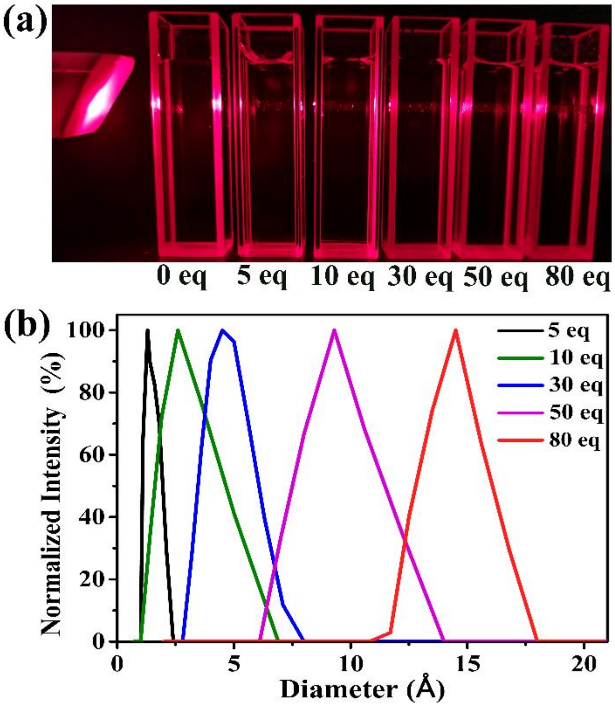

Figure S8. 2-HmIM micelles analysis. (a) Tyndall effect diagrams of 2-HmIM micelles at different concentrations (from left to right, H_2O, 5 eq, 10 eq, 30 eq, 50 eq and 80 eq of 2-HmIM, 1 eq = 0.02 mol L^{-1}), and (b) DLS analysis of 2-HmIM micelles at different concentrations (1 eq = 0.02 mol L^{-1}).

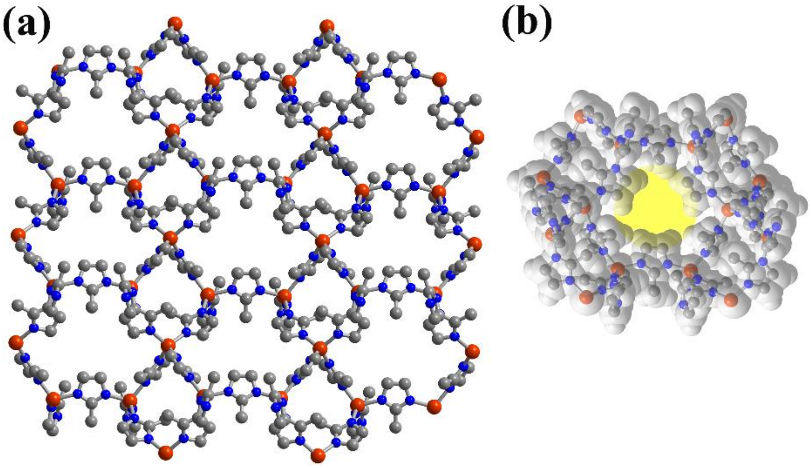

Figure S9. ZIF-L structure analysis. (a) 2D layer structure in ZIF-L. (b) A cushion-shaped cavity between layers in ZIF-L.

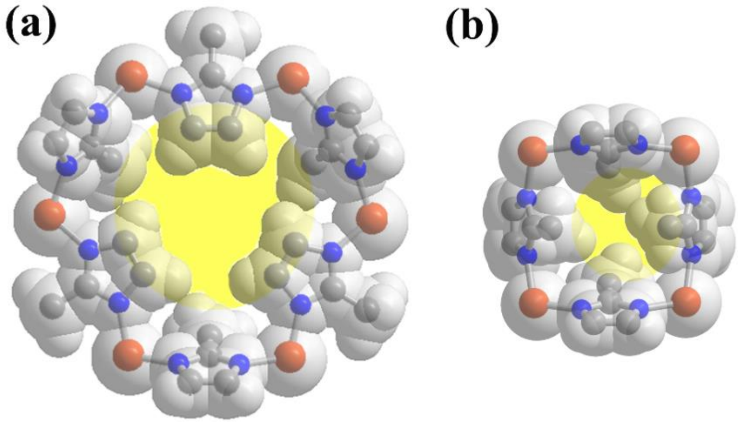

Figure S10. ZIF-8 structure analysis. (a) Structure of SOD cage of ZIF-8, and the yellow sphere represents the free volume within the framework. (b) 6-membered ring aperture of ZIF-8.

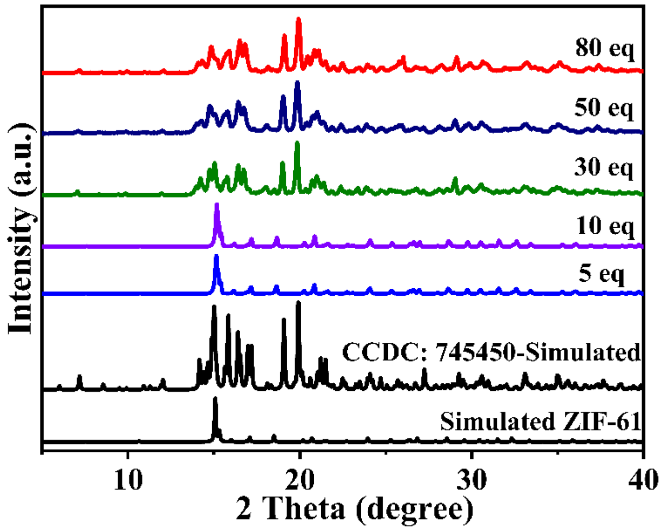

Figure S11. PXRD pattern of ZIFs synthesized with HIM at different concentration (1 eq = 0.02 mol L^{-1}).

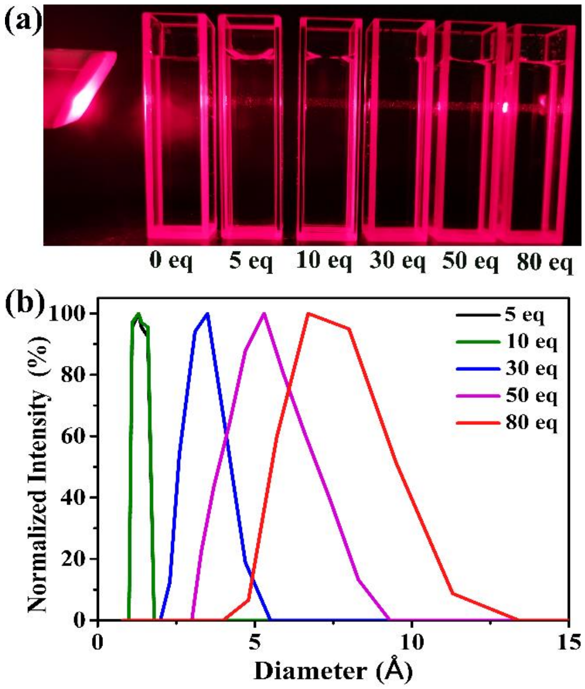

Figure S12. HIM micelles analysis. (a) Tyndall effect diagrams of HIM micelles at different concentrations (from left to right, H_2O, 5 eq, 10 eq, 30 eq, 50 eq and 80 eq of HIM, 1 eq = 0.02 mol L^{-1}), and (b) DLS analysis of HIM micelles at different concentrations (1 eq = 0.02 mol L^{-1}).

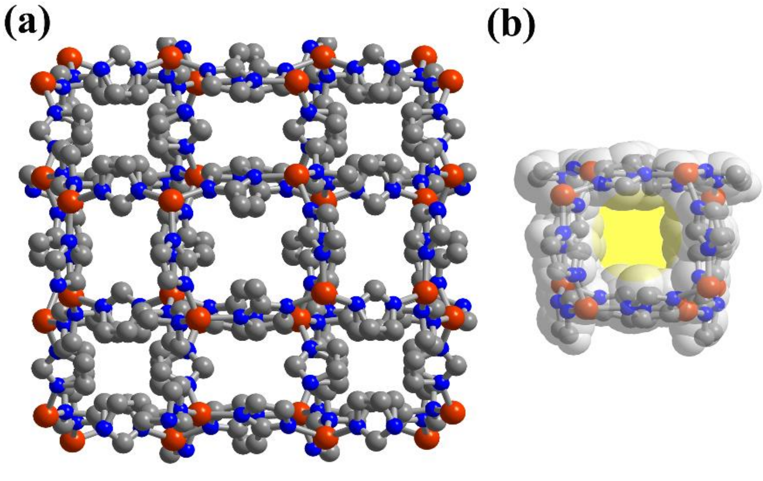

Figure S13. ZIF-61 structure analysis. (a) Zni topology structure of ZIF-61. (b) 5-membered ring aperture of ZIF-61.

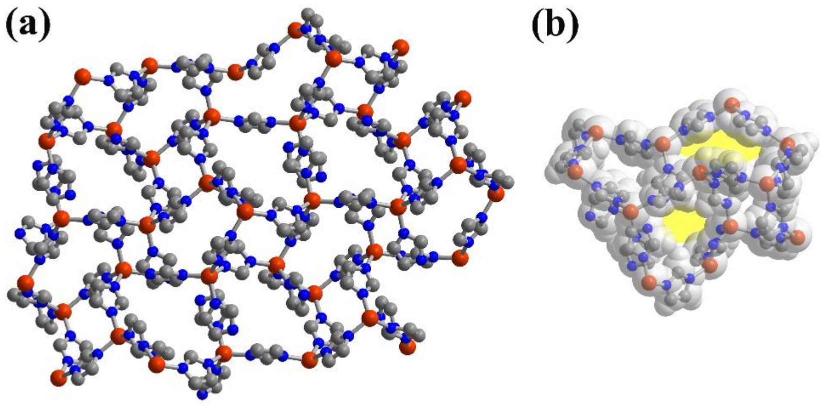

Figure S14. ZIF structure analysis. (a) Structure of dense ZIF (CCDC: 745450), containing primarily 4-membered ring units, such as many dense 4-link networks. (b) A dense phase with no porosity in ZIF.

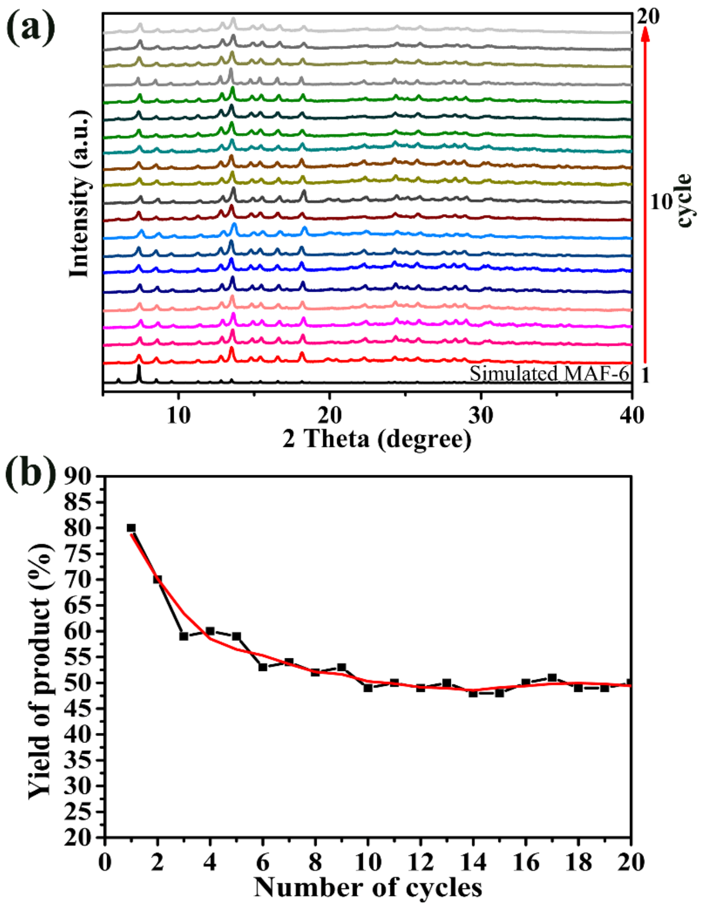

Figure S15. 20-cycle synthesis of MAF-6. (a) The PXRD patterns of 20-cycle synthesis of MAF-6 with 80 eq mother liquor. (b) Yields of MAF-6 20-cycle synthesis.

Section 4. Synthesis and Characterization of BCL@MAF-6

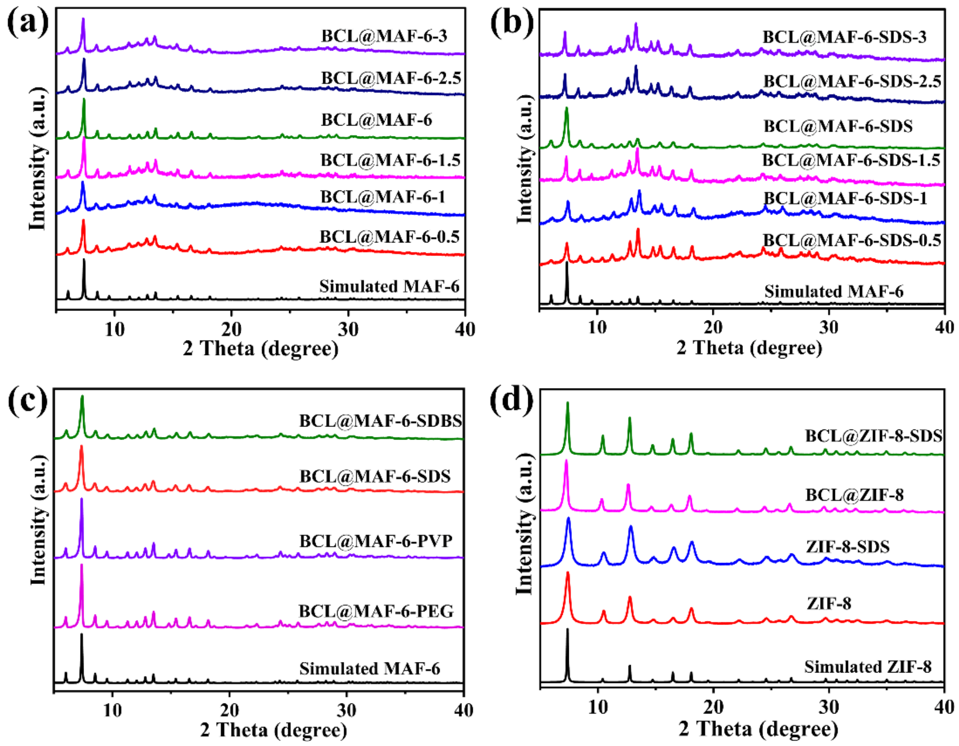

Figure S16. PXRD patterns of the synthesized samples. (a) BCL@MAF-6-X, (b) BCL@MAF-6-SDS-X, (c) BCL@MAF-6-surfactants and (d) ZIF-8, ZIF-8-SDS, BCL@ZIF-8, BCL@ZIF-8-SDS.

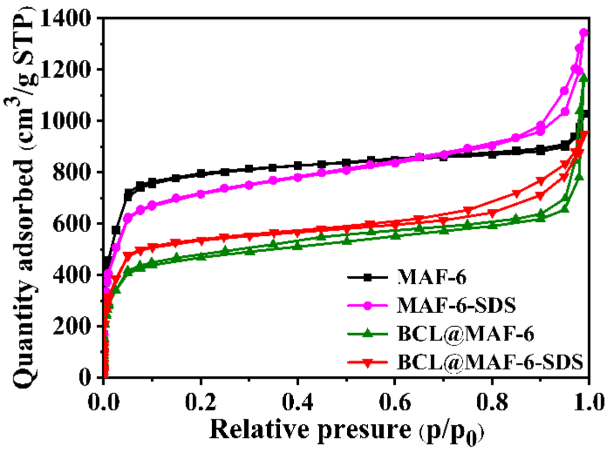

Figure S17. N_2 adsorption and desorption isotherms (77 K) of MAF-6, MAF-6-SDS, BCL@MAF-6 and BCL@MAF-6-SDS.

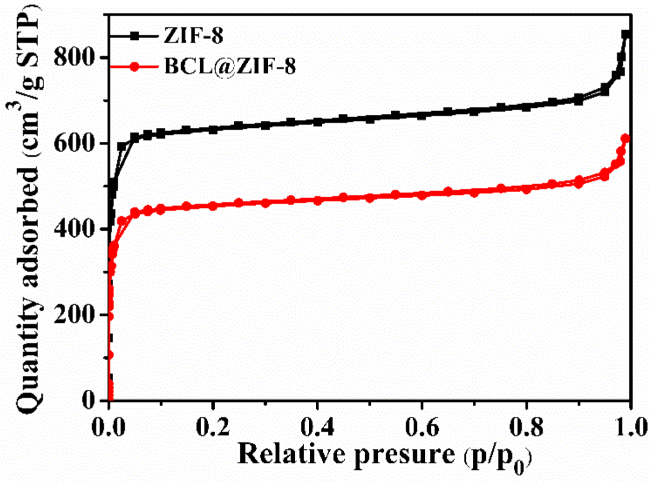

Figure S18. N_2 adsorption and desorption isotherms (77 K) of ZIF-8 and BCL@ZIF-8.

Table S2. BET surface areas of MAF-6, MAF-6-SDS, BCL@MAF-6, BCL@MAF-6-SDS, ZIF-8 and BCL@ZIF-8.

Samples	Surface areas (m^2 g^{-1})
**MAF-6**	1809.1
**MAF-6-SDS**	1875.1
**BCL@MAF-6**	1219.9
**BCL@MAF-6-SDS**	1297.9
**ZIF-8**	1573.3
**BCL@ZIF-8**	1134.9

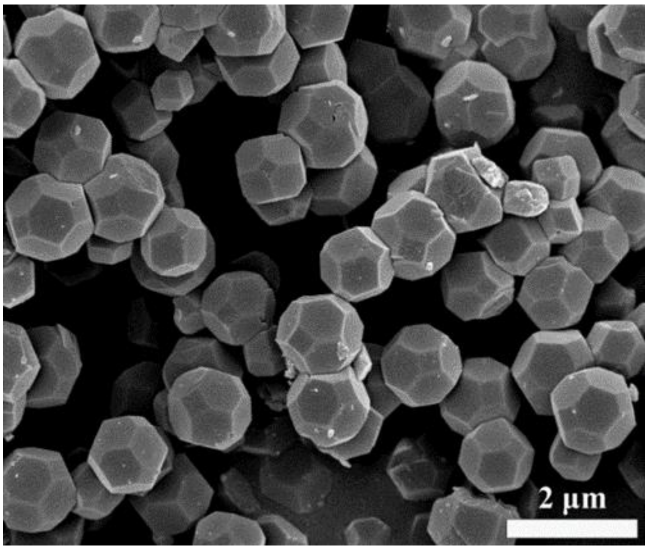

Figure S19. SEM image of MAF-6.

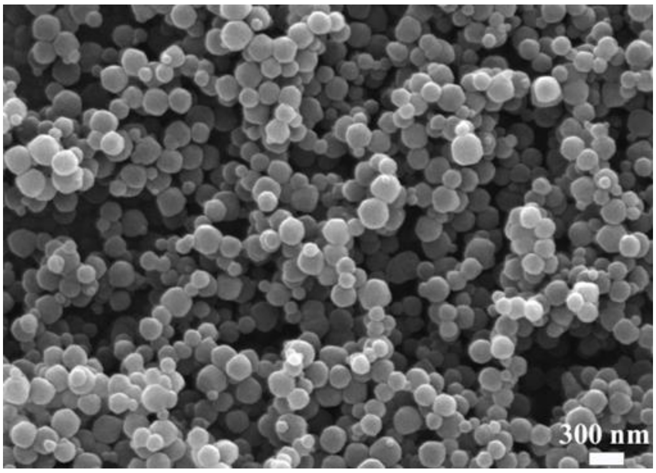

Figure S20. SEM image of ZIF-8.

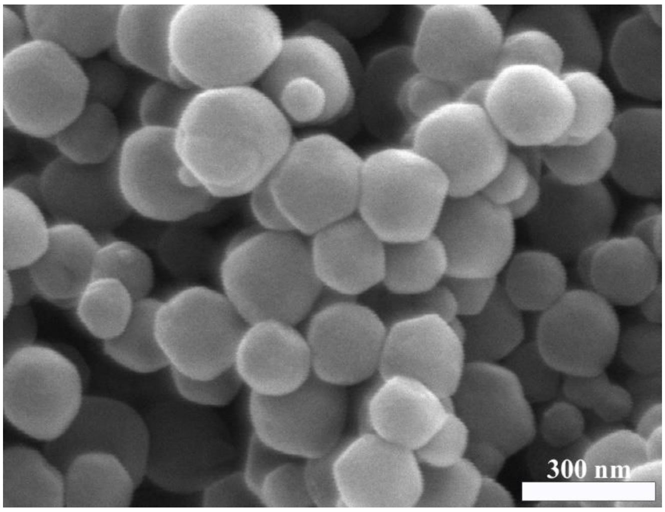

Figure S21. SEM image of BCL@ZIF-8.

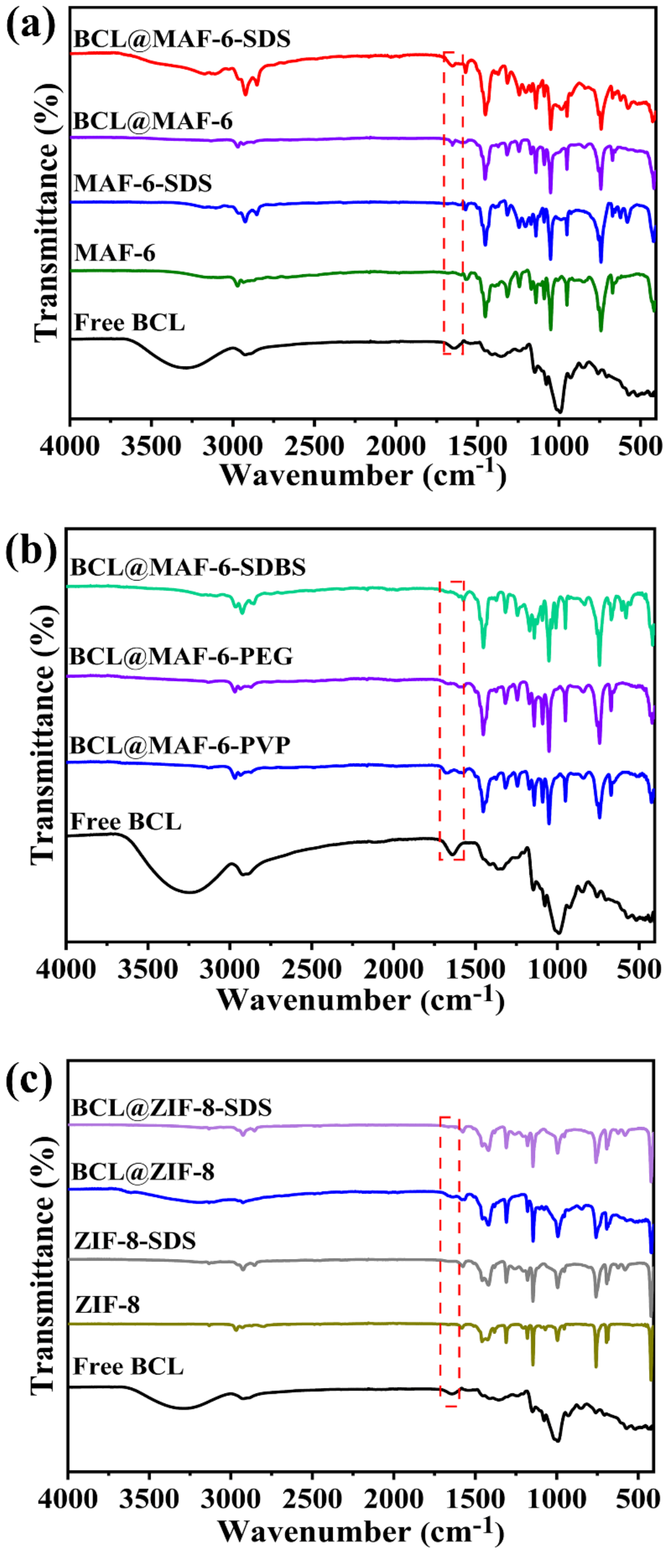

Figure S22. FTIR spectra of samples. (a) Free BCL, MAF-6, MAF-6-SDS, BCL@MAF-6, BCL@MAF-6-SDS. (b) Free BCL, BCL@MAF-6-PVP, BCL@MAF-6-PEG, BCL@MAF-6-SDBS. (c) Free BCL, ZIF-8, ZIF-8-SDS, BCL@ZIF-8, BCL@ZIF-8-SDS.

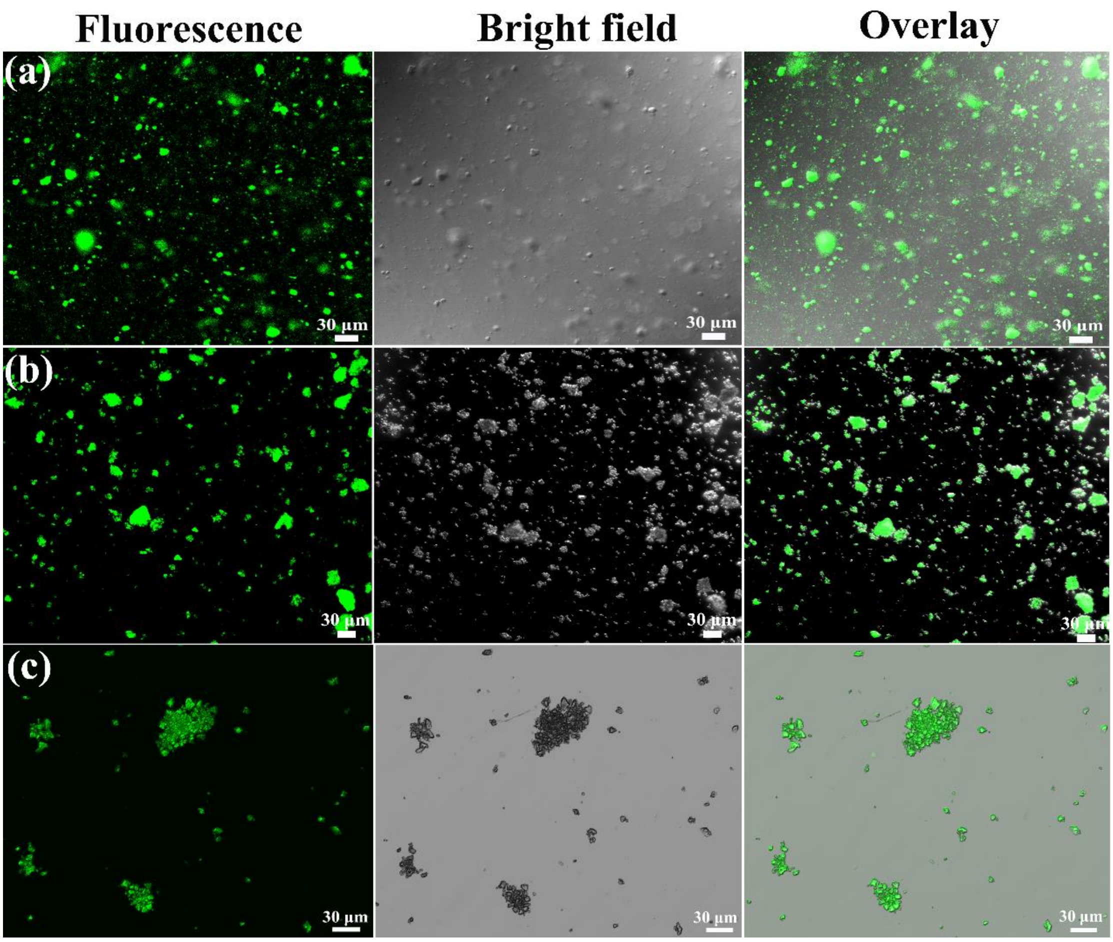

Figure S23. Confocal laser scanning microscopy (CLSM) images of samples. (a) FITC-BCL@ZIF-8. (b) FITC-BCL@MAF-6 and (c) FITC-BCL@MAF-6-SDS (excitation wavelength: 488 nm).

SDS–PAGE analysis of BCL@MAF-6, BCL@MAF-6-SDS, BCL/MAF-6 and BCL@ZIF-8.

All samples (40 mg for each sample) were first incubated in high concentration denaturation solution (sodium dodecyl sulfate, 10 wt%) for 2 h, then washed with ultrapure water for 5 times and freeze-dried. Next, the material was dispersed in a small amount of ultrapure water (2 mL), digested with concentrated hydrochloric acid (10–20 µL), washed 5 times with an ultrafiltration centrifuge tube (Millipore, 15 mL/10K, 4000 rpm for 25 min), and finally the volume of the residual solution was fixed to 500 µL. The above concentrates (16 µL) were added to 4 µL of buffer (5 X Protein Loading Buffer), heated at 95 ˚C for 5–10 minutes, and then centrifuged (12000 rpm, 5 min). Then, the centrifuged solution was applied to a protein precast gel, and the standard proteins mixture was used as the marker. Subsequently, the prepared protein precast gel samples were electrophoresed on SDS–PAGE at 100 V

for 10 minutes, then raised to 140 V for 50 minutes, then dyed with Commassie Blue R250, and finally washed to remove the floating color on the surface.

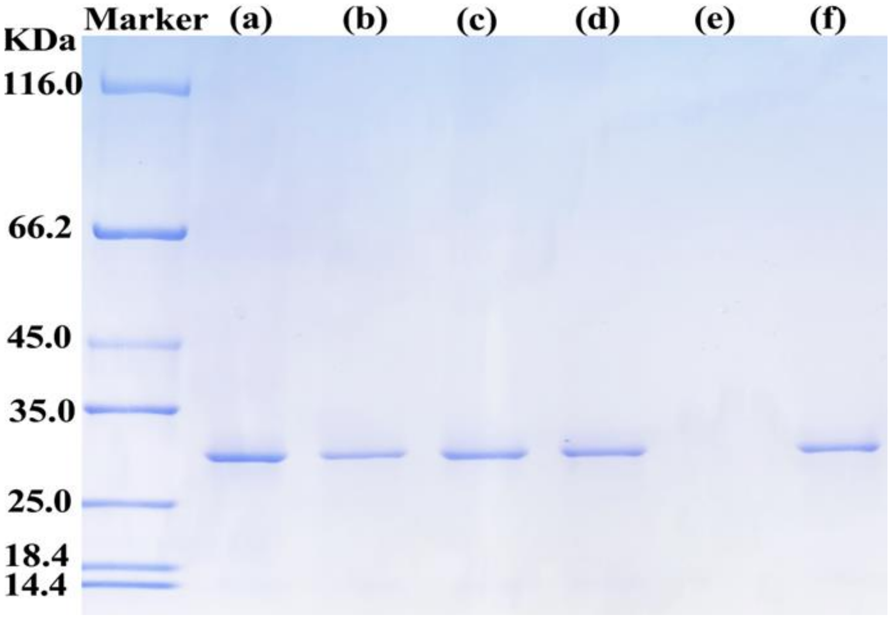

Figure S24. SDS–PAGE gel images of the samples from (a) Free BCL, (b) BCL@MAF-6, (c) BCL@MAF-6-SDS, (d) BCL/MAF-6 samples not incubated in denaturation solution, (e) BCL/MAF-6 and (f) BCL@ZIF-8, (Note: all the samples, except d, were incubated in denaturation solution).

Section 5. Optimization of Biocatalyst BCL@MAF-6.

Determination of encapsulation efficiency and loading capacity of biocomposites.

BCL concentration was measured *via* BCA method (The working reagent was mixed with BCA Reagent A (5 mL) and BCA Reagent B (Cu^{2+} solution, 100 μL)).

The following was the procedure detail.

1) The sample that encapsulated certain amount BCL in BCL@ZIF-8, BCL@ZIF-8-SDS, BCL@MAF-6 or BCL@MAF-6-X (X = PVP, PEG, SDS, SDBS) was dispersed in 2 mL of ultrapure water and the frameworks were digested by adding trace amounts of concentrated HCl (5–10 μL). The mixture was stirred for 20 min and concentrated by centrifugation through an ultrafiltration centrifugal tube (Millipore, 15 mL/ 10 K, 4000 rpm) and washed at least 4 times. At last, concentrate the solution in the ultrafiltration centrifugal tube to 500 μL. The standard and unknown sample solutions (50 μL) were added in a 96–well plate.

2) 200 μL of the working reagent was added to the above reagent and mixed well, followed by the incubation at 37 °C for 30 min.

3) The absorbance of the sample was measured at 562 nm UV-Vis, and the concentration of enzyme in the samples was calculated by the working curve (Figure A).

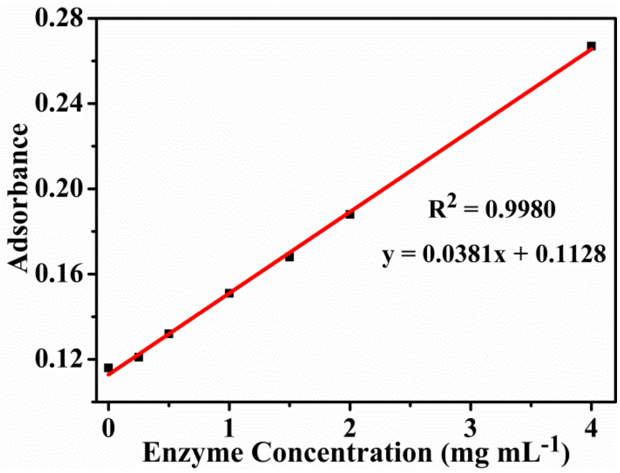

Figure A. The standard working curve shows the UV absorbance with different concentrations of lipase BCL using BCA methods.

Table S3. The loading capacity of BCL@ZIFs, calculated *via* BCA method.

Number	BCL@ZIFs	The input of BCL (mg mL^{-1})	Loading Capacity (mg mg^{-1})
**1**	BCL@MAF-6-0.5	0.5	0.04
**2**	BCL@MAF-6-1	1.0	0.10
**3**	BCL@MAF-6-1.5	1.5	0.13
**4**	BCL@MAF-6	2.0	0.18
**5**	BCL@MAF-6-2.5	2.5	0.23
**6**	BCL@MAF-6-3	3.0	0.28
**7**	BCL@MAF-6-SDS-0.5	0.5	0.11
**8**	BCL@MAF-6-SDS-1	1.0	0.15
**9**	BCL@MAF-6-SDS-1.5	1.5	0.17
**10**	BCL@MAF-6-SDS	2.0	0.20
**11**	BCL@MAF-6-SDS-2.5	2.5	0.26
**12**	BCL@MAF-6-SDS-3	3.0	0.32
**13**	BCL@ZIF-8-2	2.0	0.13
**14**	BCL@ZIF-8	2.5	0.19
**15**	BCL@ZIF-8-3	3.0	0.31
**16**	BCL@ZIF-8-SDS-2	2.0	0.16
**17**	BCL@ZIF-8-SDS	2.5	0.21
**18**	BCL@ZIF-8-SDS-3	3.0	0.32
**19**	BCL/MAF-6	2.0	0.20
**20**	BCL/MAF-6-SDS	2.0	0.20
**21**	BCL@MAF-6-PVP	2.0	0.18
**22**	BCL@MAF-6-PEG	2.0	0.19
**23**	BCL@MAF-6-SDBS	2.0	0.21
**24**	BCL@MAF-32	2.0	0.06
**25**	BCL@MAF-5	2.0	0.22
**26**	Cyt c@MAF-6-SDS	2.0	0.05
**27**	GOx@MAF-6-SDS	2.0	0.08
**28**	CAL–B@MAF-6-SDS	2.0	0.21

Enzyme's activity tests.

Hydrolysis profiles of *p*-nitrophenyl butyrate (NPB) and Phenyl ester (phenyl butyrate, *p*-tolyl butyrate, 4-ethylphenyl butyrate, 4-isopropylphenyl butyrate and 4-(tert-butyl)phenyl butyrate) by using free BCL or immobilized BCL were recorded on spectrophotometer.[5-8]

NPB and Phenyl ester solution (100 mM) were prepared in DMSO. The free BCL solution and the immobilized BCL suspension were normalized to the enzyme BCL concentration of 1 mg/mL in 50 mM Tris–HCl buffered saline (TBS, pH=7.0).

For each NPB hydrolysis reaction, 970 μL of TBS, 10 μL of free BCL solution or BCL@ZIFs suspension, and 20 μL of NPB solution were added in 10 mL centrifuge tube (1 mL in total) and this solution was heated 40 °C for 4 min. The absorbance of the solution was measured by Multiskan SkyHigh at 405 nm. The concentration of the product *p*-nitrophenol was calculated from the standard curve (Supplementary Figure B a).

For Phenyl ester hydrolysis reaction, the addition of Gibbs's reagent (20 mM in EtOH) were required due to the weak UV absorption of the product.[5-6] Configuration of phenol standard solution: All phenol solutions (phenol, *p*-cresol, 4-ethylphenol, 4-isopropylphenol and 4-tert-butylphenol) were prepared in DMSO with different solubility. The absorbance of 940 μL of TBS, 30 μL of phenol solution and 30 μL of Gibbs's reagent were incubated for 60 minutes at 37 °C and measured at 600 nm (phenol-Gibbs' adduct, Supplementary Figure B b–f).

For hydrolysis profiles of Phenyl ester, 950 μL of TBS, 20 μL of free BCL solution or BCL@ZIFs suspension and 30 μL of Phenyl ester solution (phenol-Gibbs' adduct) were added in 10 mL centrifuge tube (1 mL in total) and this solution was heated 40 °C for 60 min. The absorbance of the solution was measured by Multiskan SkyHigh at 600 nm. The concentration of the product phenol was calculated from the standard curve.

In order to reduce errors in experiments or make the experimental results more consistent, in parallel experiments, all the enzyme activities of immobilized BCL were recorded as the percentage of free BCL activity. The most importantly, all reactions were repeated at least 3 times.

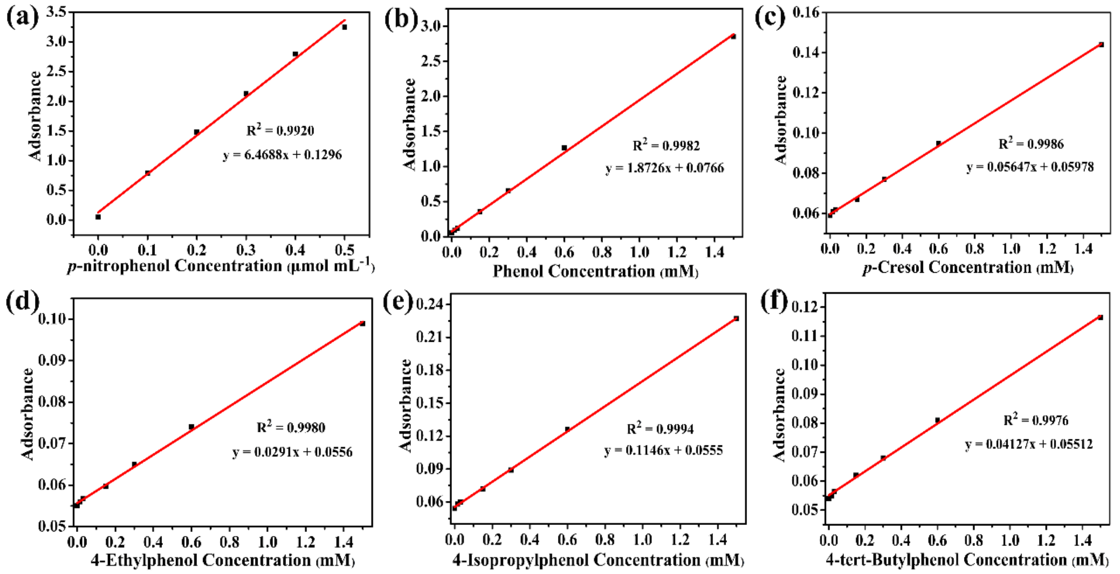

Figure B. The standard curve of the UV absorbance *vs.* different concentrations of phenol. (a) *p*-nitrophenol, (b) phenol, (c) *p*-cresol, (d) 4-ethylphenol, (e) 4-isopropylphenol, (f) 4-tert-butylphenol.

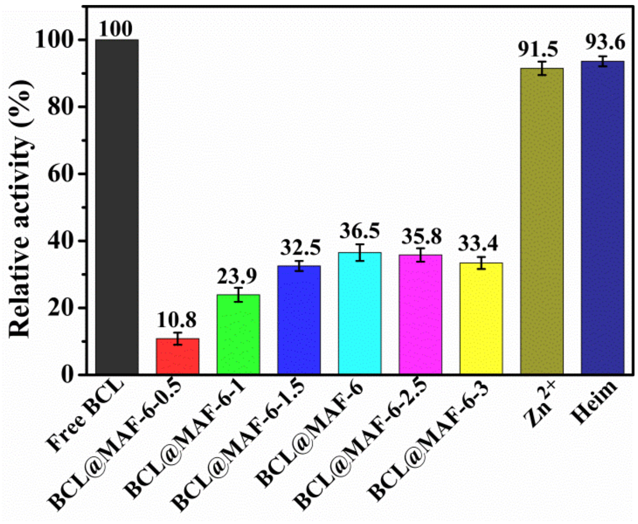

Figure S25. Enzymatic activity of different samples and enzymatic activity of free BCL after incubating with Zn^{2+} ions and HeIM solution. (BCL@MAF-6-X, X= the amounts of BCL input in the ZIF synthesis; the input of BCL@MAF-6 is 2).

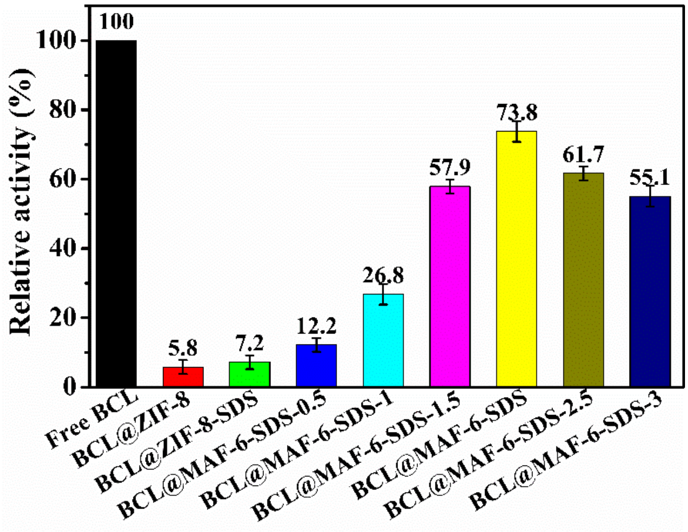

Figure S26. Enzymatic activity of different samples. Free BCL, BCL@ZIF-8, BCL@ZIF-8-SDS and BCL@MAF-6-SDS-X (X= the amounts of BCL input in the ZIF synthesis; the input of BCL@MAF-6-SDS is 2).

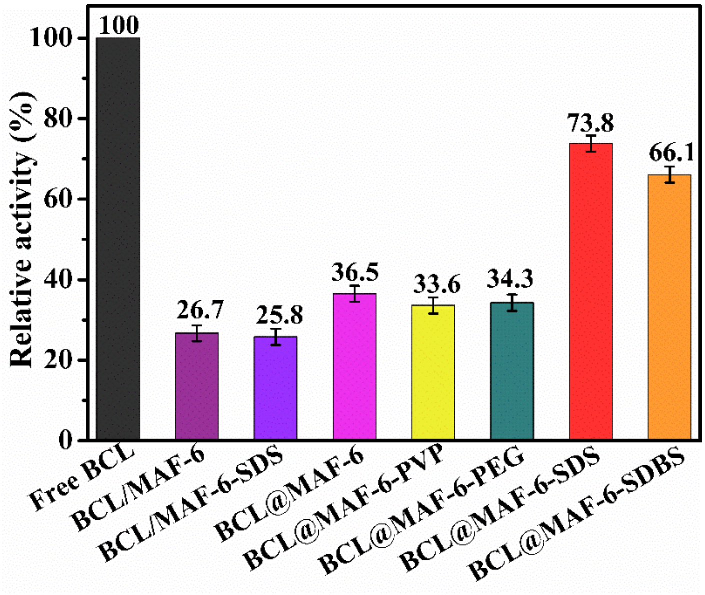

Figure S27. Enzymatic activity of different samples. Free BCL, BCL/MAF-6, BCL/MAF-6-SDS, BCL@MAF-6, BCL@MAF-6-PVP, BCL@MAF-6-PEG, BCL@MAF-6-SDS, BCL@MAF-6-SDBS.

SDS content in BCL@MAF-6-SDS was determined. 5 mg of BCL@MAF-6-SDS was dispersed into deuterated water, digested by adding trace deuterated HCl, and then measured by ^{1}H NMR spectra. It can be seen from the digested BCL@MAF-6-SDS ^{1}H NMR spectra that the SDS molar content is 8% of HeIM. Then, according to the content of BCL measured by the BCA method, the weight ratio of SDS to BCL can be calculated as 0.8:1.

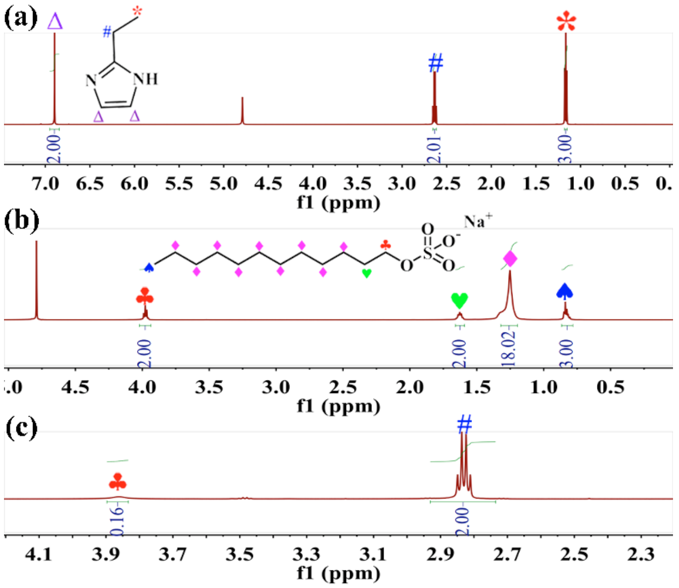

Figure S28. ^{1}H NMR spectra of (a) HeIM, (b) SDS and (c) BCL@MAF-6-SDS digestion sample.

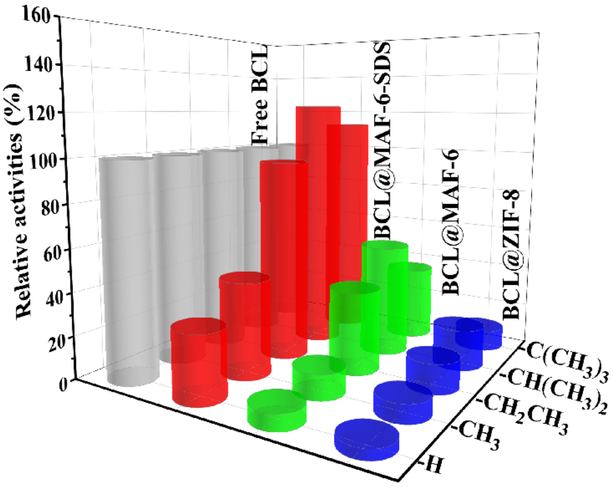

Figure S29. The relative activities of free BCL, BCL@MAF-6-SDS, BCL@MAF-6 and BCL@ZIF-8 catalysts in a series of phenolic esters hydrolysis reactions.

Table S4. The product concentration in phenol ester hydrolysis reactions catalyzed by free BCL, BCL@MAF-6-SDS, BCL@MAF-6 and BCL@ZIF-8. The esters are phenyl butyrate, *p*-tolyl butyrate, 4-ethylphenyl butyrate, 4-isopropylphenyl butyrate and 4-(tert-butyl)phenyl butyrate.

Phenyl ester	Free BCL (mM)	BCL@MAF-6-SDS (mM)	BCL@MAF-6 (mM)	BCL@ZIF-8 (mM)
**-H**	0.57	0.18	0.04	0.03
**-CH_3**	0.26	0.12	0.03	0.02
**-C_2H_5**	0.42	0.40	0.16	0.06
**-$CH(CH_3)_2$**	0.90	1.10	0.47	0.13
**-$C(CH_3)_3$**	0.59	0.66	0.21	0.06

Table S5. The conversion in phenol ester hydrolysis reactions catalyzed by free BCL and BCL@MAF-6-SDS. The esters are phenyl butyrate, *p*-tolyl butyrate, 4-ethylphenyl butyrate, 4-isopropylphenyl butyrate and 4-(tert-butyl)phenyl butyrate.

Phenyl ester	Free BCL (%)	BCL@MAF-6-SDS (%)
**-H**	38.00	12.00
**-CH_3**	17.33	8.00
**-C_2H_5**	28.00	26.67
**-$CH(CH_3)_2$**	60.00	73.33
**-$C(CH_3)_3$**	39.33	44.00

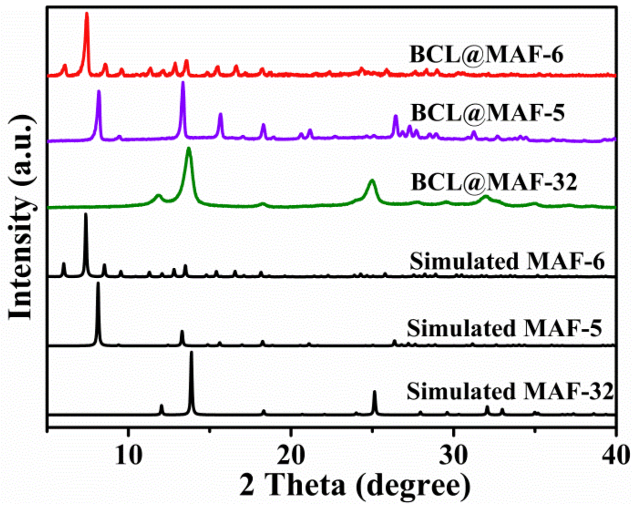

Figure S30. PXRD patterns of BCL@MAF-32, BCL@MAF-5 and BCL@MAF-6.

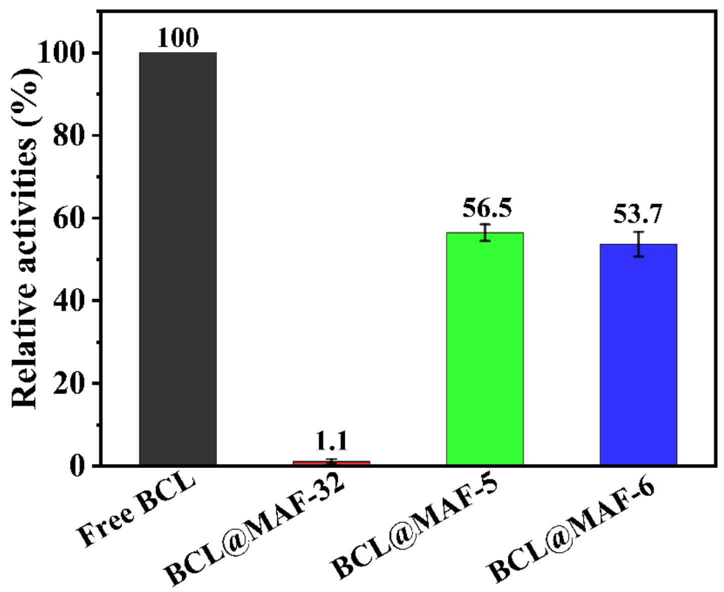

Figure S31. The relative activity of free BCL, BCL@MAF-32, BCL@MAF-5 and BCL@MAF-6 catalysts in hydrolysis of 4-isopropylphenyl butyrate.

Examination of enzymatic activities of free Cyt c and Cyt c@MAF-6-SDS, and free GOx and GOx@MAF-6-SDS.

For determining the enzymatic activities of free Cyt c and Cyt c@MAF-6-SDS, 2, 2'-azino-bis(3-ethylbenzothiazoline-6-sulfonicacid)-diammonium salt (ABTS) was used as the substrate. Prepare the 5 μL of free Cyt c solution and the immobilized Cyt c suspension (containing the same concentration of Cyt c of 0.1 mg mL^{-1}) in 240 μL of ABTS solution (2.8 mg mL^{-1} ABTS in TBS buffer, pH 7.0) and 5 μL of H_2O_2 (1 M) were added in a 96-well plate (250 μL in total). Then, the absorbance of the solution was measured at 415 nm for 20 min.

The enzymatic activities of free GOx and GOx@MAF-6-SDS were performed as follows: 10 μL of glucose solution (0.5 M) and 10 μL of ABTS solution (0.02 M in TBS buffer, pH 7.0) in 280 μL of TBS solution (containing the same concentration of GOx of 40 μg mL^{-1} and 20 μg mL^{-1} of HRP) were added in a 96-well plate (300 μL in total). Then, the absorbance of the solution was measured at 415 nm for 10 min.

The enzyme activities of immobilized Cyt c or GOx were recorded as the percentage of free Cyt c or GOx activity. For each reaction should be repeated at least 3 times. And, the activity of Cyt c and Cyt

c@MAF-6-SDS or GOx and GOx@MAF-6-SDS was calculated from the slope of the absorbance *vs.* time plot.[9]

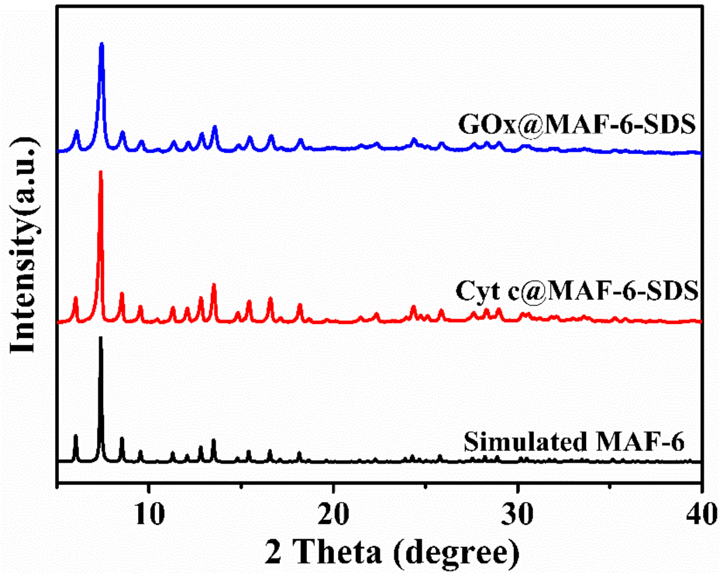

Figure S32. PXRD patterns for Cyt c@MAF-6-SDS and GOx@MAF-6-SDS.

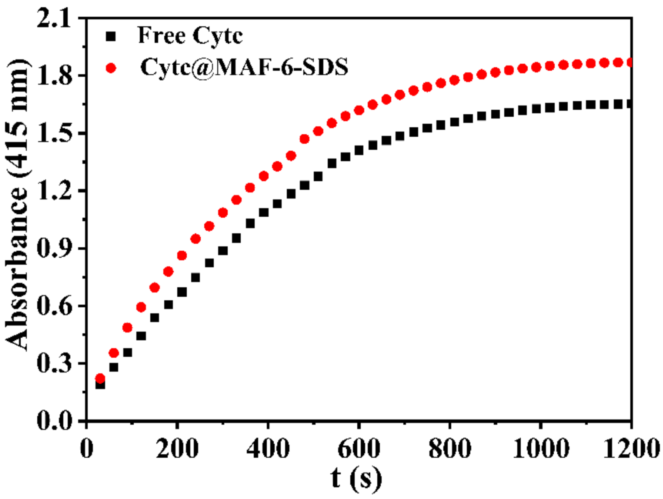

Figure S33. Curves for absorbance of ABTS oxidation products at 415 nm by free Cyt c and Cyt c@MAF-6-SDS.

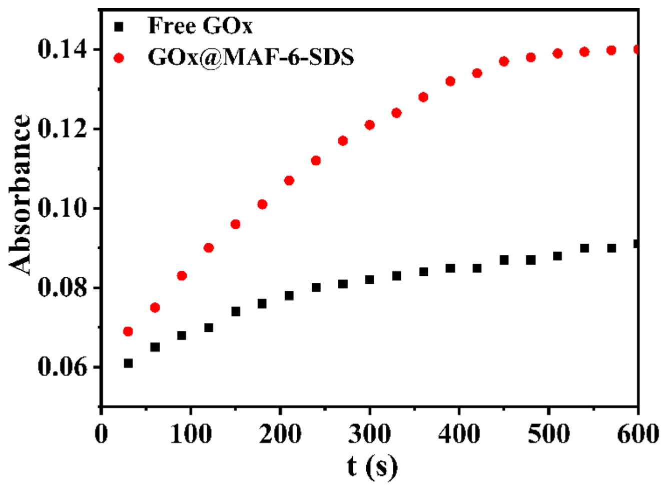

Figure S34. Curves for absorbance of glucose and ABTS oxidation products at 415 nm by free GOx and GOx@MAF-6-SDS.

Section 6. Asymmetric Catalysis with BCL@MAF-6-SDS

Kinetic resolution of racemic 4-phenyl-3-buten-2-ol with vinyl acetate.

As a typical run, 4-phenyl-3-buten-2-ol (0.3 mmol), 2 mg catalyst (based on BCL), vinyl acetate (0.75 mmol) and 2.5 mL *n*-hexane were added into a 10 mL test tube, then the mixture was transferred to 45 °C oil bath and stirred for a period of time. After the reaction, the catalyst was recovered, the yield of the product was analyzed by ^{1}H NMR and the *ee* values were determined by HPLC with a chiralcel OD–H column. The recyclable catalyst was washed with *n*-hexane, dried overnight in vacuum at room temperature, and directly used for the next run.

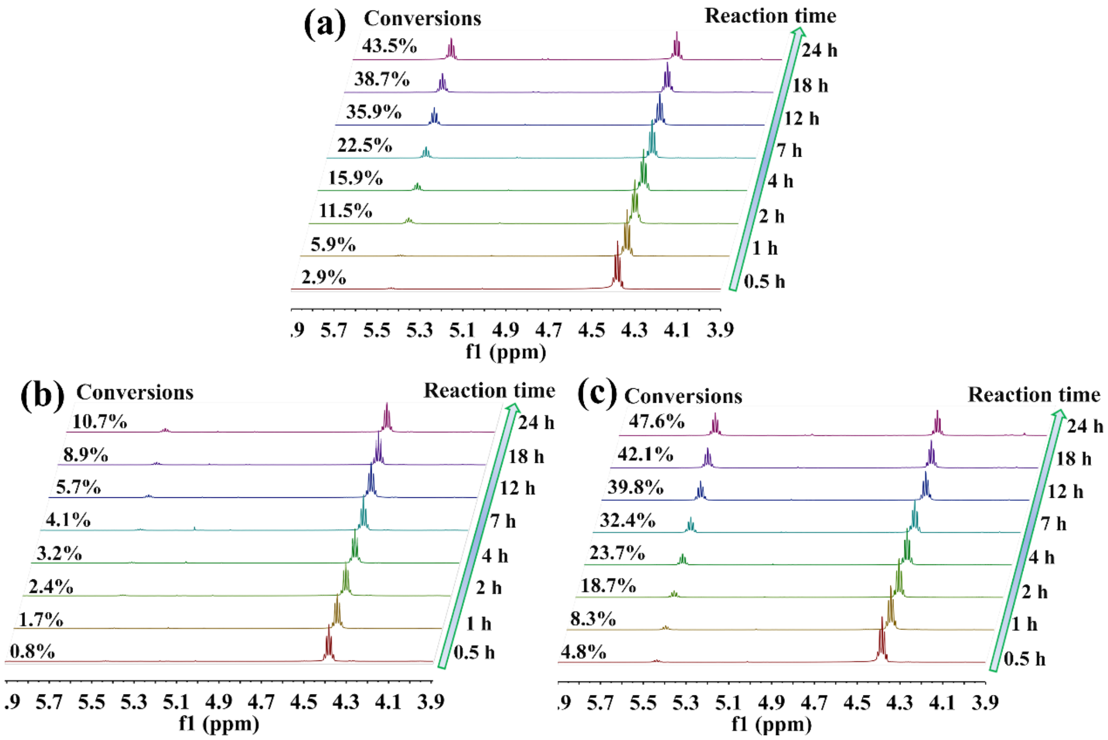

Figure S35. The yield of sample was measured by NMR spectroscopy. (a) Free BCL, (b) BCL@MAF-6 (c) BCL@MAF-6-SDS as biocatalysts, and the yields were measured by NMR spectroscopy. The substrate 4-phenyl-3-buten-2-ol peaks at around 4.4 ppm and the product 4-phenyl-3-buten-2-yl acetate peaks at around 5.5 ppm.

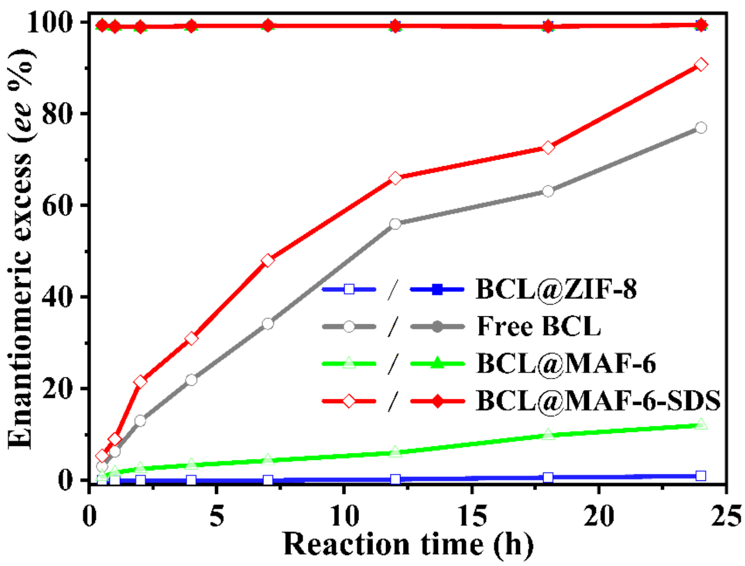

Figure S36. Enantiomeric excess values (*ee*) of substrate (hollow sphere) and product (solid sphere) with reaction time. The *ee* of the products are always > 99%.

Michaelis–Menten kinetic parameters.

The enzymatic kinetic constants of free and immobilized BCL in ZIFs were determined using the Michaelis–Menten enzymatic kinetics method:[10]

$$V_0 = \frac{V_{max}\,[S]}{K_m + [S]}$$

V_0 is the initial velocity; V_{max} is the maximum rate of the enzyme-catalyzed reaction when the catalytic sites are saturated with substrates, $[S]$ is the initial substrate concentration, and K_m is the Michaelis, which indicates the affinity to substrates. The kinetic constants K_m and V_{max} of the enzyme are measured by using the Lineweaver–Burk.[11] The Catalyst rate constant K_{cat} is equal to the quotient of V_{max} and enzyme concentration.

$$\frac{1}{V_0} = \frac{K_m}{V_{max}\,[S]} + \frac{1}{V_{max}}$$

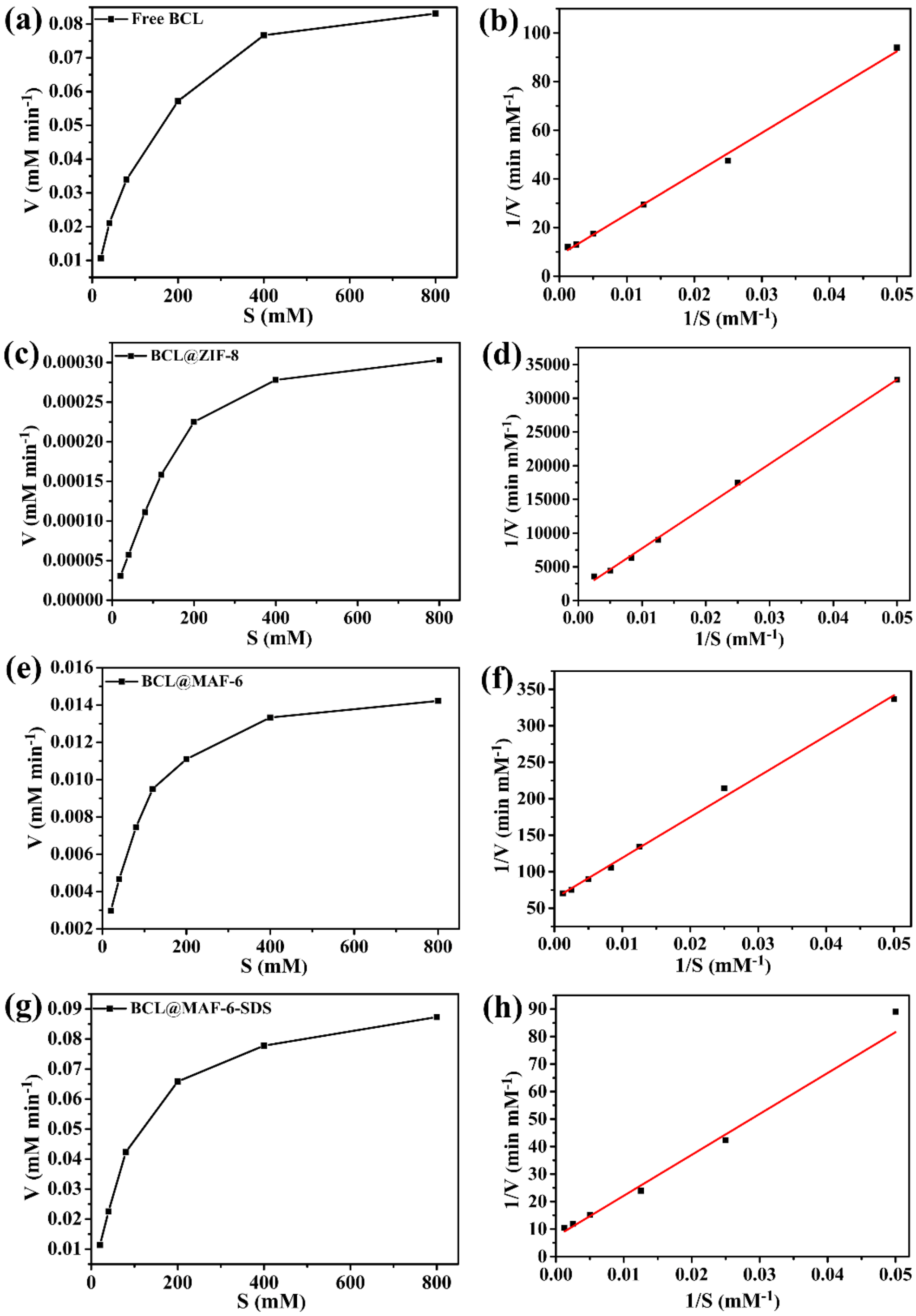

Figure S37. Michaelis–Menten plots of the effect of different concentrations of 4-phenyl-3-buten-2-ol on the initial velocity. (a) Free BCL, (c) BCL@ZIF-8, (e) BCL@MAF-6, (g) BCL@MAF-6-SDS. The Lineweaver–Burk plot for (b) Free BCL, R^2 = 0.9951, (d) BCL@ZIF-8, R^2 = 0.9981, (f) BCL@MAF-6, R^2 = 0.9943, (h) BCL@MAF-6-SDS, R^2 = 0.9861. The reaction conditions: biocatalyst (2 mg, based on BCL), 4-phenyl-3-buten-2-ol (0.00125 to 0.05 mM), 2.5 equivalent vinyl acetate and 2.5 mL *n*-hexane, and the reaction was carried out at 45 $^\circ$ C for 12 h.

Table S6. The Kinetic Parameters for free BCL, BCL@ZIF-8, BCL@MAF-6 and BCL@MAF-6-SDS.

Samples	V_{max} (mM min^{-1})	K_m (mM)	K_{cat} (min^{-1})^a	K_{cat}/K_m $\times 10^{-3}$ (min^{-1} mM^{-1})
Free BCL	0.12	207	5.03	24.4
BCL@ZIF-8	0.0007	422	0.03	0.066
BCL@MAF-6	0.02	88	0.65	7.41
BCL@MAF-6-SDS	0.14	204	5.65	27.7

^{a} $k_{cat} = V_{max}/[E]$ and $[E]$ represents the concentration of the enzyme, the molecular weight of BCL is 33 kDa.

Recycle experiments.

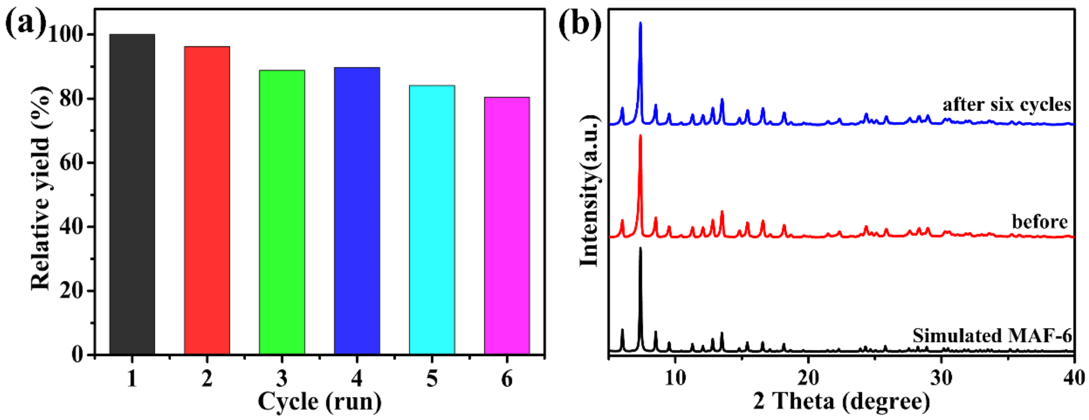

Figure S38. BCL@MAF-6 recycle reactions analysis. (a) The recycle reactions for BCL@MAF-6, (b) PXRD patterns of BCL@MAF-6, before and after six cycles.

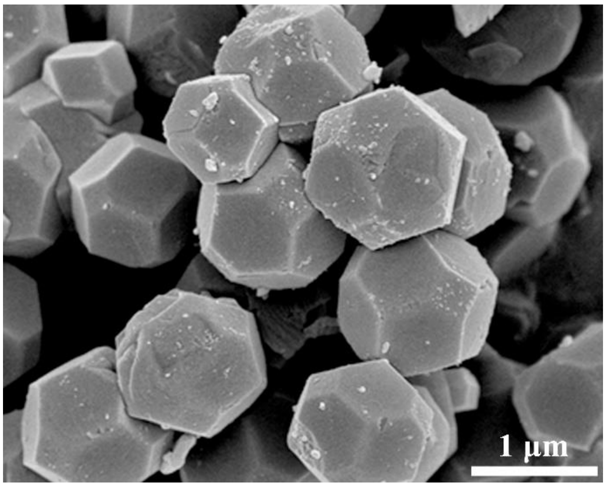

Figure S39. SEM of BCL@MAF-6 after six cycles.

Section 7. Catalytic Synthesis of Chiral Drug Precursor Molecules

Conditions for catalytic synthesis of chiral drug precursors:

Substrate (**1** to **6** (0.3 mmol) and **7** to **8** (0.2 mmol)), catalyst (based on BCL, **1** to **5** (2 mg) and **6** (8 mg); based on CAL-B, **7** to **8** (2 mg)), vinyl acetate (2.5 eq, 0.75 mmol) and 2.5 mL *n*-hexane were added into a 10 mL test tube. The mixture was heated at 45 °C oil bath and stirred for a period of time (24 h for **1**, **2** and **4**; 36 h for **3**; 33 h for **5**; 48 h for **6**; 24 h for free CAL-B and 36 h for CAL-B@MAF-6-SDS in **7**; 22 h for free CAL-B and 36 h for CAL-B@MAF-6-SDS in **8**.). After the reaction, the catalyst was filtered, and the excess solvent was removed by vacuum. Then the oil was dissolved in deuterated chloroform (0.5 mL). The yield of the product was calculated by ^{1}H NMR integration and the *ee* values were determined by HPLC with a chiralcel OD–H, OJ–H or AD–H column.

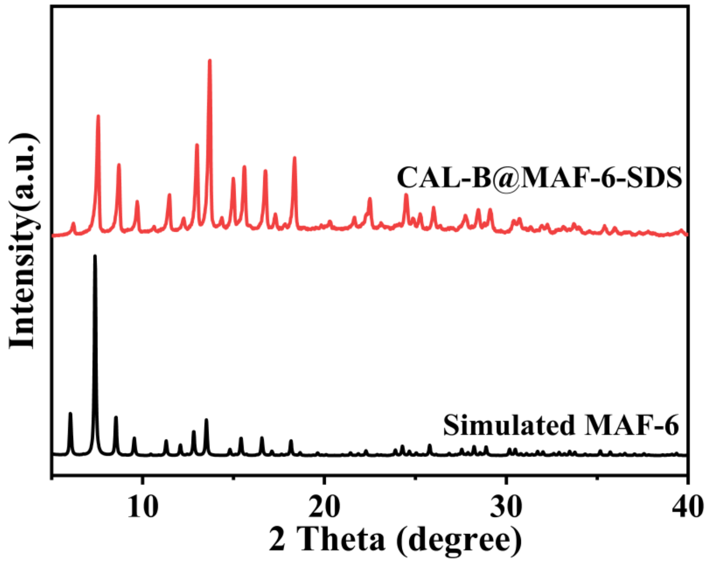

Figure S40. PXRD patterns of CAL-B@MAF-6-SDS and simulated MAF-6.

Table S7. The corresponding products required for substrate expansion.

Entry	Biocatalyst	Substrate	Product	Yield (%)	*ee* (%)
1	BCL@ZIF-8	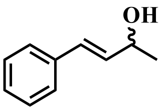	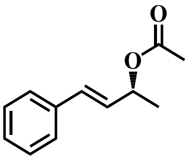 **1**	2.0	99.1
	BCL@MAF-6-SDS			47.6	99.2
	Free BCL			43.5	99.3
2	BCL@ZIF-8	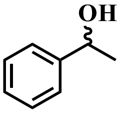	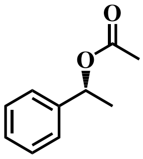 **2**	No reaction	None
	BCL@MAF-6-SDS			43.8	99.4
	Free BCL			41.9	99.5
3	BCL@ZIF-8	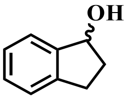	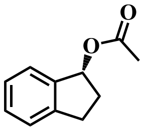 **3**	No reaction	None
	BCL@MAF-6-SDS			49.9	94.5
	Free BCL			49.9	95.1
4	BCL@ZIF-8	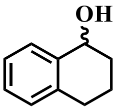	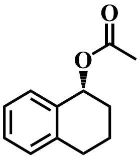 **4**	No reaction	None
	BCL@MAF-6-SDS			47.1	99.8
	Free BCL			45.1	99.8
5	BCL@ZIF-8	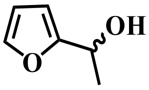	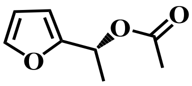 **5**	No reaction	None
	BCL@MAF-6-SDS			49.9	97.9
	Free BCL			46.9	98.7
6	BCL@ZIF-8	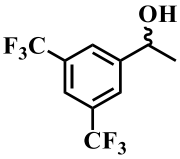	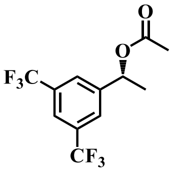 **6**	No reaction	None
	BCL@MAF-6-SDS			46.2	98.2
	Free BCL			43.6	98.9
7	CAL-B@ZIF-8	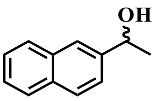	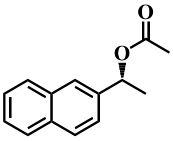 **7a**	No reaction	None
	CAL-B@MAF-6-SDS			28.1	98.8
	Free CAL-B			30.1	99.0
8	CAL-B@ZIF-8	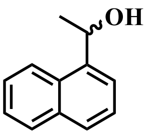	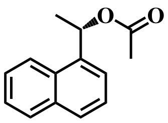 **8**	No reaction	None
	CAL-B@MAF-6-SDS			8.3	97.0
	Free CAL–B			47.9	98.9

Section 8. Thermal and Chemical Stability

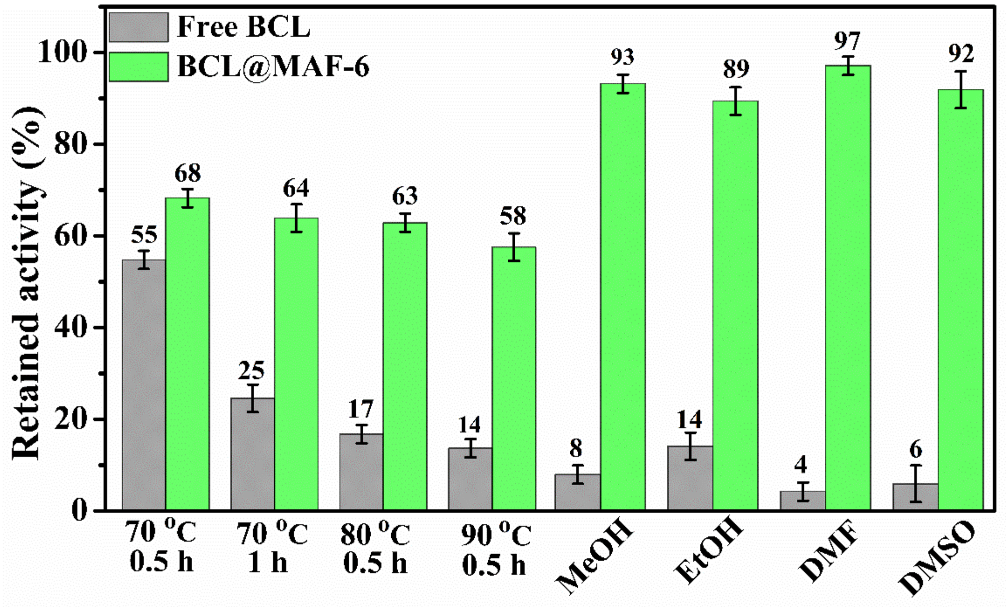

Figure S41. Retained activity of free BCL and BCL@MAF-6 after treatment under various harsh conditions.

Section 9. NMR Spectroscopy

Section 9.1. NMR spectra of substrates

phenyl butyrate: ^{1}H NMR (600 MHz, $CDCl_3$) δ 7.29 – 7.26 (m, 2H), 7.15 – 7.11 (m, 1H), 6.99 – 6.98 (m, 2H), 2.45 (t, J = 7.4 Hz, 2H), 1.73 – 1.67 (m, 2H), 0.95 (t, J = 7.4 Hz, 3H).

^{13}C NMR (151 MHz, $CDCl_3$) δ 172.25 (s), 150.87 (s), 129.50 (s), 125.81 (s), 121.70 (s), 36.36 (s), 18.59 (s), 13.76 (s).

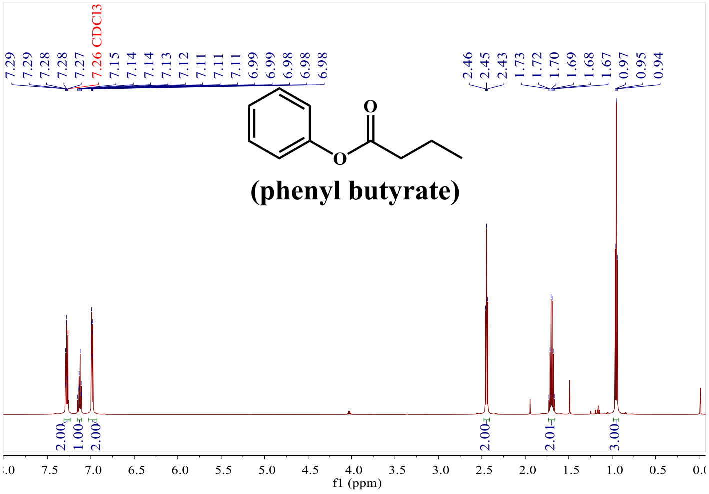

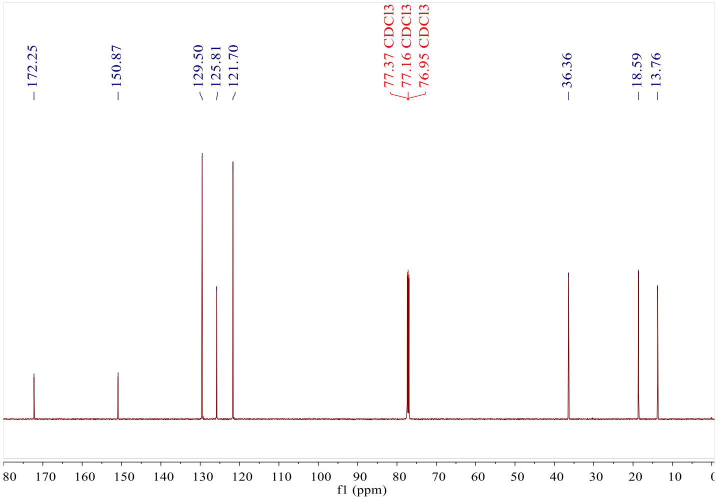

***p*–tolyl butyrate**: ^{1}H NMR (600 MHz, $CDCl_3$) δ 7.16 (d, *J* = 8.2 Hz, 2H), 6.95 (d, *J* = 8.4 Hz, 2H), 2.53 (t, *J* = 7.4 Hz, 2H), 2.34 (s, 3H), 1.82 – 1.76 (m, 2H), 1.05 (t, *J* = 7.4 Hz, 3H).

^{13}C NMR (151 MHz, $CDCl_3$) δ 172.40 (s), 148.62 (s), 135.38 (s), 129.98 (s), 121.34 (s), 36.33 (s), 20.93 (s), 18.59 (s), 13.73 (s).

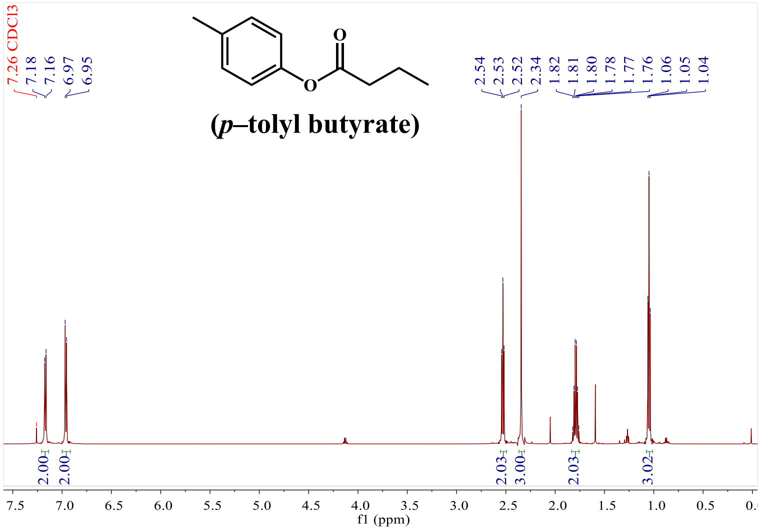

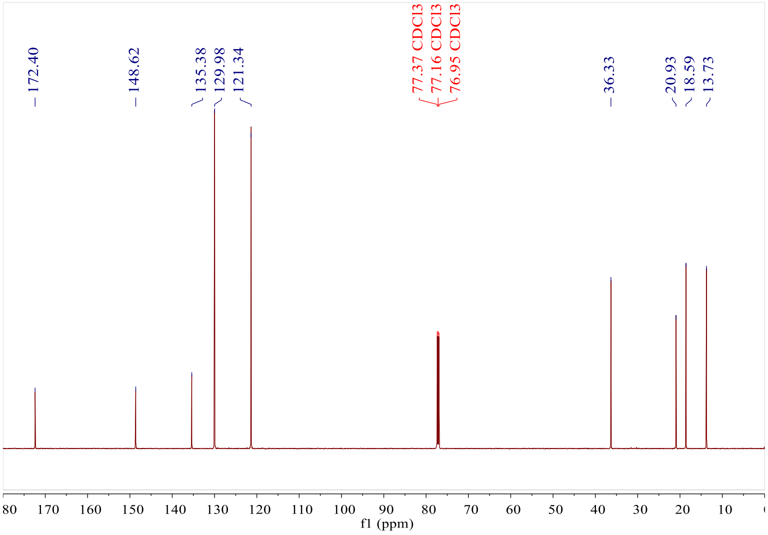

4–ethylphenyl butyrate: ^{1}H NMR (600 MHz, $CDCl_3$) δ 7.19 (d, *J* = 8.4 Hz, 2H), 6.99 (d, *J* = 8.4 Hz, 2H), 2.65 (q, *J* = 7.6 Hz, 2H), 2.54 (t, *J* = 7.4 Hz, 2H), 1.79 (dd, *J* = 14.8, 7.4 Hz, 2H), 1.24 (t, *J* = 7.6 Hz, 3H), 1.05 (t, *J* = 7.4 Hz, 3H).

^{13}C NMR (151 MHz, $CDCl_3$) δ 172.44 (s), 148.75 (s), 141.72 (s), 128.80 (s), 121.38 (s), 36.35 (s), 28.38 (s), 18.61 (s), 15.67 (s), 13.74 (s).

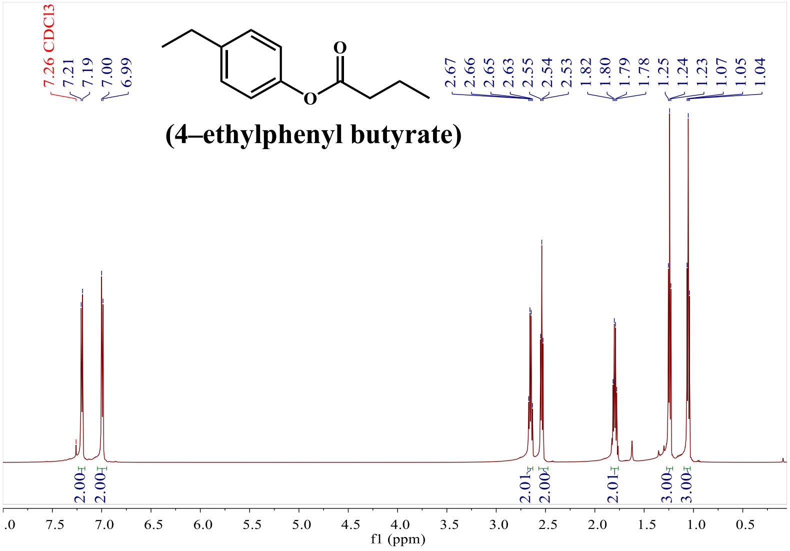

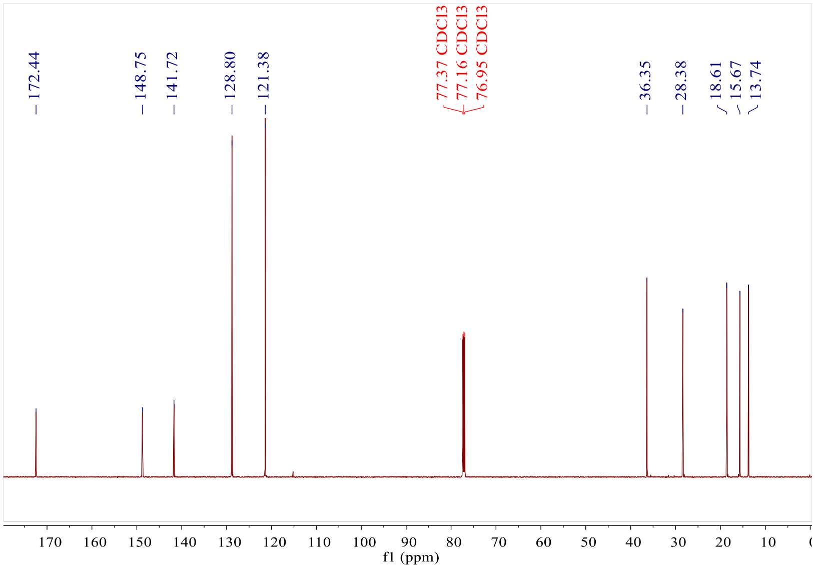

4–isopropylphenyl butyrate: ^{1}H NMR (600 MHz, $CDCl_3$) δ 7.23 (d, J = 8.4 Hz, 2H), 7.01 (d, J = 8.5 Hz, 2H), 2.90 (dt, J = 13.8, 6.9 Hz, 1H), 2.53 (t, J = 7.4 Hz, 2H), 1.83 – 1.77 (m, 2H), 1.25 (d, J = 7.0 Hz, 6H), 1.06 (t, J = 7.4 Hz, 3H).

^{13}C NMR (151 MHz, $CDCl_3$) δ 172.43 (s), 148.78 (s), 146.31 (s), 127.39 (s), 121.34 (s), 36.37 (s), 33.73 (s), 24.15 (s), 18.62 (s), 13.75 (s).

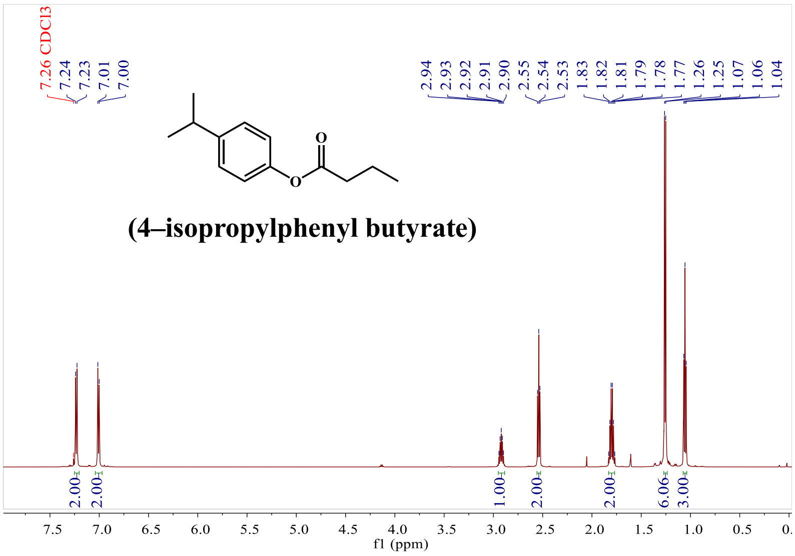

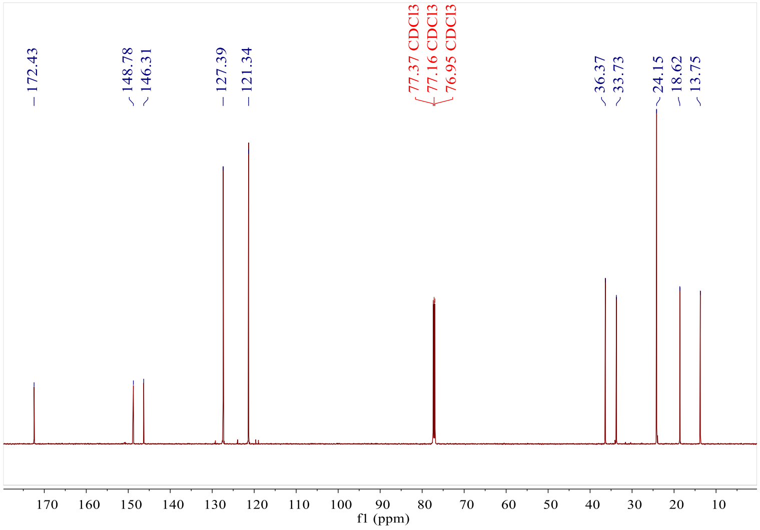

4–(tert–butyl)phenyl butyrate: ^{1}H NMR (600 MHz, $CDCl_3$) δ 7.40 (d, J = 8.7 Hz, 2H), 7.02 (d, J = 8.7 Hz, 2H), 2.54 (t, J = 7.4 Hz, 2H), 1.80 (dd, J = 14.8, 7.4 Hz, 2H), 1.34 (s, 9H), 1.07 (t, J = 7.4 Hz, 3H).

^{13}C NMR (151 MHz, $CDCl_3$) δ 172.36 (s), 148.50 (s), 126.35 (s), 120.97 (s), 36.35 (s), 34.54 (s), 31.51 (s), 18.60 (s), 13.73 (s).

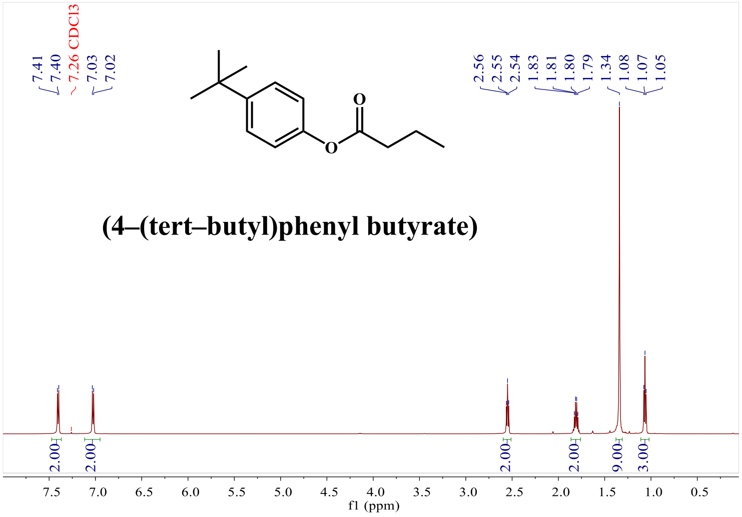

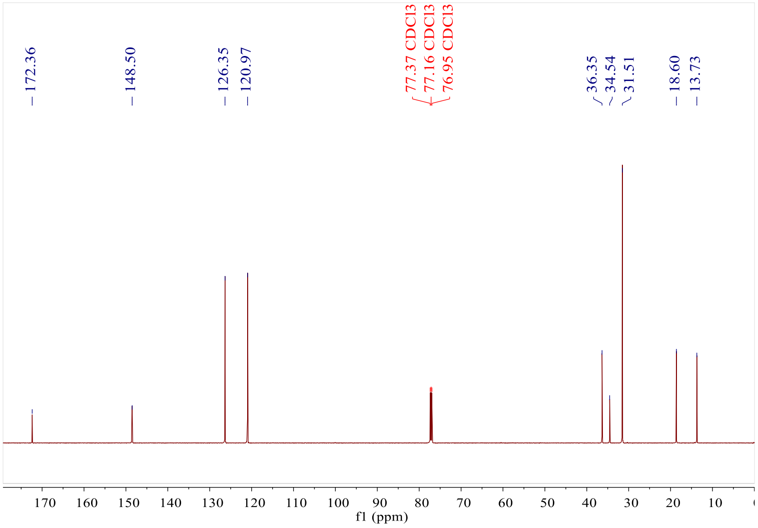

4–phenyl–3–buten–2–ol :^{1}H NMR (600 MHz, $CDCl_3$) δ 7.27 (d, *J* = 7.3 Hz, 2H), 7.22 (t, *J* = 7.6 Hz, 2H), 7.19 (t, *J* = 7.3 Hz, 1H), 6.46 (d, *J* = 15.9 Hz, 1H), 6.17 (dd, *J* = 15.9, 6.4 Hz, 1H), 4.38 – 4.36 (m, 1H), 1.26 (d, *J* = 6.4 Hz, 3H).

^{13}C NMR (151 MHz, $CDCl_3$) δ 136.81 (s), 133.68 (s), 129.50 (s), 128.70 (s), 127.75 (s), 126.57 (s), 69.05 (s), 23.54 (s).

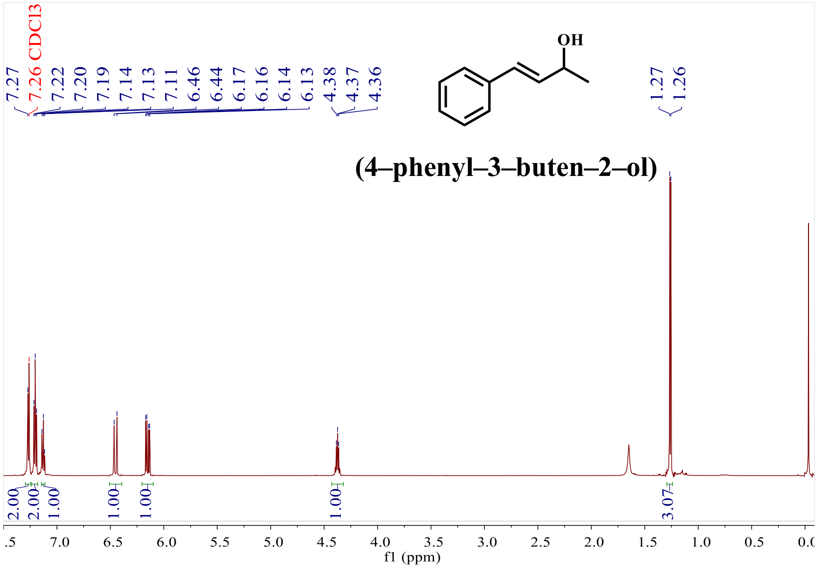

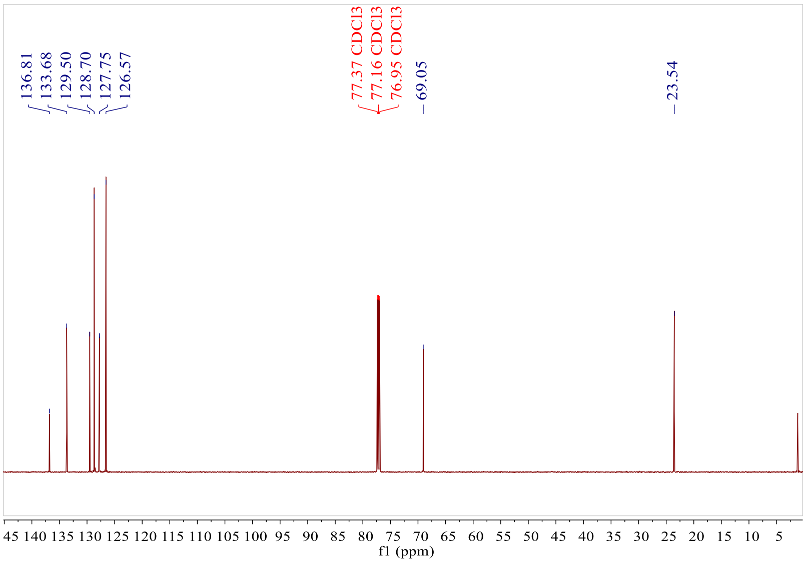

1–(furan–2–yl)ethan–1–ol: ^{1}H NMR (600 MHz, $CDCl_3$) δ 7.36 (d, J = 1.7 Hz, 1H), 6.32 (dd, J = 3.2, 1.8 Hz, 1H), 6.21 (d, J = 3.2 Hz, 1H), 4.86 (q, J = 6.6 Hz, 1H), 1.53 (d, J = 6.6 Hz, 3H).

^{13}C NMR (151 MHz, $CDCl_3$) δ 157.69 (s), 141.99 (s), 110.22 (s), 105.21 (s), 63.69 (s), 21.35 (s).

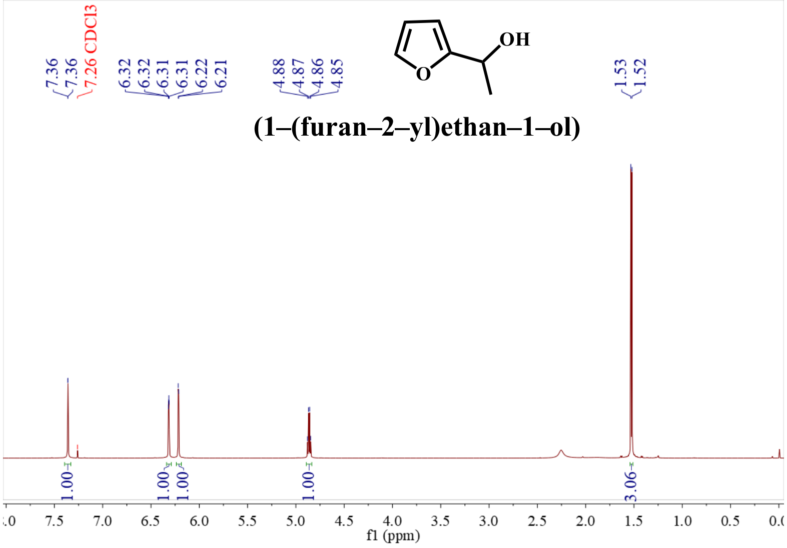

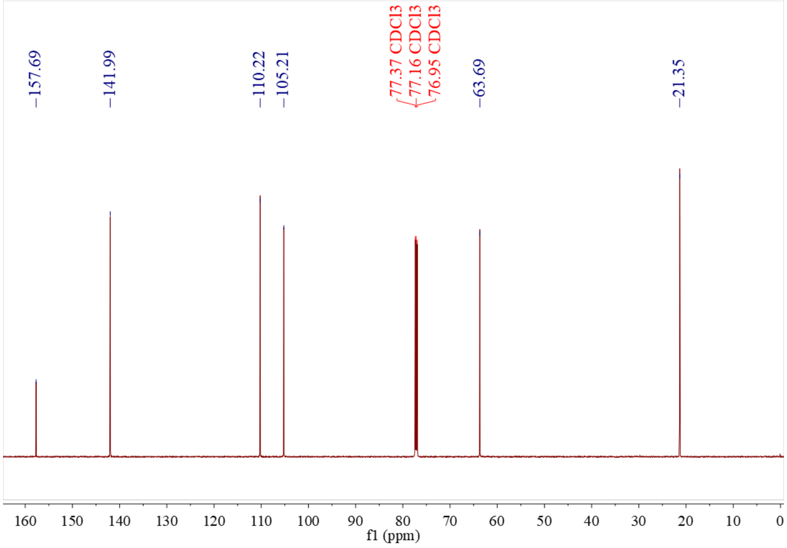

1–(3,5–bis(trifluoromethyl)phenyl)ethan–1–ol: ^{1}H NMR (600 MHz, $CDCl_3$) δ 7.84 (s, 2H), 7.79 (s, 1H), 5.05 (q, J = 6.4 Hz, 1H), 2.01 (s, 1H), 1.55 (d, J = 6.5 Hz, 3H).

^{13}C NMR (151 MHz, $CDCl_3$) δ 148.37 (s), 132.01 (s), 125.81 (s), 122.60 (s), 121.45 (s), 69.43 (s), 25.74 (s).

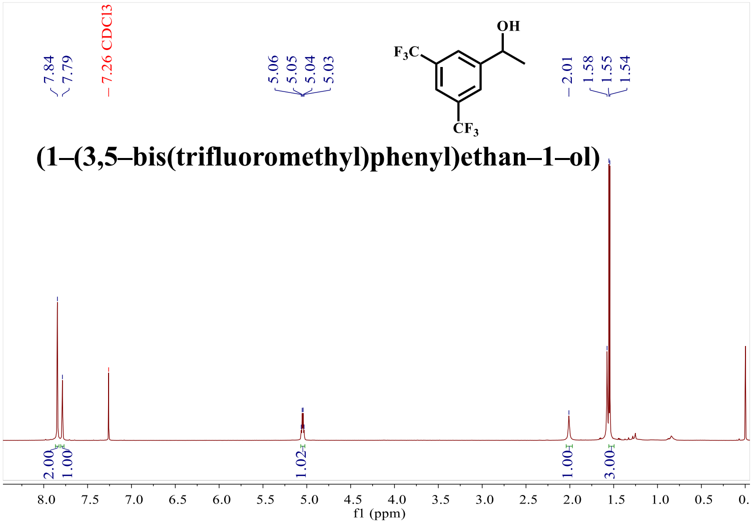

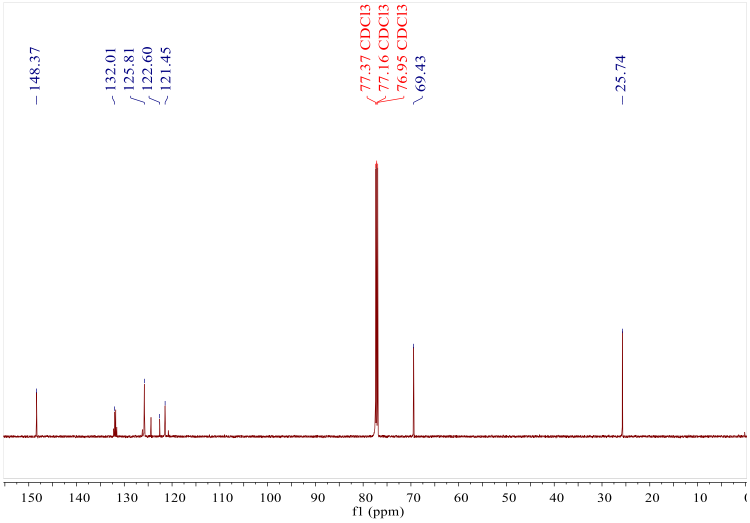

Section 9.2. NMR spectra of products

(*R,E*)–4–phenylbut-3–en–2–yl acetate (1): ^{1}H NMR (600 MHz, $CDCl_3$) δ 7.38 (d, J = 8.6 Hz, 2H), 7.32 (t, J = 7.6 Hz, 2H), 7.24 (d, J = 8.5 Hz, 1H), 6.59 (d, J = 15.9 Hz, 1H), 6.18 (dd, J = 16.0, 6.8 Hz, 1H), 5.51 (p, J = 7.6 Hz, 1H), 2.08 (s, 3H), 1.41 (d, J = 6.5 Hz, 3H).

^{13}C NMR (151 MHz, $CDCl_3$) δ 170.51 (s), 136.46 (s), 131.68 (s), 128.94 (s), 128.71 (s), 128.05 (s), 126.70 (s), 71.15 (s), 21.56 (s), 20.53 (s).

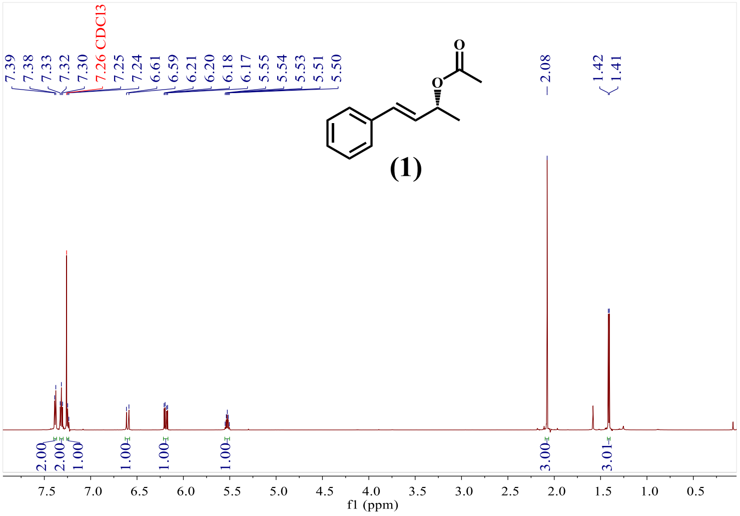

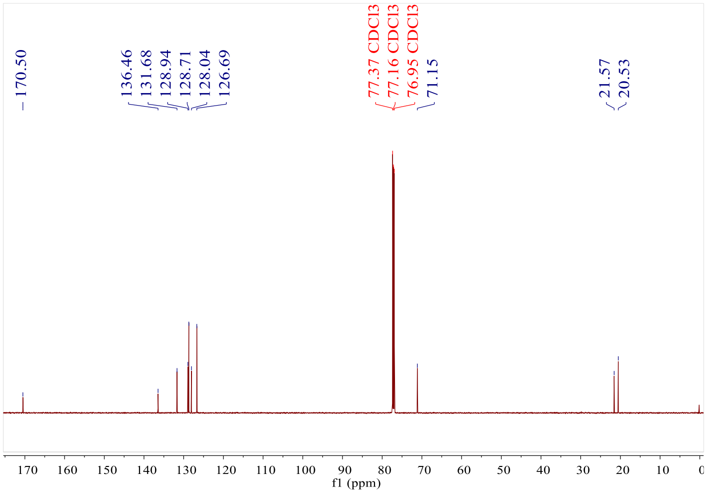

(*R*)–1–phenylethyl acetate (2): ^{1}H NMR (600 MHz, $CDCl_3$) δ 7.39 – 7.29 (m, 5H), 5.90 (q, *J* = 6.6 Hz, 1H), 2.09 (s, 3H), 1.55 (d, *J* = 6.6 Hz, 3H).

^{13}C NMR (151 MHz, $CDCl_3$) δ 170.30 (s), 141.73 (s), 128.53 (s), 127.90 (s), 126.14 (s), 72.33 (s), 22.25 (s), 21.36 (s).

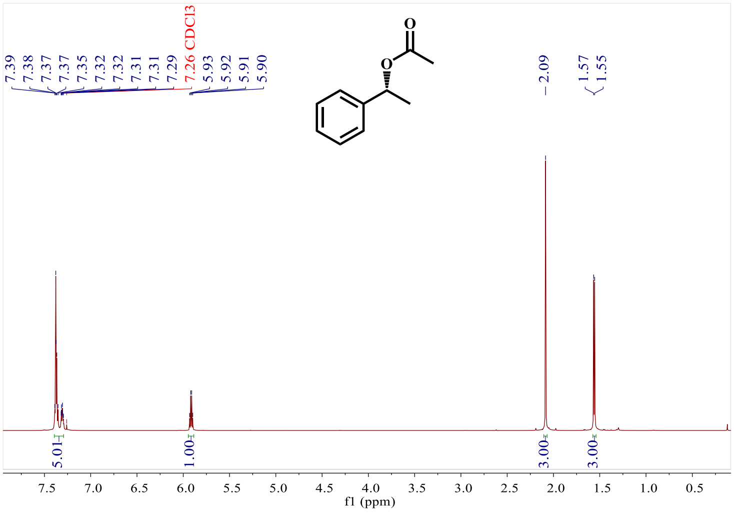

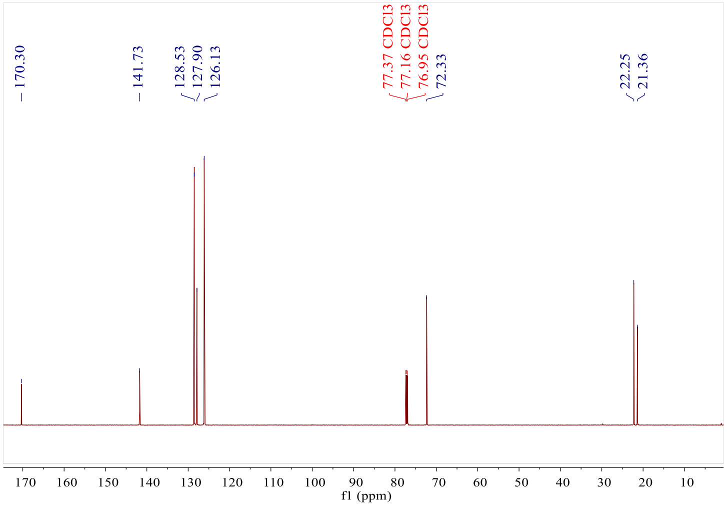

(*R*)–2,3–dihydro–1H–inden–1–yl acetate (3): ^{1}H NMR (600 MHz, $CDCl_3$) δ 7.36 (d, *J* = 7.5 Hz, 1H), 7.25 – 7.21 (m, 2H), 7.18 (t, *J* = 6.9 Hz, 1H), 6.15 (dd, *J* = 7.0, 3.7 Hz, 1H), 3.09 – 3.04 (m, 1H), 2.86 – 2.81 (m, 1H), 2.44 (dt, *J* = 15.1, 6.7 Hz, 1H), 2.08 – 2.04 (m, 1H), 2.02 (d, *J* = 12.3 Hz, 3H).

^{13}C NMR (151 MHz, $CDCl_3$) δ 171.17 (s), 144.50 (s), 141.16 (s), 129.04 (s), 126.80 (s), 125.65 (s), 124.90 (s), 78.45 (s), 32.40 (s), 30.30 (s), 21.41 (s).

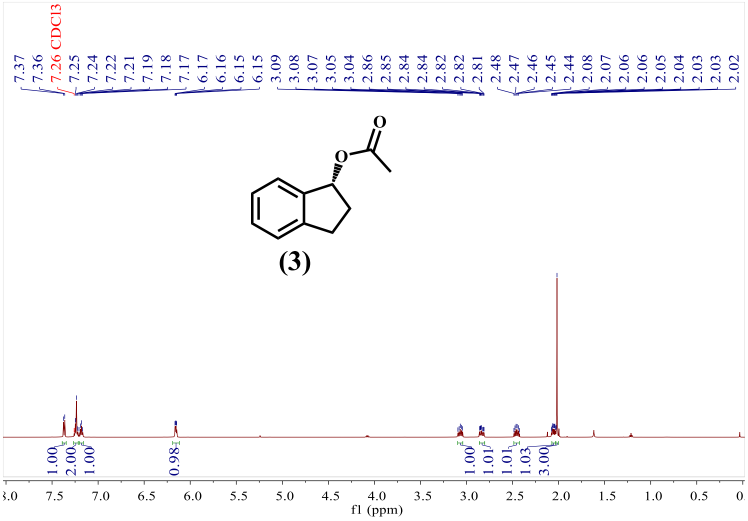

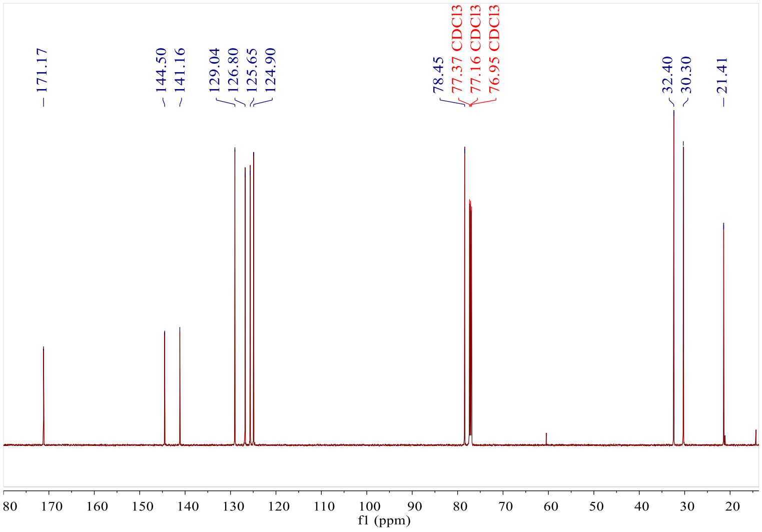

(*R*)–1,2,3,4–tetrahydronaphthalen–1–yl acetate (4): ^{1}H NMR (600 MHz, $CDCl_3$) δ 7.26 (d, *J* = 7.6 Hz, 1H), 7.19 (t, *J* = 6.8 Hz, 1H), 7.14 (t, *J* = 7.2 Hz, 1H), 7.09 (d, *J* = 7.5 Hz, 1H), 5.98 (t, *J* = 4.3 Hz, 1H), 2.86 – 2.82 (m, 1H), 2.70 (dd, *J* = 16.1, 9.4 Hz, 1H), 2.06 (s, 3H), 1.99 – 1.91 (m, 3H), 1.79 (dd, *J* = 14.7, 9.1 Hz, 1H).

^{13}C NMR (151 MHz, $CDCl_3$) δ 170.80 (s), 137.97 (s), 134.63 (s), 129.49 (s), 129.14 (s), 128.14 (s), 126.13 (s), 70.05 (s), 29.16 (s), 29.04 (s), 21.53 (s), 18.88 (s).

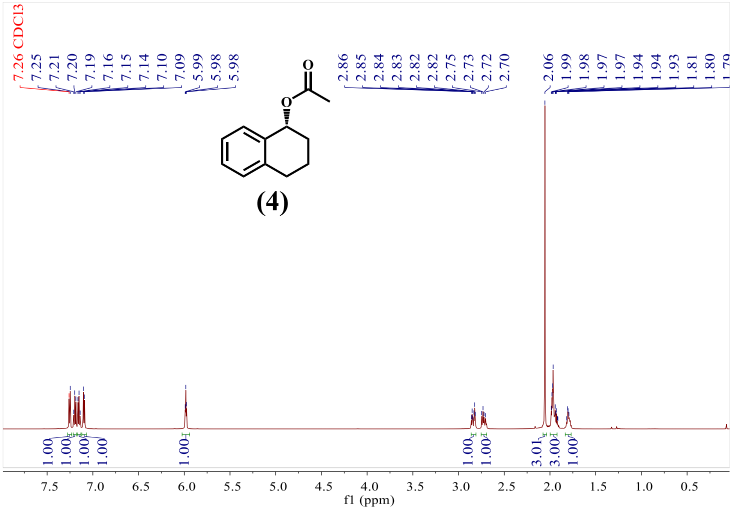

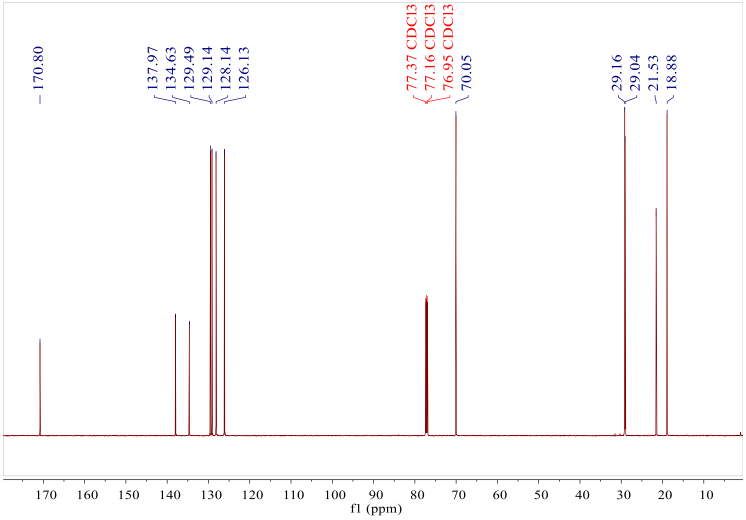

(*R*)–1–(furan–2–yl)ethyl acetate (5): ^{1}H NMR (600 MHz, $CDCl_3$) δ 7.38 (s, 1H), 6.32 (dd, J = 9.6, 4.1 Hz, 2H), 5.95 (q, J = 6.7 Hz, 1H), 2.05 (s, 3H), 1.57 (d, J = 6.7 Hz, 3H).

^{13}C NMR (151 MHz, $CDCl_3$) δ 170.33 (s), 153.51 (s), 142.55 (s), 110.27 (s), 107.83 (s), 65.12 (s), 21.27 (s), 18.28 (s).

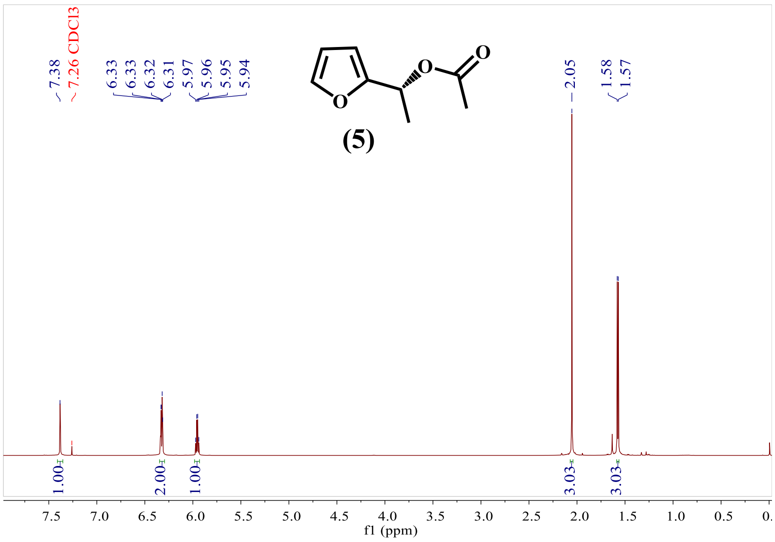

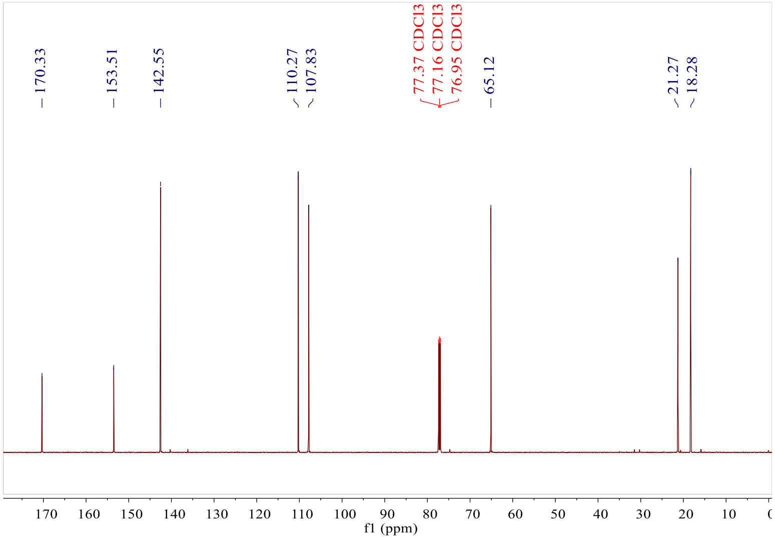

(*R*)–1–(3,5–bis(trifluoromethyl)phenyl)ethyl acetate (6): ^{1}H NMR (600 MHz, $CDCl_3$) δ 7.79 (d, *J* = 11.6 Hz, 3H), 5.94 (q, *J* = 6.7 Hz, 1H), 2.12 (s, 3H), 1.57 (d, *J* = 6.7 Hz, 3H).

^{13}C NMR (151 MHz, $CDCl_3$) δ 170.10 (s), 144.57 (s), 132.22 (s), 132.00 (s), 126.41 (s), 124.27 (s), 122.46 (s), 122.01 (s), 71.08 (s), 22.37 (s), 21.19 (s).

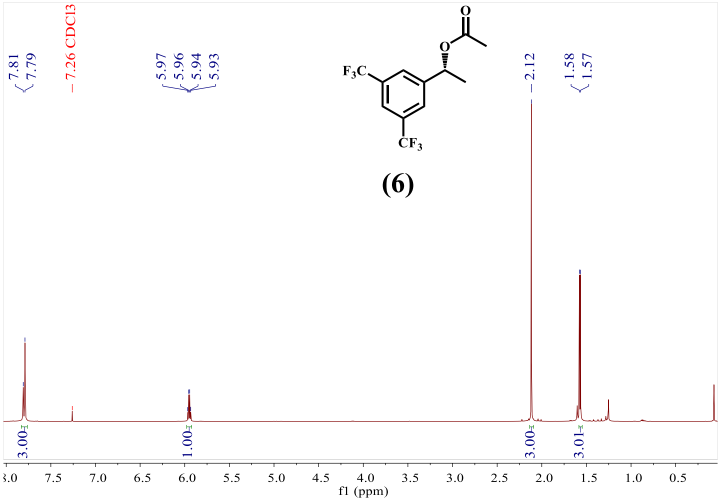

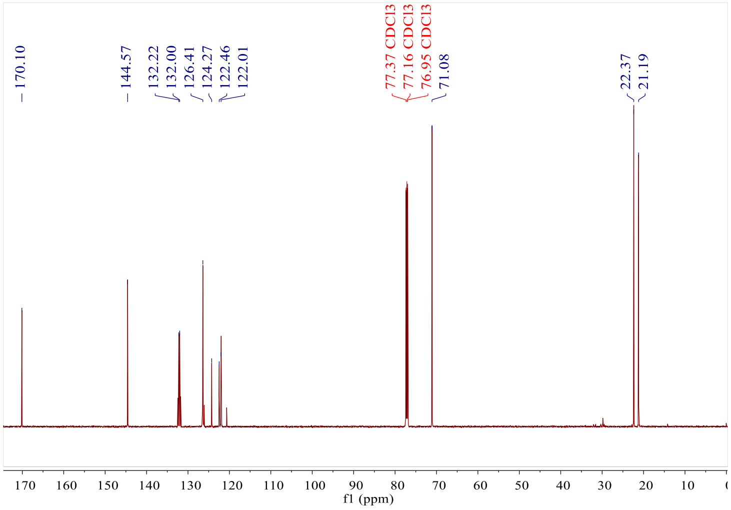

(*R*)–1–(naphthalen–2–yl)ethyl acetate (7): ^{1}H NMR (600 MHz, $CDCl_3$) δ 8.09 (d, *J* = 8.4 Hz, 1H), 7.88 (d, *J* = 7.7 Hz, 1H), 7.81 (d, *J* = 8.2 Hz, 1H), 7.61 (d, *J* = 7.1 Hz, 1H), 7.52 (ddd, *J* = 21.0, 15.3, 10.8 Hz, 3H), 6.66 (q, *J* = 6.6 Hz, 1H), 2.13 (s, 3H), 1.71 (d, *J* = 6.6 Hz, 3H).

^{13}C NMR (151 MHz, $CDCl_3$) δ 170.41 (s), 137.48 (s), 133.88 (s), 130.31 (s), 128.98 (s), 128.52 (s), 126.38 (s), 125.74 (s), 125.43 (s), 123.21 (d, *J* = 5.6 Hz), 69.51 (s), 21.77 (s), 21.44 (s).

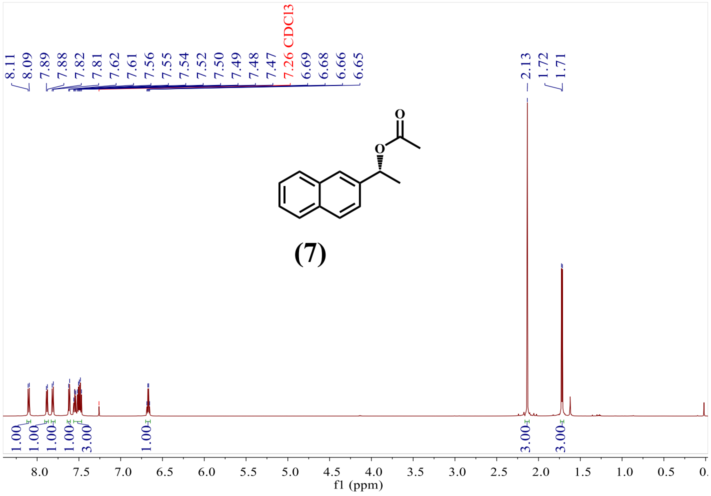

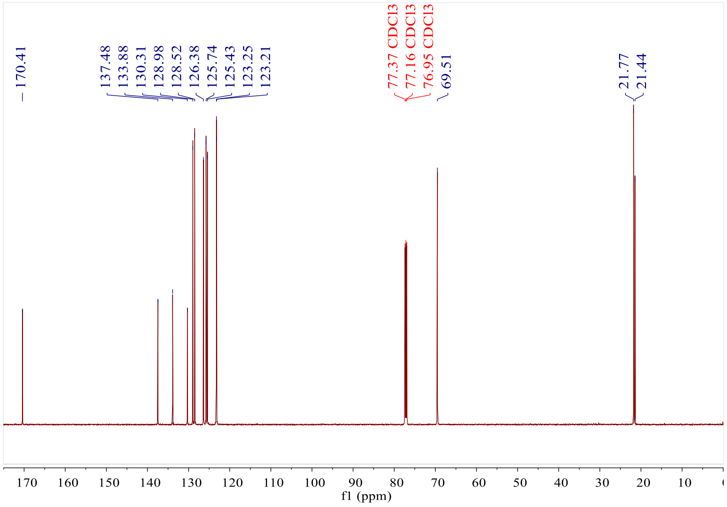

(*R*)–1–(naphthalen–1–yl)ethyl acetate (8): ^{1}H NMR (600 MHz, $CDCl_3$) δ 7.84 (t, *J* = 11.3 Hz, 4H), 7.49 (d, *J* = 6.7 Hz, 3H), 6.05 (q, *J* = 6.6 Hz, 1H), 2.11 (s, 3H), 1.63 (d, *J* = 6.6 Hz, 3H).

^{13}C NMR (151 MHz, $CDCl_3$) δ 170.47 (s), 139.09 (s), 133.26 (s), 133.11 (s), 128.46 (s), 128.12 (s), 127.76 (s), 126.33 (s), 126.16 (s), 125.12 (s), 124.19 (s), 72.55 (s), 22.30 (s), 21.51 (s).

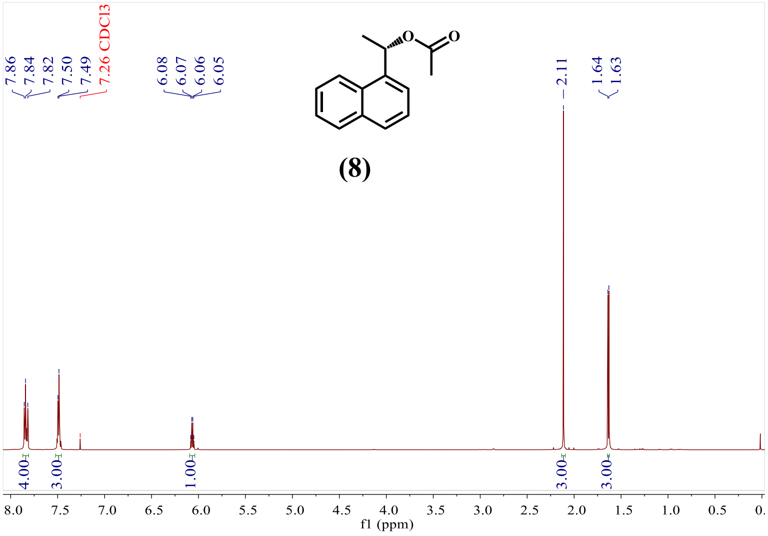

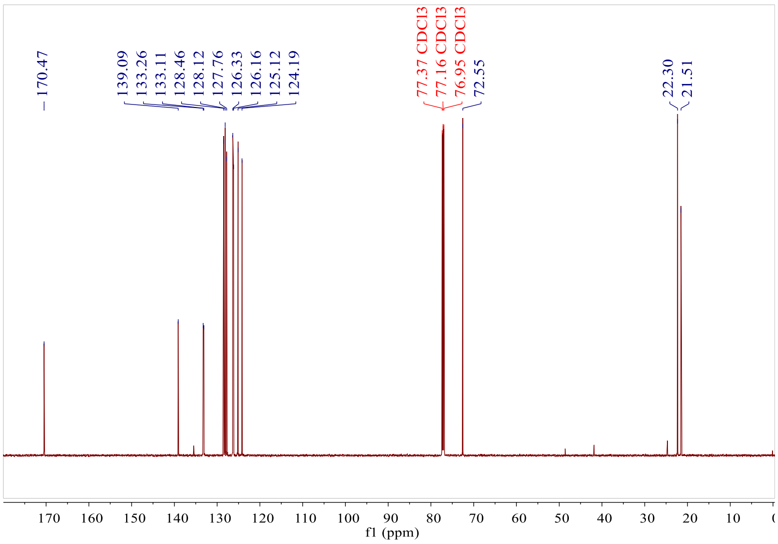

Section 10. HPLC Traces

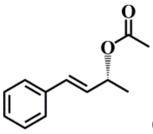

(*R,E*)–4–phenylbut–3–en–2–yl acetate (1): The enantiomeric excess was determined by HPLC with Chiralpack OD–H column at 254 nm; eluent: hexane, flow rate = 1 mL/min, t_{major} = 22.353 min, t_{minor} = 25.635 min.

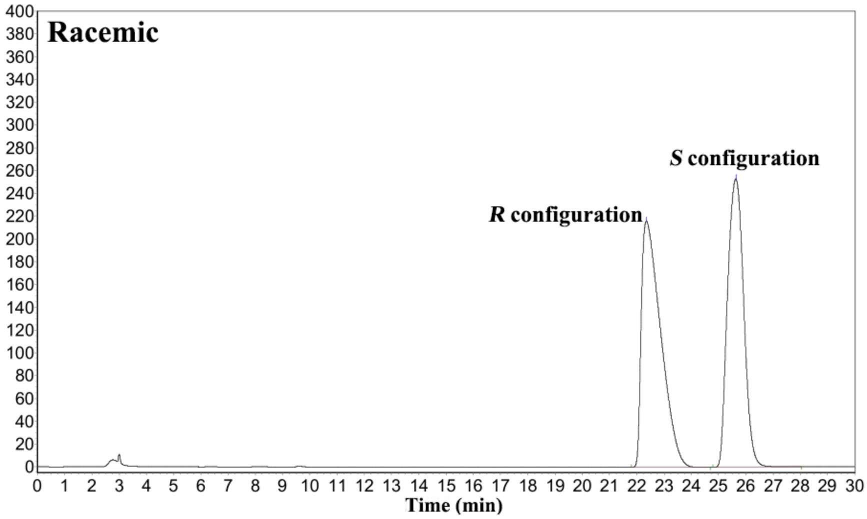

No.	Retention Time	Area	Height	Concentration
**1**	22.353	10692601.00	216192.172	50.6397
**2**	25.635	10422461.00	253029.563	49.3603

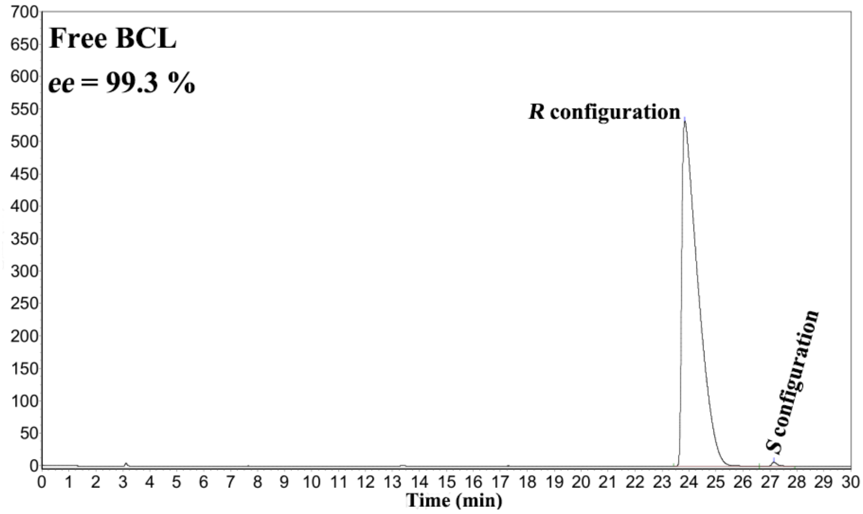

No.	Retention Time	Area	Height	Concentration
**1**	23.850	22182548.000	531686.938	99.6358
**2**	27.143	81074.789	6020.103	0.3642

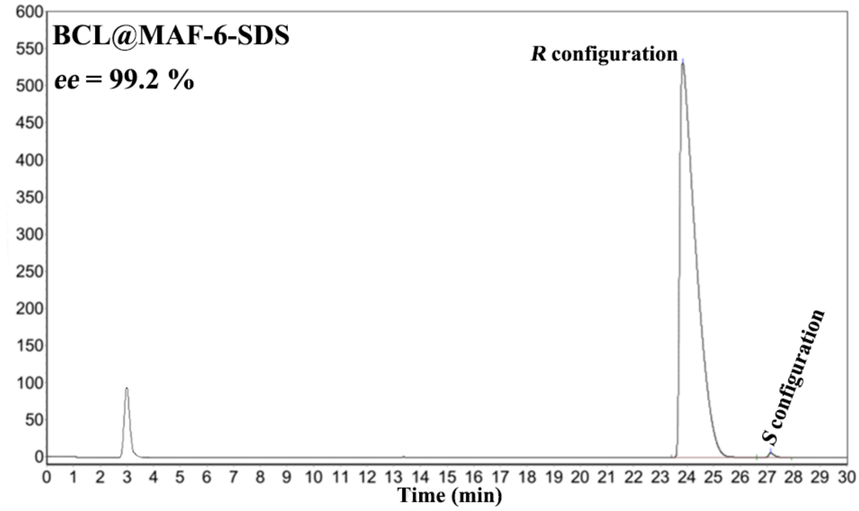

No.	Retention Time	Area	Height	Concentration
**1**	23.855	22082149.500	530886.961	99.6232
**2**	27.153	81885.373	5927.157	0.3768

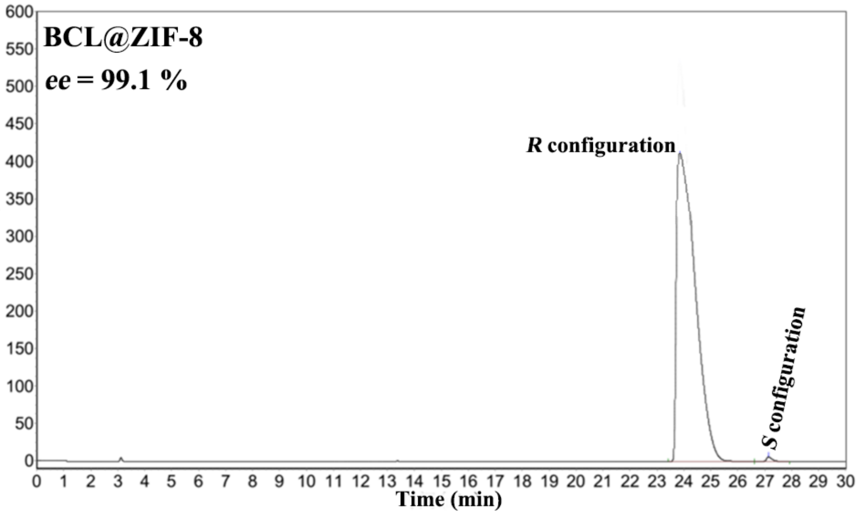

No.	Retention Time	Area	Height	Concentration
**1**	23.840	18082149.000	387286.961	99.5534
**2**	27.141	80975.580	5913.327	0.4466

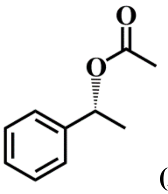

(*R*)–1–phenylethyl acetate (2): The enantiomeric excess was determined by HPLC with Chiralpack OD–H column at 216 nm; eluent: hexane: *i*-PrOH (99: 1), flow rate = 1 mL/min, t_{major} = 6.380 min, t_{minor} = 7.133 min.

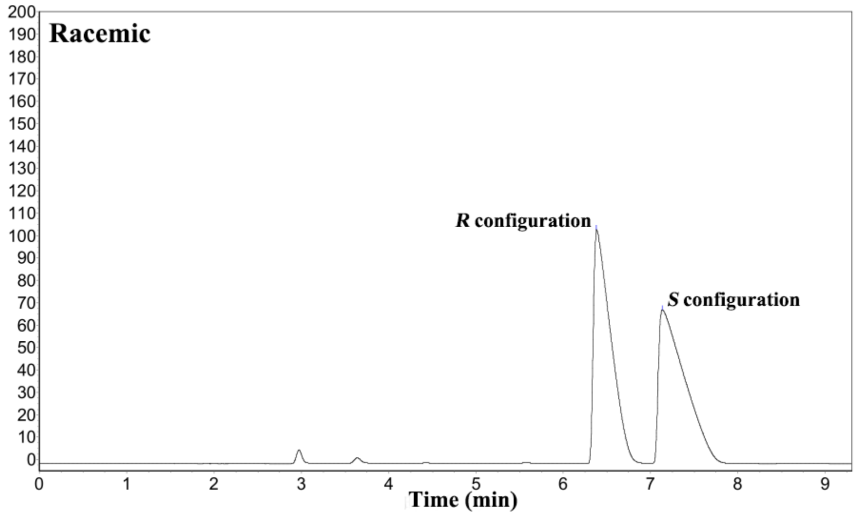

No.	Retention Time	Area	Height	Concentration
**1**	6.380	1435842.875	235423.938	49.9786
**2**	7.133	1437127.375	194166.188	50.0214

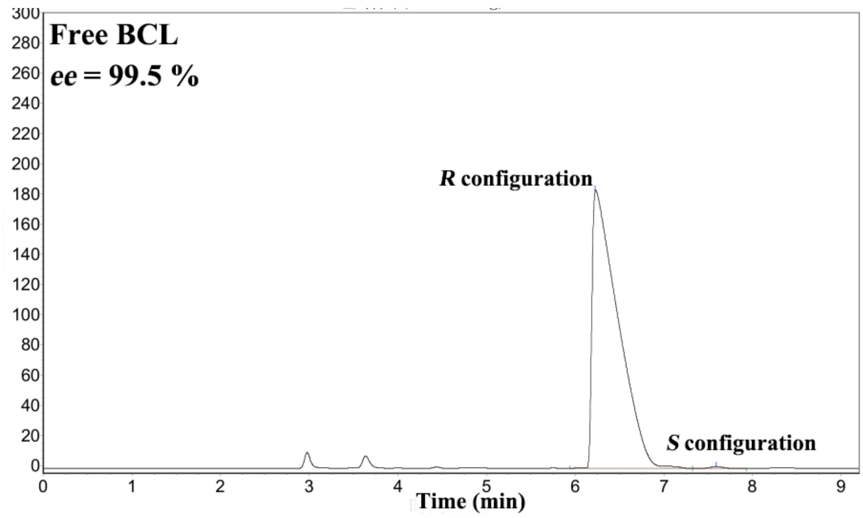

No.	Retention Time	Area	Height	Concentration
**1**	6.227	3735436.750	184396.484	99.6783
**2**	7.590	12053.653	1237.351	0.3217

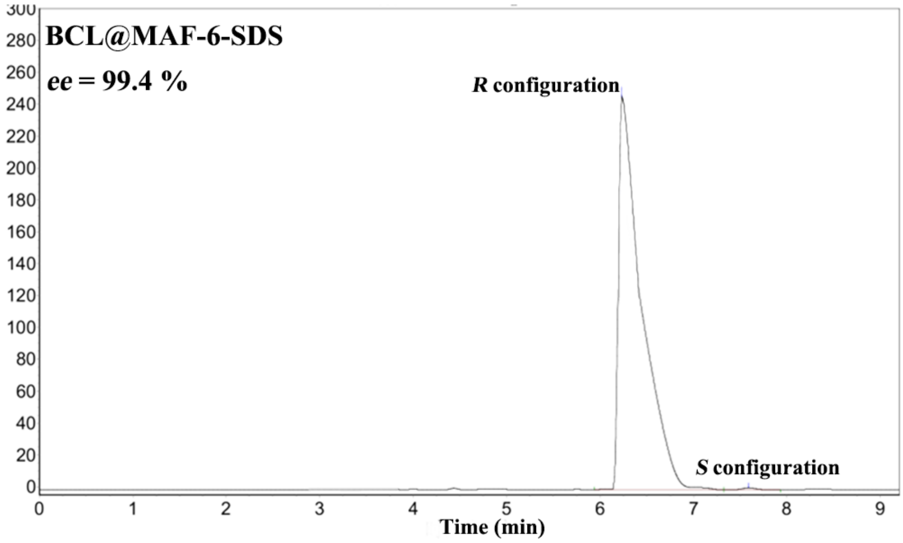

No.	Retention Time	Area	Height	Concentration
**1**	6.212	5037563.000	202369.387	99.7508
**2**	7.588	12582.559	1289.562	0.2492

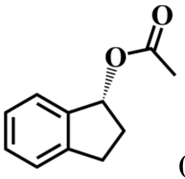

(*R*)–2,3–dihydro–1H–inden–1–yl acetate (3): The enantiomeric excess was determined by HPLC with Chiralpack OD–H column at 254 nm; eluent: hexane: *i*-PrOH (99: 1), flow rate = 0.3 mL/min, tmajor = 19.562 min, tminor = 21.125 min.

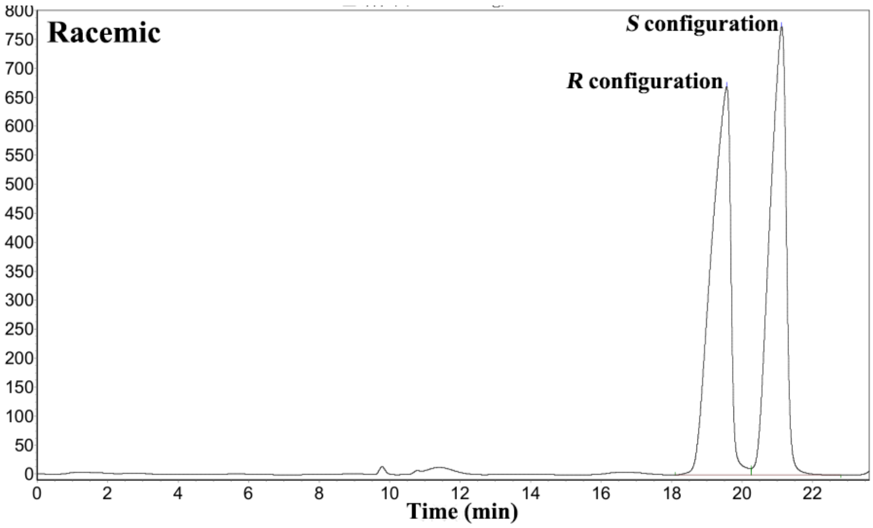

No.	Retention Time	Area	Height	Concentration
**1**	19.562	25695030.000	669571.063	50.5618
**2**	21.125	25052850.000	773564.875	49.4382

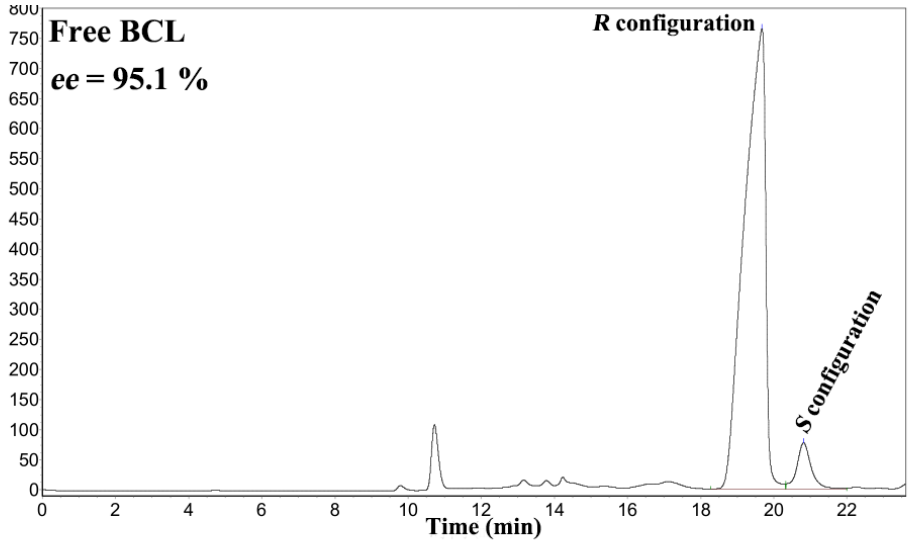

No.	Retention Time	Area	Height	Concentration
**1**	19.675	26439937.206	784186.343	97.5565
**2**	20.817	1358510.121	219831.959	2.4435

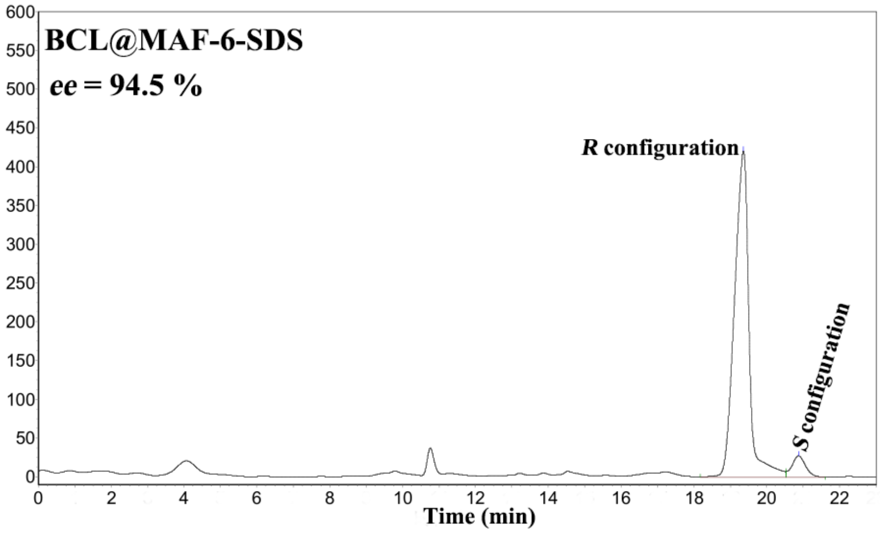

No.	Retention Time	Area	Height	Concentration
**1**	19.357	11656437.000	420962.938	97.2742
**2**	20.873	326632.625	17701.803	2.7258

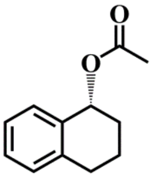

(*R*)–1,2,3,4–tetrahydronaphthalen–1–yl acetate (4): The enantiomeric excess was determined by HPLC with Chiralpack OJ–H column at 254 nm; eluent: hexane: *i*-PrOH (99: 1), flow rate = 0.5 mL/min, t_{major} = 9.632 min, t_{minor} = 10.910 min.

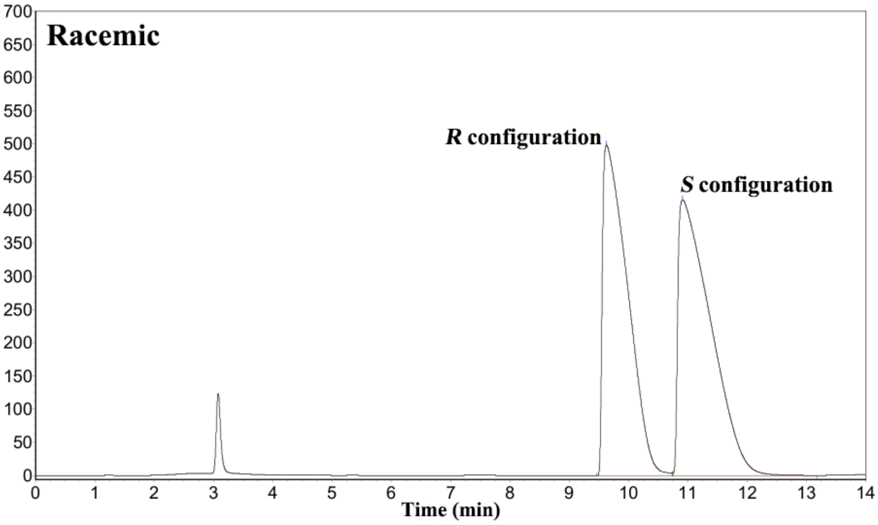

No.	Retention Time	Area	Height	Concentration
**1**	9.632	14569244.000	498230.750	47.8479
**2**	10.910	15884527.000	415885.500	52.1521

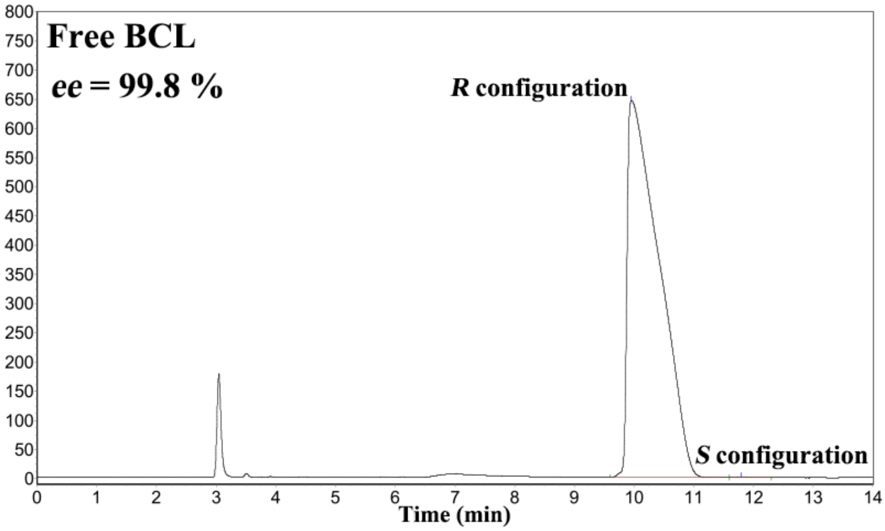

No.	Retention Time	Area	Height	Concentration
**1**	9.955	23946898.000	645917.563	99.9159
**2**	11.793	20167.313	1479.504	0.0841

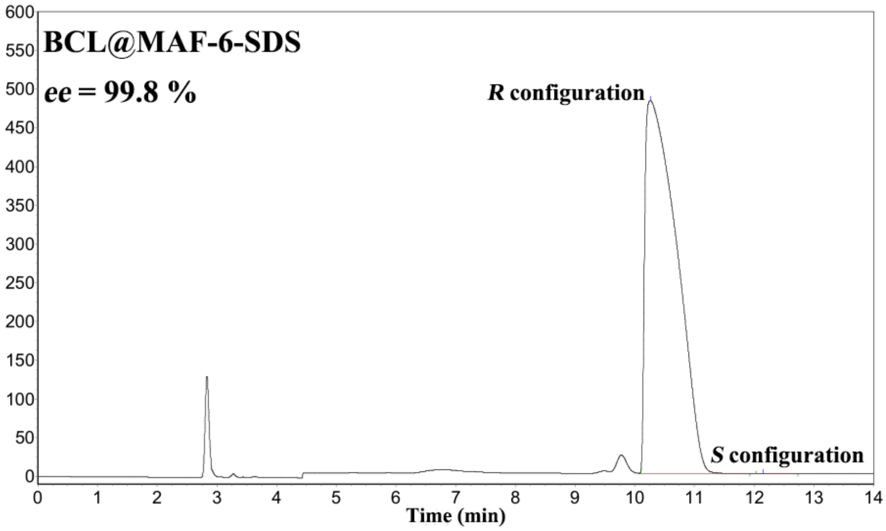

No.	Retention Time	Area	Height	Concentration
**1**	10.263	17687448.000	481692.563	99.9184
**2**	12.148	14449.228	182.312	0.0816

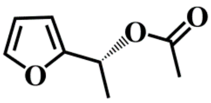

(*R*)–1–(furan–2–yl)ethyl acetate (5): The enantiomeric excess was determined by HPLC with Chiralpack AD–H column at 220 nm; eluent: hexane: *i*-PrOH (99: 1), flow rate = 0.3 mL/min, t_{major} = 18.478 min, t_{minor} = 19.578 min.

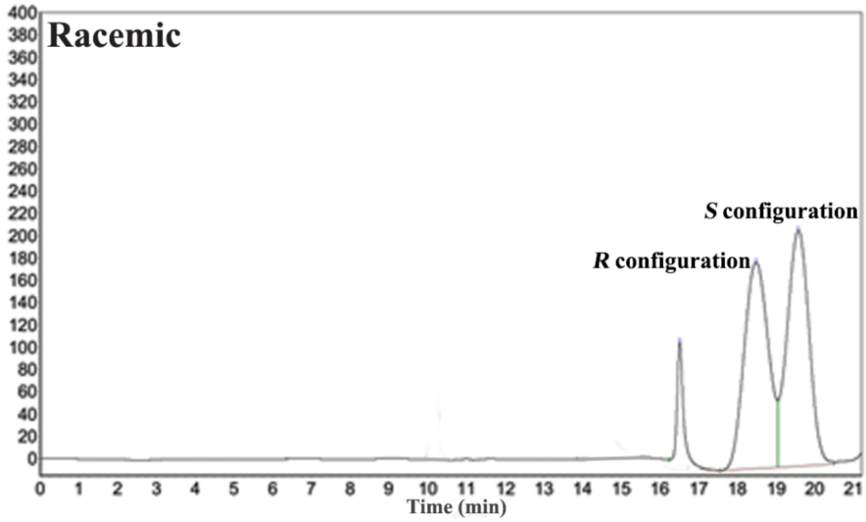

No.	Retention Time	Area	Height	Concentration
**1**	18.478	8058348.500	184935.969	48.7056
**2**	19.578	8398064.000	211110.234	52.2944

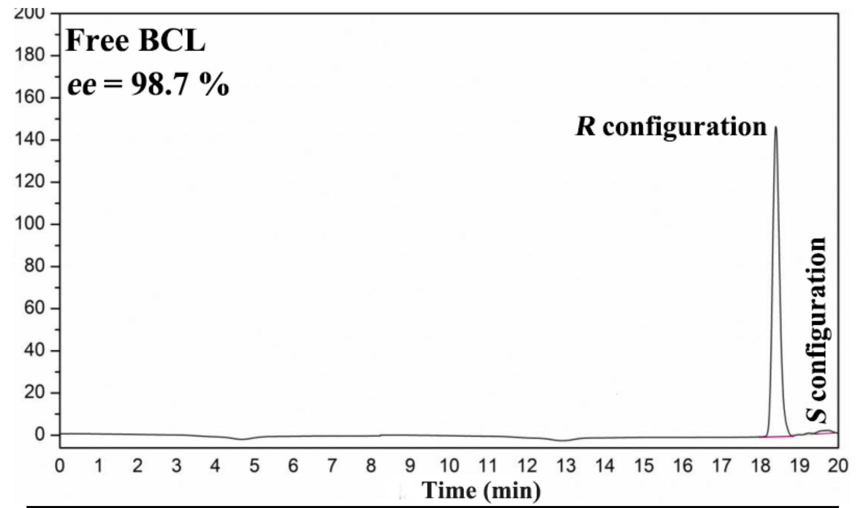

No.	Retention Time	Area	Height	Concentration
**1**	18.398	1863097.625	147065.250	99.3502
**2**	19.248	12186.170	1559.346	0.6498

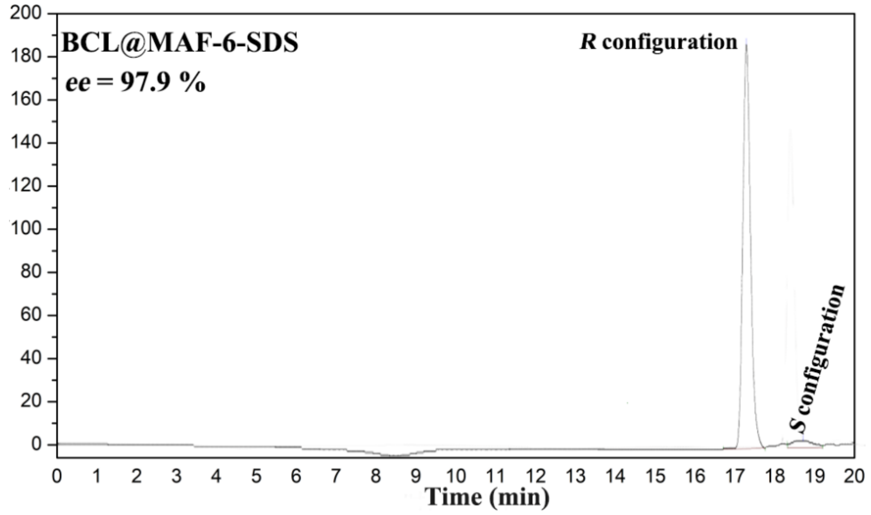

No.	Retention Time	Area	Height	Concentration
**1**	17.283	2062061.250	166525.906	98.9384
**2**	18.662	22126.297	1968.065	1.0616

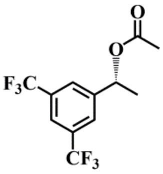

(*R*)–1–(3,5–bis(trifluoromethyl)phenyl)ethyl acetate (6): The enantiomeric excess was determined by HPLC with Chiralpack OD–H column at 265 nm; eluent: hexane: *i*-PrOH (98: 2), flow rate = 1 mL/min, t_{major} = 2.982 min, t_{minor} = 3.375 min.

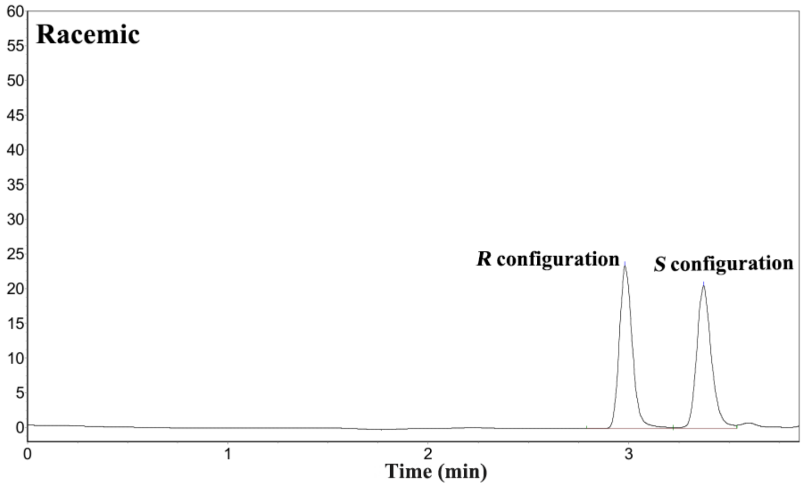

No.	Retention Time	Area	Height	Concentration
**1**	2.982	106287.234	23462.074	50.0089
**2**	3.375	106249.461	20595.346	49.9911

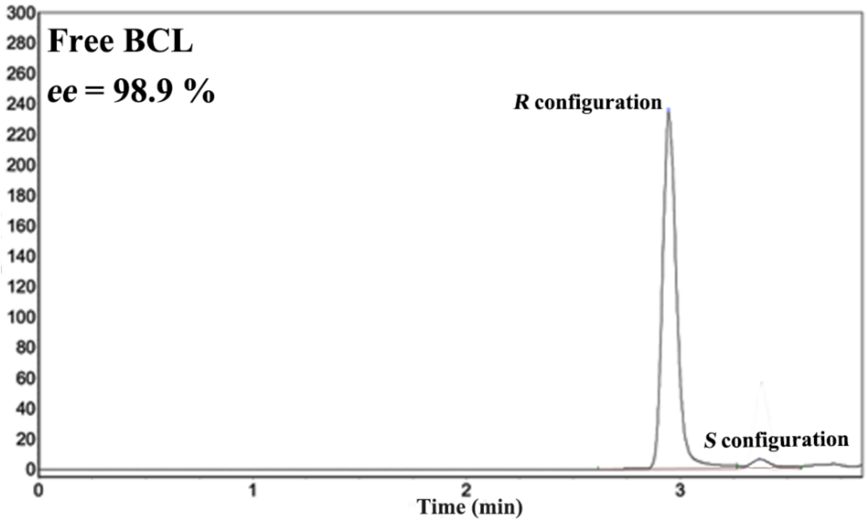

No.	Retention Time	Area	Height	Concentration
**1**	2.945	1082487.125	234270.172	99.4618
**2**	3.380	5857.200	1155.171	0.5382

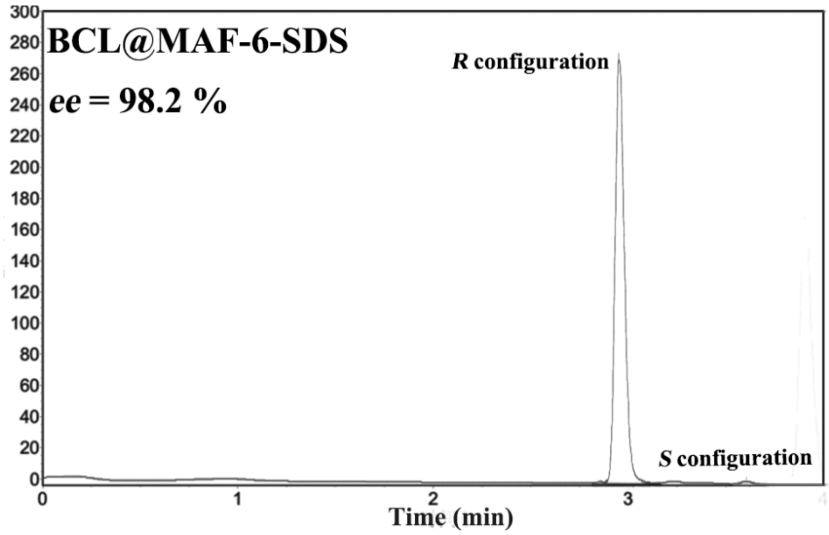

No.	Retention Time	Area	Height	Concentration
**1**	2.885	983891.750	197661.734	99.1524
**2**	3.688	9112.506	4831.987	0.8476

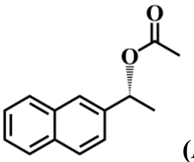

(*R*)–1–(naphthalen–2–yl)ethyl acetate (7): The enantiomeric excess was determined by HPLC with Chiralpack OD–H column at 254 nm; eluent: hexane: *i*-PrOH (95: 5), flow rate = 0.8 mL/min, t_{major} = 6.463 min, t_{minor} = 7.323 min.

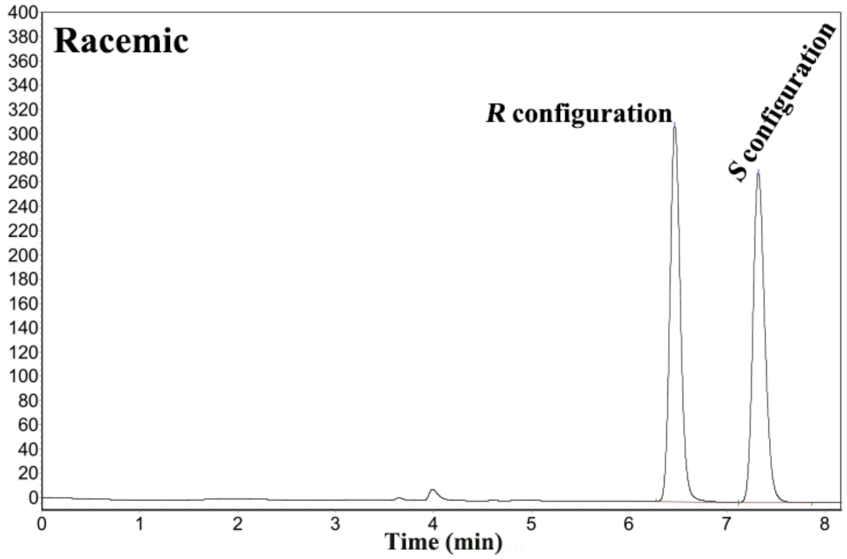

No.	Retention Time	Area	Height	Concentration
**1**	6.463	2304109.000	309279.813	50.1389
**2**	7.323	2291343.500	271152.688	49.8611

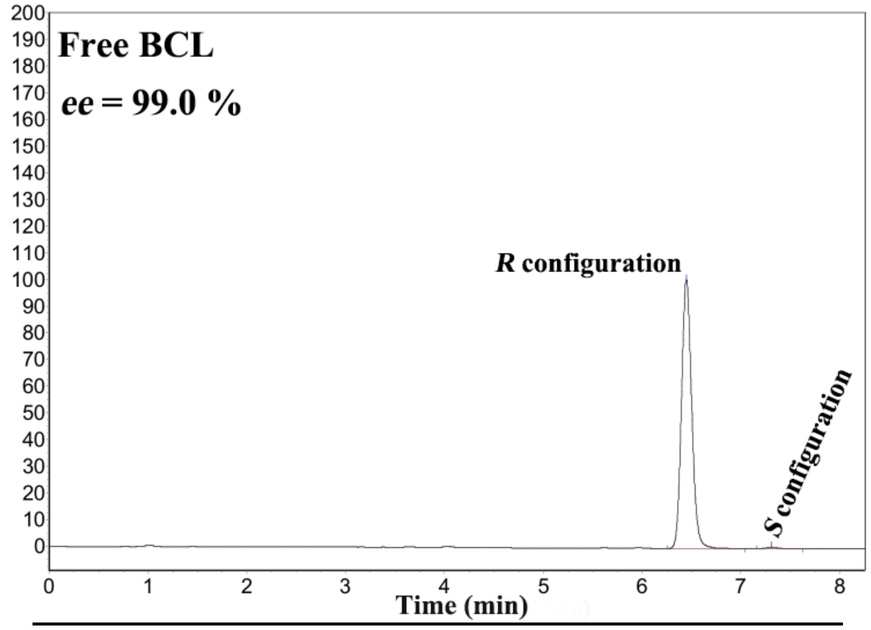

No.	Retention Time	Area	Height	Concentration
**1**	6.450	723789.875	100652.102	99.5149
**2**	7.308	3528.299	579.645	0.4851

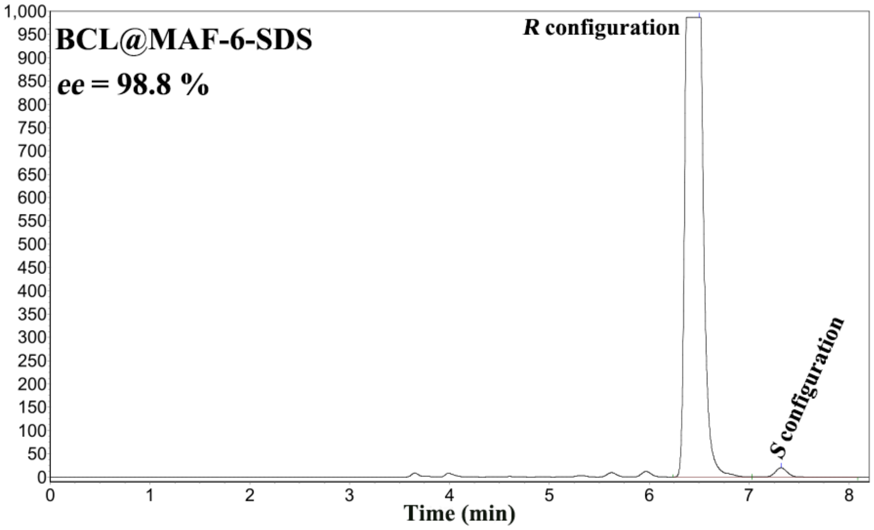

No.	Retention Time	Area	Height	Concentration
**1**	6.502	12779619.000	987892.750	99.3895
**2**	7.323	78496.641	10660.996	0.6105

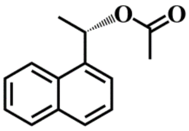

(*R*)–1–(naphthalen–1–yl)ethyl acetate (8): The enantiomeric excess was determined by HPLC with Chiralpack AD–H column at 254 nm; eluent: hexane: *i*-PrOH (99: 1), flow rate = 0.5 mL/min, t_{major} = 12.717 min, t_{minor} = 14.655 min.

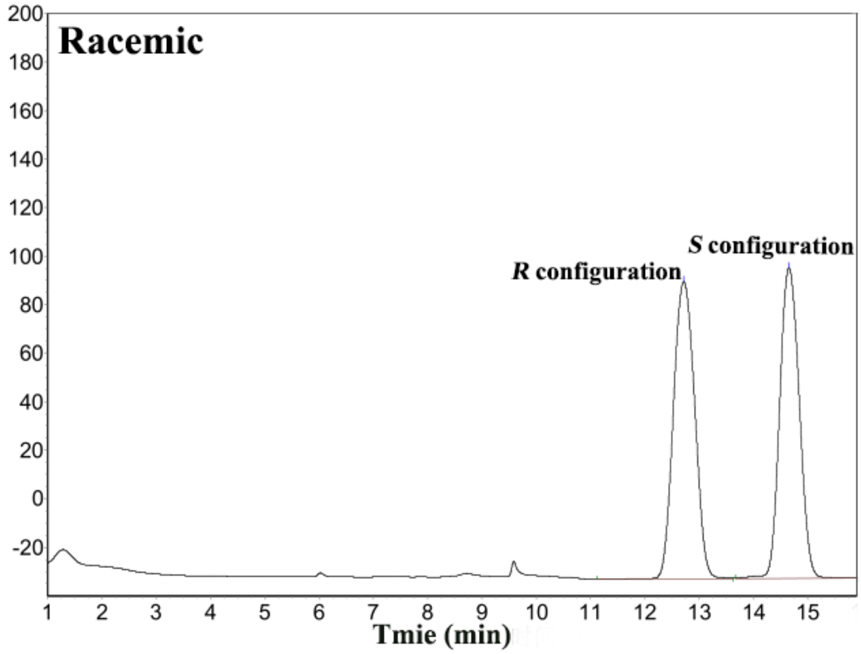

No.	Retention Time	Area	Height	Concentration
**1**	12.717	3344731.250	122699.781	50.9988
**2**	14.655	3213723.000	128153.406	49.0012

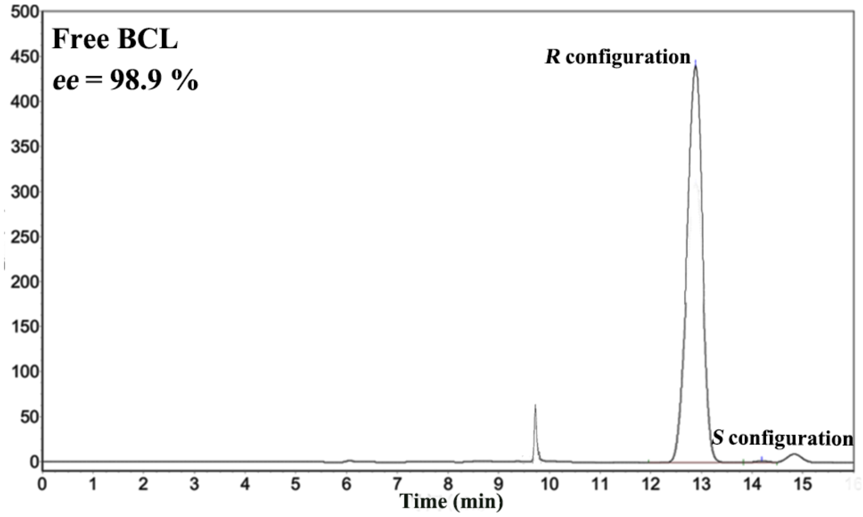

No.	Retention Time	Area	Height	Concentration
**1**	12.912	9795764.000	376433.000	99.4709
**2**	14.832	52103.441	2310.656	0.5291

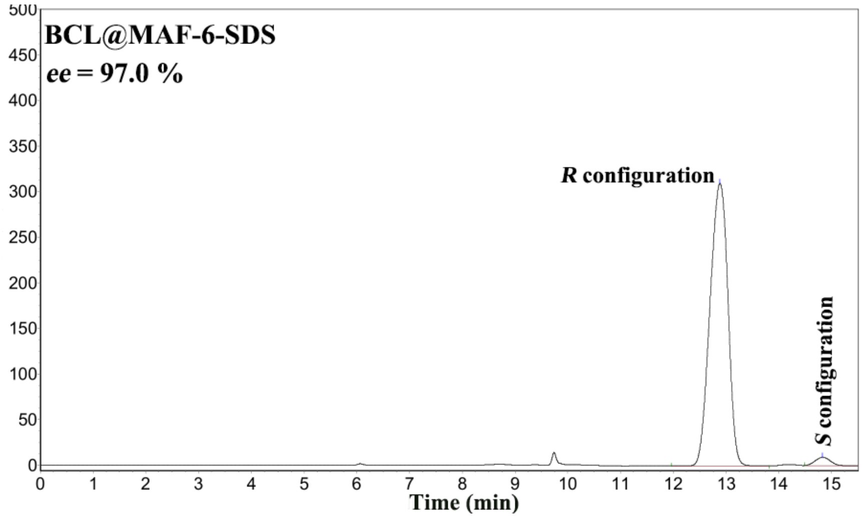

No.	Retention Time	Area	Height	Concentration
**1**	12.885	7335746.500	309994.969	98.5093
**2**	14.828	211013.141	9519.868	1.4907

Section 11. References

(1) Leśniarek A.; Chojnacka A.; Gładkowski W. Application of lecitase (R) ultra-catalyzed hydrolysis to the kinetic resolution of (E)-4-phenylbut-3-en-2-yl esters. *Catalysts* **2018**, *8*, 423.

(2) Onaran, M. B.; Seto, C. T. Using a lipase as a high-throughput screening method for measuring the enantiomeric excess of allylic acetates. *J. Org. Chem.* **2003**, *68*, 8136–8141.

(3) McCarney, E. R.; Breaux, C. J.; Rendle, P. M. Measurement of the hydrodynamic radii of PEE-G dendrons by diffusion spectroscopy on a benchtop NMR spectrometer. *Magn. Reson. Chem.* **2020**, *58*, 641–647.

(4) Li, D.; Keresztes, I.; Hopson, R.; Williard, P. G. Characterization of reactive intermediates by multinuclear diffusion-ordered NMR spectroscopy (DOSY). *Acc. Chem. Res.* **2009**, *42*, 270–280.

(5) Li, Y. M.; Yuan, J.; Ren, H.; Ji, C. Y.; Tao, Y.; Wu, Y.; Chou, L. Y.; Zhang, Y. B.; Cheng, L. Fine-tuning the micro-environment to optimize the catalytic activity of enzymes immobilized in multivariate metal–organic frameworks. *J. Am. Chem. Soc.* **2021**, *143*, 15378–15390.

(6) Li, P.; Modica, J. A.; Howarth, A. J.; Vargas L., E.; Moghadam, P. Z.; Snurr, R. Q.; Mrksich, M.; Hupp, J. T.; Farha, O. K. Toward design rules for enzyme immobilization in hierarchical mesoporous metal–organic frameworks. *Chem* **2016**, *1*, 154−169.

(7) Bornscheuer, U. T.; Ordoñez, G. R.; Hidalgo, A.; Gollin, A.; Lyon, J.; Hitchman, T. S.; Weiner, D. P. Selectivity of lipases and esterases towards phenol esters. *J. Mol. Catal., B: Enzym.* **2005**, *36*, 8–13.

(8) Białecka-Florjańczyk, E.; Krzyczkowska, J.; Stolarzewicz, I. Catalytic activity of baker's yeast in ester hydrolysis. *Biocatal. Biotransfor.* **2010**, *28*, 288–291.

(9) Ramírez-Montoya, L. A.; Concheso, A.; Alonso-Buenaposada, I. D.; García, H.; Angel Menéndez, J.; Arenillas, A.; Montes-Morán, M. A. Protein adsorption and activity on carbon xerogels with narrow pore size distributions covering a wide mesoporous range. *Carbon* **2017**, *118*, 743–751.

(10) Du, Y.; Gao, J.; Zhou, L.; Ma, L.; He, Y.; Zheng, X.; Huang, Z.; Jiang, Y. MOF-based nanotubes to hollow nanospheres through protein-induced soft-templating pathways. *Adv. Sci.* **2019**, *6*, 1801684.

(11) Feng, D.; Liu, T. F.; Su, J.; Bosch, M.; Wei, Z.; Wan, W.; Yuan, D.; Chen, Y. P.; Wang, X.; Wang, K.; Lian, X.; Gu, Z. Y.; Park, J.; Zou, X.; Zhou, H. C. Stable metal–organic frameworks containing single-molecule traps for enzyme encapsulation. *Nat. Commun.* **2015**, *6*, 5979.
