# Supplementary material for: Highly Enantioselective Catalysis by Enzyme Encapsulated in Metal Azolate Frameworks with Micelle-Controlled Pore Sizes
Source: ACS Cent Sci. 2024 Jan 18;10(2):358–66. doi: 10.1021/acscentsci.3c01432 (PMC10906037; doi:10.1021/acscentsci.3c01432)
Supplement: Supplementary file 2 — oc3c01432_si_002.pdf [file oc3c01432_si_002.pdf]

oc-2023-01432a.R1

Name: Peer Review Information for "Highly Enantioselective Catalysis by Enzyme Encapsulated in Metal Azolate Frameworks with Micelle-Controlled Pore Sizes"

First Round of Reviewer Comments

Reviewer: 1

Comments to the Author

The manuscript, by Yuan, Zhang, and Cheng, reports the chiral catalytic synthesis of pharmaceutical precursors for the first time by enzymes encapsulated in metal azolate frameworks (MAFs), whose pore sizes are controlled through a micelle-directed mechanism in aqueous conditions. They have illustrated the delicate regulation of the micro-environments of enzymes by MOF encapsulation, we the breakthrough here is the enlargement of pore aperture allowing the diffuse in and diffuse out of organic molecules enabling the enantioselctive catalytic syntheses of pharmaceutical precursors in almost quantitative yields. This is a millstone from the proof-of-concept heading to the practical application for enzyme@MOF nanocomposite materials. On the other hand, the molecular insight for the micelles-directed synthesis resembling the formation of mesoporous materials is very inspiring. The overall quality of this experiments is very high and the presentation of the manuscript is scholarly. Considering the novelty and significance, I highly recommend this manuscript to be published in ACS Central Science after minor revision with the considerations provided as follows:

1. The manuscript mentions that a variety of enzymes show good activity in MAF-6. I am curious about the enzymes are embedded in the crystals or loaded into the pores of the framework?

2. MAF-6-SDS showed better catalytic performance over other catalysts in activity tests and kinetic resolution. Does MAF-6-SDS still contain SDS, and if so, what is its content?

3. The hydrolysis activity of BCL/MAF-6 and BCL/MAF-6-SDS should be discussed more in details in the NPB hydrolysis reaction.

4. Whether the enzyme in MAF-6 can still maintain good activity in harsh conditions, such as higher temperatures or organic solvents?

5. For guest-encapsulated MOF catalysis, please include related references: Chin. J. Catal. 2023, 45, 1-5; Natl. Sci. Rev. 2020, 7, 37-45.

Reviewer: 2

Comments to the Author

The present work by Zhang et.al presents an effective approach for the aqueous synthesis of MAF-6 with large pore aperture, enabling the encapsulation of enzyme BCL for subsequent enantioselective catalysis. This biocomposite can efficiently catalyze the synthesis of drug precursor molecules with 94-99% enantioselectivity and nearly quantitative yields. It's an interesting work, and the manuscript is well-organized. I think the paper is suitable for publication after addressing the following comments.

1. In page 4, it is mentioned that "As substrate hydrophobicity increased, transitioning from CH3 to CH(CH3)2, BCL@MAF6-SDS showed a significant 9.2-fold increase in conversion……". Is the hydrophobic difference the only contributing factor? Are there any other factors, such as steric hindrance, that should be considered? Additionally, how can the authors conclude that it's the influence of MAF-6's hydrophobic nature for catalysis? In fact, there are numerous factors associated with MOF that can potentially impact catalysis.

2. After six cycles, the yield of the catalytic reaction is observed to decrease by approximately 20%. What is the reason for this decrease? The absence of any change in crystallinity alone does not suffice to demonstrate the excellent recyclability and reusability of this biocomposite catalyst.

3. What is the advantage of using MAF-6 to encapsulate BCL as this biocomposite does not improve its catalytic effect compared to free BCL?

4. Does the catalytic reaction mechanism of the biocomposite differ from that of BCL?

Author's Response to Peer Review Comments:

December 28th, 2023

Prof. Editor:

Senior Editor, ACS Central Science

Dear Prof. Editor:

In response to your revision request on our manuscript entitled "Highly Enantioselective Catalysis by Enzyme Encapsulated in Metal Azolate Frameworks with Micelle-Controlled Pore Sizes" (oc-2023-01432a), we have submitted our revised manuscript and its revised supporting information, along with a point-by-point author responses to reviewer comments. We highly appreciate your great effort in handling our manuscript, and the insightful comments and constructive suggestions from the reviewers.

Both reviewers are very positive and evaluate our work highly. We have revised the manuscript and the supporting information according to their comments. To facilitate your review, we have highlighted the

revised portions in red throughout the paper. We believe the overall quality of our paper has been therefore enhanced. We sincerely hope that the corrections made align with your expectations.

Should you have any further inquiries or require additional information, please do not hesitate to reach out. We eagerly anticipate your consideration of our revised manuscript.

Thank you once again for your time and valuable input.

Sincerely yours,
